# Distinct and shared genetic architectures of gestational diabetes mellitus and type 2 diabetes

**DOI:** 10.1038/s41588-023-01607-4

**Published:** 2024-01-05

**Authors:** Amanda Elliott, Raymond K. Walters, Matti Pirinen, Mitja Kurki, Nella Junna, Jacqueline I. Goldstein, Mary Pat Reeve, Harri Siirtola, Susanna M. Lemmelä, Patrick Turley, Elisa Lahtela, Juha Mehtonen, Kadri Reis, Abdelrahman G. Elnahas, Anu Reigo, Priit Palta, Tõnu Esko, Reedik Mägi, Estonian Biobank Research Team, Andres Metspalu, Mari Nelis, Lili Milani, Georgi Hudjashov, FinnGen, Haari Siirtola, Elisa Lahtinen, Aarno Palotie, Mark J. Daly, Elisabeth Widén

**Affiliations:** 1 Analytic and Translational Genetics Unit, Massachusetts General Hospital, Boston, MA USA; 2 Stanley Center for Psychiatric Research, Broad Institute of Harvard and MIT, Cambridge, MA USA; 3 Harvard Medical School, Boston, MA USA; 4 Institute for Molecular Medicine Finland, Helsinki Institute of Life Sciences, University of Helsinki, Helsinki, Finland; 5 Department of Mathematics and Statistics, University of Helsinki, Helsinki, Finland; 6 Department of Public Health, University of Helsinki, Helsinki, Finland; 7 TAUCHI Research Center, Faculty of Information Technology and Communication Sciences (ITC), Tampere University, Tampere, Finland; 8 Finnish Institute for Health and Welfare (THL), Helsinki, Finland; 9 Center for Economic and Social Research, University of Southern California, Los Angeles, CA USA; 10 Department of Economics, University of Southern California, Los Angeles, CA USA; 11 Institute of Genomics, University of Tartu, Tartu, Estonia

**Keywords:** Gestational diabetes, Genome-wide association studies, Type 2 diabetes

## Abstract

Gestational diabetes mellitus (GDM) is a common metabolic disorder affecting more than 16 million pregnancies annually worldwide^1,2^. GDM is related to an increased lifetime risk of type 2 diabetes (T2D)^1–3^, with over a third of women developing T2D within 15 years of their GDM diagnosis. The diseases are hypothesized to share a genetic predisposition^1–7^, but few studies have sought to uncover the genetic underpinnings of GDM. Most studies have evaluated the impact of T2D loci only^8–10^, and the three prior genome-wide association studies of GDM^11–13^ have identified only five loci, limiting the power to assess to what extent variants or biological pathways are specific to GDM. We conducted the largest genome-wide association study of GDM to date in 12,332 cases and 131,109 parous female controls in the FinnGen study and identified 13 GDM-associated loci, including nine new loci. Genetic features distinct from T2D were identified both at the locus and genomic scale. Our results suggest that the genetics of GDM risk falls into the following two distinct categories: one part conventional T2D polygenic risk and one part predominantly influencing mechanisms disrupted in pregnancy. Loci with GDM-predominant effects map to genes related to islet cells, central glucose homeostasis, steroidogenesis and placental expression.

## Abstract (web summary)

Genome-wide association analyses identify 13 loci associated with gestational diabetes, showing partial overlap with type 2 diabetes risk loci but also distinct genetic architecture predominantly influencing pregnancy-related mechanisms.

## Main

Gestational diabetes mellitus (GDM) is a common disorder of pregnancy that has substantially increased in prevalence across diverse population groups in the last 15 years^14^. Despite conferring substantial morbidity to both mother and child, relatively little is known about the genetics of GDM outside of a proposed shared genetic etiology with type 2 diabetes (T2D). The largest existing genome-wide association study (GWAS) of GDM revealed five genome-wide significant loci, all but one previously associated with T2D^13^. Although the results seem to broadly support the hypothesis of shared etiology, none of the existing GWAS were sufficiently powered to fully assess the degree to which genetic risk is shared between GDM and T2D. The one prior GDM locus not associated with T2D, while intriguing, is insufficient to identify mechanisms or biological pathways specific to, or with differential effects in, GDM.

To elucidate the genetic underpinnings of GDM, we conducted a GWAS^15^ of GDM in 12,332 cases and 131,109 parous female controls. Participants were of Finnish ancestry from the FinnGen study^16^. Cases were identified using Finnish health and population registry sources, including registry data from inpatient hospitalizations, outpatient specialty clinics and birth registry. Cases were confirmed to have a diagnosis within a pregnancy window, and those with diagnoses of diabetes before the index pregnancy were excluded (Methods; Supplementary Note).

**Fig. 1:**
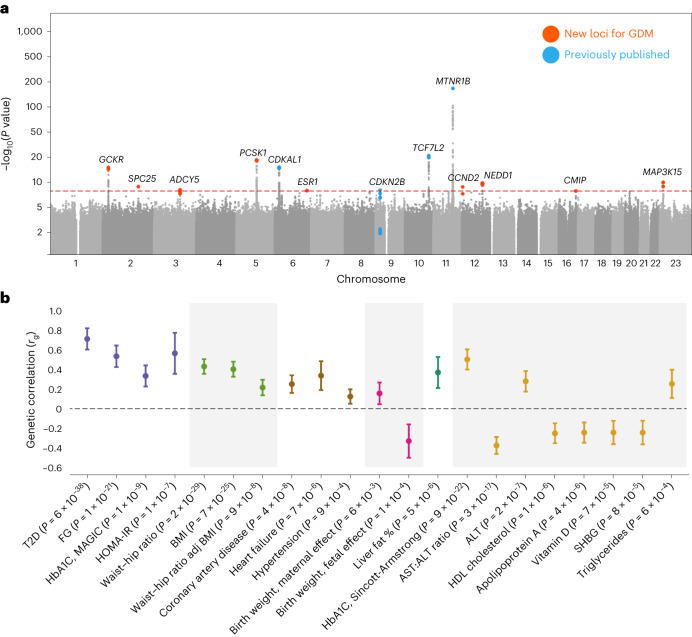
Genome-wide association results for GDM. **a**, Manhattan plot of GWAS of GDM in 12,332 cases and 131,109 parous female controls of Finnish ancestry with REGENIE 2.2.4. The *x* axis reflects chromosomal positions, and the *y* axis reflects −log_10_(*P*) values for the two-tailed association test for each variant, presented on a log scale. Red dotted line indicates the significance threshold (*P* = 5 × 10^−8^). Colored SNPs represent the credible set members for the 13 genome-wide significant loci, with blue indicating loci previously associated with GDM and orange indicating new associations. Labels indicate the gene nearest to the fine-mapped lead SNP. **b**, The genetic correlation (SNP-*r*_g_) between GDM and 53 other diseases, traits or biomarkers was computed using LD score regression. We plot the SNP-*r*_g_ with confidence intervals for all traits that were significant after Bonferroni correction for two-sided tests of 53 traits (*P* < 9.4 × 10^−4^). Results for all tested traits are reported in Supplementary Tables 19 and 20. Colors indicate phenotype category.

Our GWAS nearly tripled the previously known loci for GDM, identifying 13 distinct associated chromosomal regions (Fig. 1 and Supplementary Figs. 1–13). Significant variants include 4 of 5 previously reported GWAS loci. We observe a modest effect at the fifth, the *HKDC1* locus (rs9663238; *β* = 0.05, *P* = 0.0024), proposed as a unique GDM contributor, and note that, intriguingly, the only genome-wide significant finding for this variant in FinnGen is for intrahepatic cholestasis of pregnancy (FinnGen R11: *β* = 0.24, *P* = 1.6 × 10^−14^; Supplementary Table 2 and Supplementary Note).

To confirm the robustness of these findings, we performed replication studies using samples newly recruited to FinnGen after the data freeze and a large sample from the Estonian Biobank (EstBB; a combined 8,931 cases and 170,809 controls; Supplementary Table 3). Eleven of 13 associations replicated (the well-established T2D and previously observed GenDIP hit^13^ at *CDKN2B* was not significant but was directionally consistent as was the association at *CMIP*). Notably, the two new Finnish-enriched findings at *ESR1* and *MAP3K15* were both strongly confirmed (replication *P* values of 3.5 × 10^−5^ and 4.3 × 10^−6^, respectively).

Fine-mapping^17^ of the 13 loci pinpointed 14 independent signals (the region near *CDKN2B* containing two independent signals), of which nine regions had a 95% credible set containing five or fewer SNPs (Table 1 and Supplementary Table 1; Methods). Nine regions represented new GDM associations not reported in previous GDM GWAS. We characterized the 13 current confirmed GDM GWAS loci through annotation and colocalization of credible sets with >3,800 GWAS (Supplementary Tables 4 and 5), quantitative trait loci (QTLs) for gene expression, biomarkers and metabolites (Supplementary Tables 6–11) and chromatin interactions (Supplementary Table 11), along with tests of enrichment by functional consequence, gene expression or canonical gene sets (Supplementary Tables 13–16 and Supplementary Figs. 14–17; Methods). Given the consistency of the replication, we include also top results and fine-mapping of a joint GWAS of the FinnGen discovery and holdout samples (18,474 cases and 171,349 controls), which nominates additional new loci for further investigation (Supplementary Table 17).

**Table 1:** Fourteen genome-wide significant fine-mapped signals for GDM

Regions	Lead variants	Ref/Alt	AF	*β* (s.e.)	*P* values	Nearest genes	Annotation^a^	Cred. set size^b^	*P*_Class G_^c^	Known^d^
Class G loci (GDM-predominant effect)										
2:27508073-27519736	rs780093	T/C	0.649	0.12 (0.0149)	6.75 × 10^−16^	*GCKR*	Intron	3	0.995	T2D, FG
2:16890084	rs1402837	C/T	0.17	0.11 (0.0186)	3.87 × 10^−9^	*SPC25*, *G6PC2*	Intron	1	0.981	FG
5:96357306-96392261	rs1820176	T/C	0.314	−0.14 (0.0154)	7.86 × 10^−20^	*PCSK1*	Intron	26	>0.999	FG
6:151805650	rs537224022	C/G	0.00984	−0.447 (0.0812)	3.82 × 10^−8^	*ESR1*	5′ UTR	1	0.999	–
11:92975544	rs10830963	C/G	0.358	0.403 (0.0143)	8.65 × 10^−175^	*MTNR1B*	Intron	1	>0.999	GDM, T2D, FG
12:97449565-97470365	rs74628648	C/T	0.0786	−0.169 (0.027)	4.03 × 10^−10^	*NEDD1*	Intron	15	0.978	T2D, FG
16:81435701-81519035	rs2926003	C/T	0.337	−0.0824 (0.0151)	4.52 × 10^−8^	*CMIP*	Intron	48	0.987	–
X:19266251-19485409	rs56381411	C/T	0.0153	−0.404 (0.0638)	2.44 × 10^−10^	*MAP3K15*	Missense	4	>0.999	–
Class T loci (T2D-predominant effect)										
6:20673649-20703721	rs34499031	T/TAA	0.332	0.12 (0.0148)	5.10 × 10^−16^	*CDKAL1*	Intron	8	<0.001	GDM, T2D, FG
10:112994312-113014674	rs34872471	T/C	0.203	0.168 (0.0173)	1.69 × 10^−22^	*TCF7L2*	Intron	4	<0.001	GDM, T2D, FG
12:4275678-4367206	rs76895963	T/G	0.0305	−0.26 (0.0445)	4.69 × 10^−9^	*CCND2*	Intron	2	<0.001	T2D
Unclassified loci										
3:123346931-123405666	rs6798189	G/A	0.184	−0.103 (0.0186)	2.60 × 10^−8^	*ADCY5*	Intron	16	0.138	T2D, FG
9:22129580-22136490	rs1333051	A/T	0.115	−0.126 (0.0228)	2.92 × 10^−8^	*CDKN2B*	Regulatory	5	0.465	GDM, T2D, FG
9:22133646-22134652	rs7019437	C/G	0.438	0.0394 (0.0142)	5.49 × 10^−3^	*CDKN2B*	Intergenic	5	N/a	–

For each independent association identified by fine-mapping with SuSiE, the lead variant (highest posterior inclusion probability) and the region spanned by the credible set are reported. Loci are grouped according to their classification in the shared variants analysis (Methods; Supplementary Table 27). Genomic positions are on GRCh38. Reference (ref) and alternative (alt) alleles, alternative allele frequency (AF), GWAS results and nearest coding gene are given for the lead variant. *β* (log odds ratio), its standard error (s.e.) and corresponding unadjusted two-sided *P* values are from logistic regression using REGENIE.

^a^Most severe annotated consequence among variants in the credible set.

^b^Number of variants in the credible set for the region.

^c^Posterior probability that the lead variant is in the GDM-predominant class identified in the shared variants analysis. The secondary fine-mapped association on chromosome 9 (rs7019437) was omitted from that analysis.

^d^Whether the locus has been reported as significantly associated with GDM, T2D or FG in the previous GWAS^13,18,34^.

We next performed analyses to evaluate the shared genetic etiology with T2D. Assessment of genome-wide significant signals using our algorithm Significant Cross-trait Outliers and Trends in Joint York regression (SCOUTJOY; Methods) indicated that the 13 GDM-associated loci showed significant heterogeneity in their relationship to T2D (*P* < 0.001; Supplementary Table 18). Five of the 13 GDM-associated loci were not significantly associated (*P* < 5 × 10^−8^) with T2D in either a previously published large T2D meta-analysis^18^ or in FinnGen, while the remaining loci are established T2D hits (Table 1 and Supplementary Fig. 18). At the genomic level, GDM and T2D were genetically correlated (*r*_g_ = 0.71, s.e. = 0.06, *P* = 6.8 × 10^−37^), which is significantly greater than zero (*P* = 6.8 × 10^−37^) but less than 1 (*P* = 1.2 × 10^−7^; Methods). Significant genetic correlations were also seen with 12 diseases or traits and eight blood laboratory values in cases where the disorder or value was phenotypically related to GDM (Fig. 1, Supplementary Figs. 19 and 20 and Supplementary Tables 19–23). In both the genomic correlation and top hits comparison, GDM was significantly associated with fasting glucose (FG), hemoglobin A1c (HbA1C) and 2-h glucose results on oral glucose tolerance testing but was not associated with fasting insulin level. None of these glycemic traits or related disorders, however, appeared to stratify the 13 GDM-associated loci into distinct groups similar to T2D (Supplementary Fig. 21 and Supplementary Table 24). Comparison of the effect of GDM- and T2D-associated loci across sex and across pregnancy history indicated that the relationship was not generally mediated by pregnancy effects or sex differences (Supplementary Figs. 22 and 23 and Supplementary Tables 18, 25 and 26).

We then explored the relationship between GDM and T2D effects in more detail applying a Bayesian classification algorithm^19^ to the top associations for GDM and top associations for T2D selected to have comparable statistical evidence for association (13 loci for GDM and 15 loci for T2D; Methods; Supplementary Note). Initial assessment was performed using T2D effect sizes from a GWAS of male FinnGen participants (27,607 cases and 118,687 controls) to prevent the Bayesian algorithm from being affected by sample overlap. We then performed the same analysis in men and in women from a large external meta-analysis of T2D for comparison.

**Fig. 2:**
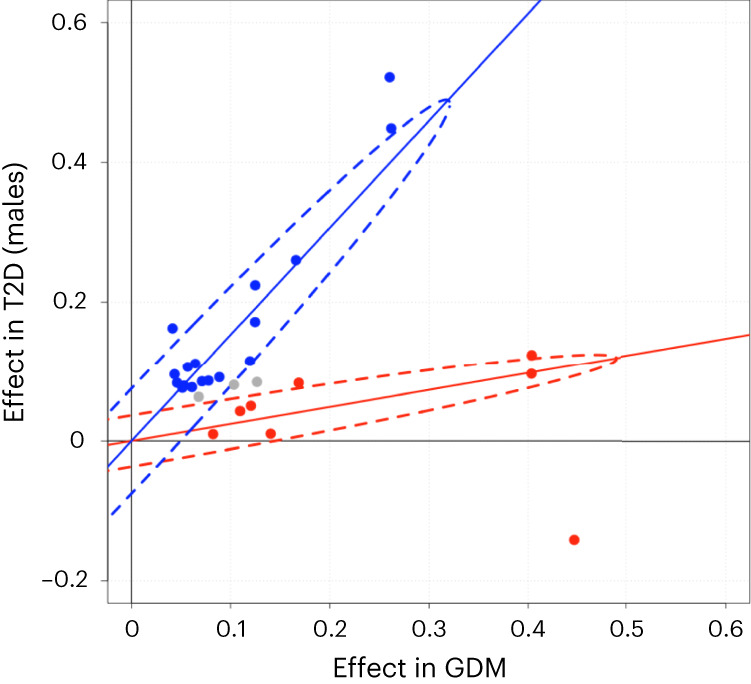
Classification of the genetic effects of SNPs in GDM and T2D. Comparison of log odds ratios in GWAS of GDM (*x* axis) and T2D in males (*y* axis) for top-associated SNPs from GDM (13 SNPs) and T2D (15 SNPs). The following two distinct classes of SNP effects were identified by a Bayesian classifier in shared variants analysis: class T (blue) containing SNPs with T2D-predominant genetic effects and class G (red) with GDM-predominant effects (Supplementary Table 27). Gray SNPs were not confidently assigned to either class (posterior probability >95%). Dotted ellipses indicate the 95% probability regions of the fitted bivariate effect size distributions with each class.

The shared variants analysis suggested that the genetics of GDM risk falls into two categories, one shared with T2D risk and the other predominantly gestational (Fig. 2 and Supplementary Table 27). Specifically, the comparison of effect sizes between GDM and T2D does not support the existence of a single, consistent relationship between GDM and T2D across loci, but instead proposes two distinct classes of significant variants in this scan (Fig. 2)—class G, with GDM-predominant effects, and class T, with T2D-predominant effects. The two-class model of relationship between GDM and T2D fits the observed distribution of odds ratios (ORs) significantly better than a single-class model (log_10_(Bayes factor (BF)) = 29.41). Class G contains 8 of the 13 GDM-associated loci that have GDM-predominant SNP effects, with effect sizes roughly three times greater in GDM than in T2D on average (Fig. 2 and Table 1). The majority of class G loci had a positive effect in T2D, but it was proportionately less than their effect in GDM. In comparison, the GDM-associated SNPs contained in class T had effects in the two disorders that were consistent with T2D-signals significantly associated with diabetes only in the T2D GWAS—namely, a reduced effect size in GDM versus T2D—a pattern of effects that was observed for all SNPs in class T. Variant classes were maintained whether comparing to T2D in men or women, with no evidence of a sex-specific classification (Supplementary Figs. 24 and 25 and Supplementary Tables 18 and 28). Stratification patterns were also consistent in T2D regardless of pregnancy history (Supplementary Fig. 25 and Supplementary Table 18) or inclusion of extended GDM results from GWAS including the FinnGen holdout set (Supplementary Figs. 26 and 27 and Supplementary Tables 29 and 30). Fasting plasma glucose associations occur in all classes, specifically with 5 of 8 class G loci, 2 of 3 class T loci and 2 of 3 unclassified loci.

The existence of the GDM-predominant class of effects, class G, distinct from those traditionally seen in T2D, raises the possibility of physiologic mechanisms of glycemic control with different actions or regulations during pregnancy (Supplementary Note, Supplementary Table 31 and Supplementary Fig. 28). As presented in Table 1, the eight class G loci have a peak SNP that is either intronic to a protein-coding gene, a missense mutation or, a 5′-UTR variant. Although the effects of a locus do not always operate through the nearest gene, several of the loci implicate genes involved in plausible cellular processes, for example, signal transduction and hormone processing. Examples of such genes^20–27^ are presented in Box 1.

**Fig. 3:**
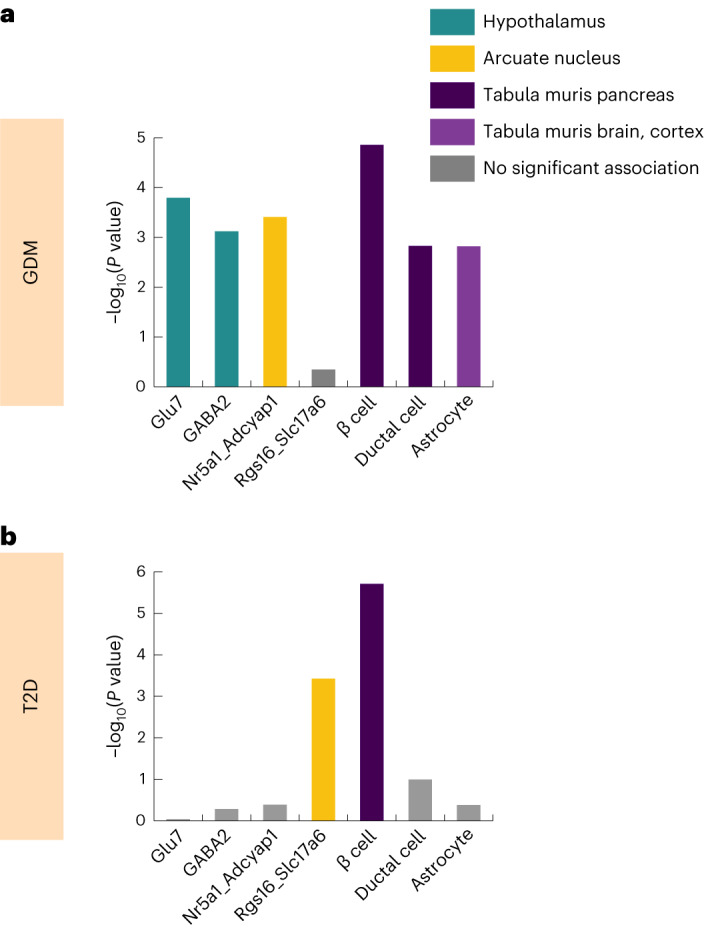
Cell-type specificity analysis of GDM and T2D highlights different cell associations. Cell-type specificity analysis was performed for GDM (**a**) and for prior meta-analysis of T2D (**b**) from ref. ^18^ using high-quality mouse single-cell RNA-seq datasets with FUMA v1.3.4 (Supplementary Tables 32–35). Unadjusted *P* values are reported for the two-sided association test between relative gene expression in the given cell type and multimarker analysis of genomic annotation (MAGMA) gene-level associations in the GWAS. Results are shown for cell types that both are significantly associated with at least one GWAS after correction for multiple testing of all cell types in all datasets (**a**) and have putatively independent association conditional on other cell types in the same RNA-seq dataset (**b**). Colors indicate the RNA-seq dataset source and significance.

Finally, to gain further insight into potential functional differences between GDM and T2D, we examined the cell-type specific expression patterns associated with the GWAS summary statistics^28^ (Methods; Fig. 3, Supplementary Tables 32–35 and Supplementary Figs. 30–32). We evaluate cell-type specific enrichment despite the lack of significant tissue-level enrichment because pregnancy induces major adaptive changes to specific cell populations within maternal tissues that might not be reflected in bulk tissue expression. Analyses integrating multiple large single-cell RNA expression datasets indicated that pancreatic β cells are significantly associated both with GDM and T2D. However, only GDM had significant associations with the hypothalamus, that is, hypothalamic GABAergic neurons (GABA2), hypothalamic glutaminergic neurons (GLU7) and neurons in the ventromedial hypothalamus (VMH) arcuate nucleus (NR5a1_Adcyap1; Fig. 3 and Supplementary Table 34).

Taken together, we present data from the largest GDM GWAS to date, identifying 14 independent signals in 13 associated chromosomal regions. This study replicates all five of the loci previously associated with GDM, albeit with indications of a weaker effect of *HKDC1* than previously reported, and discovers nine new loci. Our key finding is that GDM has a partially distinct genetic etiology, that is, while GDM and T2D in part share a polygenic predisposition, there is a second category of GDM genetic risk factors that are predominantly gestational contributors to disease. This contextualizes the substantial effect of the *MTNR1B* locus, which had been reported previously as an outlier^9^, but our data now show that *MTNR1B* is representative of a whole group of GDM-predominant loci, characterized by a larger effect on GDM than on T2D.

Further studies will be required to characterize the precise GDM-predominant molecular effects, but our current results suggest plausible mechanisms related to maternal adaptive physiological responses to pregnancy. Broadly, pregnancy increases circulating gestational hormones (for example, human placental lactogen, progesterone and estrogen) altering normal homeostatic glycemic pathways in the brain and pancreas as well as impaired insulin sensitivity in maternal peripheral tissues. The brain and pancreas both show clear enrichment of signal in our cell-type specificity analysis of GDM, with our results in the brain showing specific associations with hypothalamic and arcuate (ARC) neurons in GDM that are not seen in T2D (Fig. 3 and Supplementary Tables 32–34). The hypothalamus and ARC are connected by multiple neural pathways^29^, and both regions have been implicated in adaptive glycemic response during pregnancy^30^. In that context, our *ESR1* locus is particularly interesting given that the VMH contains glucose-sensing neurons that express the estrogen receptor-α (ERα, encoded by *ESR1*) and act to regulate glucose levels^31^. Moreover, in mice, ERα knockout or perturbation of estrogen levels (which occurs in pregnancy) alters the expression of multiple class G genes (for example, *PCSK1*, *MTNR1B* and *SPC25*-*G6PC2*) in ARC neurons that arise in the VMH^32^. Our cell-type specificity results particularly highlight Nr5a1_Adcyap1 in ARC, which projects from the VMH^33^ (Supplementary Fig. 31, Supplementary Table 34 and Supplementary Note). Given the complexity of GDM, however, much larger studies will be required to reach a comprehensive view of the molecular underpinnings of GDM susceptibility.

The current study design in the rather homogeneous Finnish population carries specific strengths and weaknesses associated with this analysis approach. On one hand, GWAS discovery is enhanced by population homogeneity^16^, and the linkage of national birth, inpatient and outpatient medical registries enables robust phenotyping (Methods). The generalizability of the results may suffer, however, as some detected loci may be for rare alleles specifically enriched in the Finnish population. In our analyses of GDM, two loci mapped to rare alleles enriched in Finland, which may be difficult to replicate elsewhere, while 70% of the loci correspond to variants that are common (minor allele frequency (MAF) > 10%), in non-Finnish European ancestry individuals (Table 1). Nonetheless, additional studies prioritizing ancestrally diverse populations are needed for a better understanding of the genetic underpinnings of GDM in all populations at risk.

In summary, we discovered nine new loci associated with GDM and demonstrated that GDM genetic risk is distinct from T2D both at the locus and genomic scale. Our results suggest that the genetics of GDM risk falls into the following two categories: one part T2D risk and one part predominantly gestational contributors to disease. Tissue characterization of GDM genetics further implicates tissues previously identified in adaptive pregnancy responses, raising hypotheses regarding genetic effects in these tissues during pregnancy. Broadly, this work underscores the benefits of focusing resources on pregnancy disorders as pregnancy is a natural perturbation that offers leverage to discover loci with new physiologic mechanisms of glycemic or homeostatic control.

Box 1 Background on selected candidate genes for class G loci, with GDM-predominant effects From the eight class G loci associated with GDM, we present background information on *MAP3K15, PCSK1* and *ESR1*, including a brief description of the gene product, function and previously established genetic associations.

**Table:** 

**Gene**	**Known genetic relationships and function**
*MAP3K15*	A missense variant (G838S, chrX_19380197_C_T_b38) protective against GDM was found in *MAP3K15*, which encodes a protein kinase that regulates apoptotic-mediated cell death and stress response. The gene has high expression in the adrenal glands and has previously been linked to steroidogenesis^20^ and polycystic ovarian syndrome^21^. The GDM-associated missense variant is rare outside of Finland, but rare loss-of-function variants in *MAP3K15* have recently been associated with T2D protection in UK Biobank^22^, where female carriers of such rare nonsynonymous variants had a 30% reduced risk of T2D and reduced blood glucose and HbA1C levels, and hemizygous male carriers of rare protein-truncating variants had a 35% reduced risk of T2D. Further characterization of the GDM-associated variant by phenome-wide association study (PheWAS) analyses in FinnGen indicated that the variant is associated with increased risk for hypertension (*β* = 0.11, *P* = 2.0 × 10^−8^), and we replicate a modest protective effect for T2D (*β* = −0.09, *P* = 1.8 × 10^−3^), which is considerably lower than that seen in GDM (*β* = −0.404).
*PCSK1*	*PCSK1*, which encodes prohormone convertase 1/3, critically regulates endocrine and neuronal prohormone processing. Previous data show that homozygous loss of function of PCSK1 results in a generalized and pleiotropic prohormone conversion defect characterized by severe obesity, impaired adrenal and thyroid function, reactive hypoglycemia, elevated levels of proinsulin and low levels of insulin^23^, whereas common gene variants have been associated with BMI^24^, fasting proinsulin, fasting glucose and T2D. Interestingly, the GDM-associated risk allele identified in our study is associated with lower BMI (*β* = −0.02, *P* = 5.3 × 10^−11^), lower weight (*β* = −0.02, *P* = 5.3 × 10^−11^) and lower height (*β* = −0.01, *P* = 3.9 × 10^−6^).
*ESR1*	One likely hormone-related class G signal is a roughly twofold Finnish-enriched low-frequency variant mapping to the 5′-UTR of the estrogen receptor gene, *ESR1*, which protects from GDM. The variant 6-151805650-C-G is several orders of magnitude more significant than neighboring variants and in FinnGen has >99% posterior probability of being the causal variant. Of note, in FinnGen, this variant is also associated with increased height (*β* = 0.07, *P* = 1 × 10^−16^) and similarly fine-mapped in a credible set of only one SNP. *ESR1* encodes ERα, which mediates both positive and negative feedback of estrogen on the hypothalamus, regulating puberty, ovulation and menopause. Homozygous loss-of-function results in elevated gonadotropins, delayed puberty, infertility, insulin resistance, increased adiposity and altered bone metabolism^25–27^.

## Methods

### Ethics statement

Participants in FinnGen provided informed consent for biobank research, based on the Finnish Biobank Act. Alternatively, separate research cohorts, collected before the Finnish Biobank Act came into effect (in September 2013) and the start of FinnGen (August 2017), were collected based on study-specific consents and later transferred to the Finnish biobanks after approval by Fimea, the National Supervisory Authority for Welfare and Health. Recruitment protocols followed the biobank protocols approved by Fimea. The Coordinating Ethics Committee of the Hospital District of Helsinki and Uusimaa (HUS) approved the FinnGen study protocol HUS/990/2017.

The FinnGen study is approved by Finnish Institute for Health and Welfare (permit: THL/2031/6.02.00/2017, THL/1101/5.05.00/2017, THL/341/6.02.00/2018, THL/2222/6.02.00/2018, THL/283/6.02.00/2019, THL/1721/5.05.00/2019 and THL/1524/5.05.00/2020), Digital and Population Data Service Agency (permit: VRK43431/2017-3, VRK/6909/2018-3 and VRK/4415/2019-3), the Social Insurance Institution (permit: KELA 58/522/2017, KELA 131/522/2018, KELA 70/522/2019, KELA 98/522/2019, KELA 134/522/2019, KELA 138/522/2019, KELA 2/522/2020 and KELA 16/522/2020), Findata (permit: THL/2364/14.02/2020, THL/4055/14.06.00/2020, THL/3433/14.06.00/2020, THL/4432/14.06/2020, THL/5189/14.06/2020, THL/5894/14.06.00/2020, THL/6619/14.06.00/2020, THL/209/14.06.00/2021, THL/688/14.06.00/2021, THL/1284/14.06.00/2021, THL/1965/14.06.00/2021, THL/5546/14.02.00/2020, THL/2658/14.06.00/2021, THL/4235/14.06.00/2021) and Statistics Finland (permit: TK-53-1041-17 and TK/143/07.03.00/2020 (earlier TK-53-90-20) TK/1735/07.03.00/2021).

The Biobank Access Decisions for FinnGen samples and data used in FinnGen Data Freeze 8 include the following: THL Biobank BB2017_55, BB2017_111, BB2018_19, BB_2018_34, BB_2018_67, BB2018_71, BB2019_7, BB2019_8, BB2019_26, BB2020_1, Finnish Red Cross Blood Service Biobank 7.12.2017, Helsinki Biobank HUS/359/2017, Auria Biobank AB17-5154 and amendment 1 (August 17, 2020), AB20-5926 and amendment 1 (April 23, 2020), Biobank Borealis of Northern Finland_2017_1013, Biobank of Eastern Finland 1186/2018 and amendment 22 § /2020, Finnish Clinical Biobank Tampere MH0004 and amendments (21.02.2020 and 06.10.2020), Central Finland Biobank 1-2017 and Terveystalo Biobank STB 2018001.

EstBB research was conducted in accordance with good ethical standards and was approved by the Estonian Committee of Bioethics and Human Research (1.1-12/1020).

### Cohort

The FinnGen Study is a public–private partnership project combining data from Finnish biobanks and electronic health records from national registries. The linked national health registers include data on hospital and outpatient visits, primary care, cause of death and medication records. Approval from the FinnGen Study was received to use the data in the present work. After a 1-year embargo, the FinnGen summary stats are available for download. In this study, we used the results from the FinnGen release R8, which includes data from 342,499 individuals and disease endpoints.

### Phenotyping

Full details of phenotyping are described in the Supplementary Note. Briefly, clinical endpoints with corresponding dates were constructed for gestational diabetes and related diagnoses for exclusions for all FinnGen participants as described in the Supplementary Note. Temporal phenotyping was then performed to phenotype each pregnancy for the presence of glycemic disease, and then individuals were assigned as cases or controls. Beginning with 330,000 pregnancies among genotyped FinnGen participants, we defined a ‘pregnancy window’ of 40 before delivery until 5 weeks after delivery. A pregnancy met inclusion criteria for ‘gestational diabetes’ if it had (I1) gestational diabetes International Classification of Diseases (ICD) codes occurring in the pregnancy window, (I2) any diabetes codes occurring in the pregnancy window (for example, for ICD8) or (I3) abnormal blood glucose test results in the pregnancy registry. Pregnancies were then excluded for the following: (E1) any previous diabetes diagnosis code occurring outside a pregnancy window; (E2) any previous significant pancreatic disease, including chronic pancreatitis, pancreatic necrosis, pancreatic cancer or cystic fibrosis or (E3) any previous type 1 diabetes (T1D) or T2D code. Pregnancies passing these exclusion criteria and without any inclusion criteria for gestational diabetes were designated as ‘unaffected.’ Then, to phenotype individuals, cases were identified among the 151,000 genotyped females with a history of pregnancy as those with at least one pregnancy meeting inclusion criteria for gestational diabetes and passing exclusion criteria. Controls were defined as females with only ‘unaffected’ pregnancies (that is, no diabetes or significant pancreatic diseases occurring before or during any pregnancy, and no abnormal blood glucose in the pregnancy registry).

### Genotyping and GWAS

A detailed description of the study design and analytical methods is available in the online documentation (https://github.com/FINNGEN/regenie-pipelines). In brief, FinnGen individuals have been genotyped with Illumina and Affymetrix chip arrays. Quality control was performed to remove samples and variants of poor quality. Imputation was performed using a population-specific SISu v3 imputation reference panel. A subset of unrelated individuals of genetically confirmed Finnish ancestry was identified. GWAS was performed using REGENIE 2.2.4. Sex, age, 10 principal components (PCs), and genotyping batch were included as covariates in the analysis.

### Fine-mapping

Fine-mapping of a 1.5-Mb locus around any GWAS lead SNP was performed using the SuSiE algorithm^17^, which reports causal variants and a 95% credible set for each independent signal (details described previously^16^ and at https://finngen.gitbook.io/documentation/methods/finemapping). As linkage disequilibrium (LD), we used in-sample dosages (that is, cases and controls used for each phenotype) computed with LDstore.

Independent signals were those that either represent the primary strongest signal with lead *P* < 5 × 10^−^^8^ or as secondary signals that must have genome-wide significance and log BF > 2.

### Replication

Replication was performed in (1) a replication holdout sample from FinnGen, (2) an EstBB cohort, (3) a meta-analysis of the FinnGen holdout and EstBB samples and (4) the previously published GenDIP consortium meta-analysis (Supplementary Note and Supplementary Table 3)^13^. For GenDIP, we also consider replication of the previously published loci in the current GWAS (Supplementary Table 2). The FinnGen replication cohort is a holdout sample of 6,026 cases and 45,296 controls with genotyping, phenotyping and analysis matching the current discovery GWAS.

The EstBB is a population-based biobank with 212,000 participants. The 198K data release was used for the replication analyses described here. All EstBB participants have signed a broad informed consent form. Participants with gestational diabetes were identified using the ICD-10 code system (information on ICD codes is obtained via linking with the National Health Insurance Fund and other databases). The EstBB replication cohort consists of 2,904 female cases with an ICD code for gestational diabetes (O24.4) and 125,513 female controls with genotyping, imputation and analysis performed as described previously^35,36^. All EstBB participants have been genotyped at the Core Genotyping Lab of the Institute of Genomics, University of Tartu, using Illumina Global Screening Array v1.0 and v2.0. Samples were genotyped, and PLINK format files were created using Illumina GenomeStudio v2.0.4. Individuals were excluded from the analysis if their call rate was <95% or if the sex defined based on heterozygosity of the X chromosome did not match the sex in phenotype data. Before imputation, variants were filtered by call rate <95%, Hardy–Weinberg equilibrium (HWE) *P* < 1 × 10^−4^ (autosomal variants only) and minor allele frequency <1%. Variant positions were updated to b37, and all variants were changed to be from TOP strand using GSAMD-24v1-0_20011747_A1-b37.strand.RefAlt.zip files from https://www.well.ox.ac.uk/~wrayner/strand/ webpage. Prephasing was done using Eagle v2.3 software38 (number of conditioning haplotypes Eagle2 uses when phasing each sample was set to:–Kpbwt=20000) and imputation was done using Beagle v.28Sep18.79339 with effective population size *n*_e_ = 20,000. Population-specific imputation reference of 2,297 WGS samples was used^37^.

Association analysis was carried out using SAIGE (v0.43.1) software implementing a mixed logistic regression model without LOCO option, using sex, age, age_sq and ten PCs as covariates in step I. Replication meta-analysis was performed using inverse-variance weighted fixed effects meta-analysis. Replication in each analysis was evaluated at *P* < 0.05 after Bonferroni correction for the number of loci available in the replication sample.

### Annotation

Variants were annotated with Ensembl Variant Effect Predictor version 104 (https://www.ensembl.org/info/docs/tools/vep/index.html) data to give the projected variant consequence. Each variant was also annotated for enrichment in Finland compared to compared to non-Finnish–Swedish–Estonian Europeans, as described previously^16^. Annotation with known prior GWAS loci was performed as previously described^16^. In brief, for each independent association, we annotated every phenotype in the GWAS Catalog that was significantly associated with either (1) the lead posterior inclusion probabilities (PIP) variant or (2) any variant in the credible set. Similar annotation was performed for metabolite associations from the MetSIM study^38^ (Supplementary Note). Each locus was also annotated with SNP2GENE in FUMA version v1.3.5d (fuma.ctglab.nl/snp2gene/) for chromatin interactions (Supplementary Table 12), expression QTL (eQTL) associations (Supplementary Table 6) and prior GWAS hits (Supplementary Table 5).

### Colocalization

Colocalization was performed on all fine-mapped regions as previously described for the FinnGen study^16^. In brief, the probabilistic model to integrate GWAS and eQTL data was eCAVIAR^39^, but the input PIPs were estimated by the SuSiE algorithm^17^. The eCAVIAR method uses PIPs for variants in each region to compute a colocalization posterior probability (Supplementary Note). The intersection of variants in credible sets was then checked across multiple phenotypes from FinnGen (Supplementary Table 4), GTEx (Supplementary Table 7), eQTL Catalog (Supplementary Table 8), GeneRisk (Supplementary Table 9) and the UK Biobank (Supplementary Table 10).

### Gene enrichment analysis

Gene-level association results from multimarker analysis of genomic annotation (MAGMA)^40^ were used to identify tissue and pathway enrichments using the SNP2GENE and GENE2FUNC modules of FUMA (version v1.3.4). The MAGMA results were tested for (1) association with gene expression levels in GTEx v8 (Supplementary Table 14 and Supplementary Fig. 14), (2) enrichment in sets of differentially expressed genes identified across tissues from GTEx v8 (Supplementary Table 15 and Supplementary Fig. 15), (3) enrichment in gene sets for pathways or other biological processes including those defined by KEGG (MsigDB c2), gene ontology (GO) biological processes (MsigDB c5) or WikiPathways (Supplementary Table 16) and (4) enrichment in gene sets defined by reported associations in GWAS Catalog (Supplementary Table 16 and Supplementary Fig. 17).

### Genetic correlation

We estimated the SNP heritability (ℎ^2^_SNP_) of GDM and pairwise genetic correlations (SNP-*r*_g_) between GDM and diabetes-related diseases and traits using LDSC version 1.0.1. Testing difference of ${r}_{g}$ from perfect correlation was performed using a one-tailed *z*-score test:

$$z=\frac{1-{r}_{\mathrm{g}}}{{\mathrm{s.e.}}\left({r}_{\mathrm{g}}\right)}$$

See Supplementary Note for details on additional genetic correlation analyses.

### SCOUTJOY

To compare the heterogeneity of GDM-associated loci’s genetic effects in any two disorders, we developed SCOUTJOY (Supplementary Note), substantively extending base methods introduced in MR-PRESSO^41^ that address heterogeneity detection while allowing both sample overlap and estimation error in both comparison GWASes rather than just one. The goal of SCOUTJOY is to estimate the primary relationship in effect sizes between the two disorders while accounting for estimation error. To accomplish this, we derive estimators for York regression^42^ with a fixed intercept. Global heterogeneity testing was then performed based on the overall goodness of fit of the York regression model to the observed distribution of effect size estimates. Outlier variants were identified as those where the goodness of fit is significantly improved by modeling the variant as having its own separate distribution. These goodness of fit tests provide an analytic solution replacing the null simulations used in MR-PRESSO. Code for SCOUTJOY and York regression with a fixed intercept is available on GitHub (https://github.com/aelliott08/SCOUTJOY).

### Shared variants analysis

We applied the linemodels package (https://github.com/mjpirinen/linemodels) to the GWAS summary statistics from T2D GWAS and GDM GWAS. The analysis included 28 lead variants from the GWAS analyses (13 from GDM and 15 from T2D). We classified the variants into two classes based on their bivariate effect sizes. The classes were represented by line models whose slopes were estimated using an EM algorithm, resulting in values of 1.53 (labeled as class T) and 0.25 (labeled as class G). For both models, the scale parameters determining the magnitude of effect sizes were set to 0.2 and the correlation parameters determining the allowed deviation from the lines were set to 0.99. The membership probabilities in the two classes were computed separately for each variant by assuming that the classes were equally probable a priori. Because the two GWAS did not have overlapping samples, the correlation of their effect estimators was set to 0.

### Cell-type specificity analyses

To get better resolution on specific cell types, we performed cell-type specificity analyses with high-quality single-cell mouse datasets using FUMA (https://fuma.ctglab.nl/tutorial#celltype; Supplementary Note). First, we identified tissue-level associations with Tabula Muris data^43^ identifying significant associations (false discovery rate (FDR) < 0.05) with expression in brain and pancreas after Benjamini–Hochberg multiple testing correction (Supplementary Fig. 30). We then performed cell-type specificity analyses as previously described^28^, augmenting Tabula Muris with additional high-quality scRNA-seq of hypothesized involved brain regions (Supplementary Note). Analysis was performed on genetic summary statistics for both our gestational diabetes GWAS and for a recent T2D European meta-analysis dataset^18^. We also compare the pancreatic results to the analysis of high-quality scRNA-seq of pancreas in humans to assess the impact of known differences in human versus mouse pancreatic cellular function and physiology (Supplementary Table 35 and Supplementary Fig. 32).

### Reporting summary

Further information on research design is available in the Nature Portfolio Reporting Summary linked to this article.

## Online content

Any methods, additional references, Nature Portfolio reporting summaries, source data, extended data, supplementary information, acknowledgements, peer review information; details of author contributions and competing interests; and statements of data and code availability are available at 10.1038/s41588-023-01607-4.

### Supplementary information

Supplementary Information Supplementary Note and Supplementary Figs. 1–32.

Reporting Summary

Supplementary Tables Supplementary Tables 1–35.

## Data Availability

The FinnGen data may be accessed through Finnish Biobanks’ FinnBB portal (www.finbb.fi) and THL Biobank data through THL Biobank (https://thl.fi/en/web/thl-biobank). The full summary statistics of the primary scan of GDM are available at https://www.ebi.ac.uk/gwas/studies/GCST90296696. GWAS of T2D in males, females, parous females and nulliparous females are available at https://www.ebi.ac.uk/gwas/studies/GCST90296697, https://www.ebi.ac.uk/gwas/studies/GCST90296698, https://www.ebi.ac.uk/gwas/studies/GCST90296699 and https://www.ebi.ac.uk/gwas/studies/GCST90296700. Additional data used for colocalization/annotations are available from GWAS Catalog (https://www.ebi.ac.uk/gwas/home), GTEx (https://gtexportal.org/home/datasets), EMBL-EPI eQTL Catalog (https://www.ebi.ac.uk/eqtl/), UK Biobank fine-mapping (https://www.finucanelab.org/data) and METSIM (https://pheweb.org/metsim-metab/). GeneRisk lipid QTL results will be available on GWAS Catalog (https://www.ebi.ac.uk/gwas/) upon publication of the corresponding manuscript (https://www.medrxiv.org/content/10.1101/2023.01.21.23284765v1.full). A complete list of sources used for annotation with FUMA, including download links, are available at https://fuma.ctglab.nl/links and https://fuma.ctglab.nl/tutorial#datasets; see Supplementary Note for details on datasets used in the current analysis. Previous GWAS results on T2D from ref. ^18^ are available from the DIAGRAM Consortium (https://diagram-consortium.org/downloads.html). GWAS results for glycemic traits are available from MAGIC (https://magicinvestigators.org/downloads/). Additional GWAS results used for genetic correlation analyses are also all publicly available—birth weight results from the EGG Consortium (https://egg-consortium.org/birth-weight-2019.html); BMI, height and WHR results from GIANT (https://portals.broadinstitute.org/collaboration/giant/index.php/GIANT_consortium_data_files); liver fat, NAFLD, T1D and number of children results on GWAS Catalog (https://www.ebi.ac.uk/gwas/; GCST90029073, GCST008468, GCST90014023 and GCST90029038, respectively); hypertension results on NHLBI GRASP (https://grasp.nhlbi.nih.gov/FullResults.aspx); coronary artery disease (CAD), heart failure and AtrialFib results from the Cardiovascular Disease (CVD) Knowledge Portal (https://cvd.hugeamp.org/downloads.html#summary) and Sinnott-Armstrong et al. biomaker results on FigShare (https://nih.figshare.com/articles/dataset/The_meta-analyzed_GWAS_summary_statistics_for_35_lab_biomarkers_described_in_Genetics_of_35_blood_and_urine_biomarkers_in_the_UK_Biobank_/12355382).

## Code availability

GWAS was performed using REGENIE 2.24 (https://rgcgithub.github.io/regenie/) with the pipeline described at the GitHub for FinnGen GWAS pipeline publicly described (https://github.com/FINNGEN/regenie-pipelines).

Fine-mapping of GWAS signals was performed using SuSIE 0.9.2 (https://stephenslab.github.io/susieR/index.html) with the FinnGen fine-mapping pipeline publicly described (https://github.com/FINNGEN/finemapping-pipeline).

Colocalization was performed using the FinnGen colocalization pipeline that is based on eCAVIAR (https://github.com/FINNGEN/pheweb-colocalization). Annotations were performed according to the FinnGen annotation pipeline (https://github.com/FINNGEN/autoreporting).

Genetic correlation was performed using LDSC v1.0.1 (https://github.com/bulik/ldsc).

Gene-level association results from MAGMA were used to identify tissues and pathways enrichment using the FUMA modules for SNP2GENE v1.3.5d, GENE2FUNC v1.3.4 and cell specificity module v1.3.4 (https://fuma.ctglab.nl/).

Comparison of heterogeneity of cross-disorder genetic effects was performed using SCOUTJOY v0.0.5 (https://github.com/aelliott08/SCOUTJOY). Classification of variants based on their bivariate effect size was carried out using Linemodels 0.2.0 (https://github.com/mjpirinen/linemodels).
