# Supplementary material for: Distinct and shared genetic architectures of gestational diabetes mellitus and type 2 diabetes
Source: Nat Genet. 2024 Jan 5;56(3):377–82. doi: 10.1038/s41588-023-01607-4 (PMC10937370; doi:10.1038/s41588-023-01607-4)
Supplement: Supplementary file 1 — Supplementary Note and Supplementary Figs. 1–32. [file 41588_2023_1607_MOESM1_ESM.pdf]

nature genetics

Letter

https://doi.org/10.1038/s41588-023-01607-4

Distinct and shared genetic architectures of gestational diabetes mellitus and type 2 diabetes

In the format provided by the authors and unedited

Table of Contents

Page #

Table of Contents *1*

SUPPLEMENTARY NOTE *3*

Supplementary Text *3*

I. Phenotyping **3**
- A. Available diagnosis data for phenotyping 3
- B. Mapping diagnostic codes to phenotype 3
- C. Relationship between diagnosis code and blood glucose levels 5

II. Replication of lead variants from prior GenDIP meta-analysis **6**
- A. Method for comparing genetic effects of a SNP between two studies 6
- B. Difference in estimated effect at HKDC1 6

III. Replication of the 13 loci from FinnGen GWAS **7**

IV. Colocalization comparison metrics **8**
- A. Causal Posterior Probability (CLPP) 8
- B. Causal Posterior Agreement (CLPA) 8
- C. Datasets 8

V. Finnish metabolite mQTL annotations **9**
- A. Dataset 9
- B. Method 9
- C. Results 9

VI. Comparison of genetic correlation results **10**
- A. Method to compare genetic correlation results 10
- B. Results 10

VII. Comparison of GDM with T2D and glycemic traits **11**
- A. Methods 11
  - 1. Derivation of York regression model with fixed intercept 11
  - 2. Estimation of correlation in sampling error for GWAS effect sizes 13
  - 3. Significant Cross-trait OUtliers and Trends in JOint York regression (SCOUTJOY) 13
    - i. Evaluation of general overall dispersion using the Global Test 14
    - ii. Identification of outliers 14
- B. Results 15
- C. Discussion 16

VIII. Genetic effect differences by sex or pregnancy history **17**
- A. Method for comparing genetic effects of a SNP in two traits 17
- B. Results 17

IX. Selection of variants for shared variants analysis **19**

X. Comparison of relationship of genetic effects in BMI and GDM for Class T and Class G loci **20**
- A. Method to generate and compare genetic effect relationships 20
- B. Results 20

XI. Cell type specificity analyses **21**
- A. Datasets for analysis 21
- B. Method for cell specificity analysis 22
  - 1. Identify cells with scRNA expression significantly associated with genetic summary statistics 22
  - 2. Identify independently associated cell types within each dataset 22
  - 3. Identify independent signals across cells in different datasets/tissues 22

C. Results.. 22

Supplementary Figures ***24***

Supplementary Figure 1: Regional GWAS results for rs780093 locus on chromosome 2 at 27519736.......................... 25
Supplementary Figure 2: Regional GWAS results for rs1402837 locus on chromosome 2 at 168900844...................... 25
Supplementary Figure 3: Regional GWAS results for rs6798189 locus on chromosome 3 at 123376465...................... 26
Supplementary Figure 4: Regional GWAS results for rs1820176 locus on chromosome 5 at 96360881........................ 26
Supplementary Figure 5: Regional GWAS results for rs34499031 locus on chromosome 6 at 20676183...................... 27
Supplementary Figure 6: Regional GWAS results for rs537224022 locus on chromosome 6 at 151805650.................. 27
Supplementary Figure 7: Regional GWAS results for rs1333051 locus on chromosome 9 at 22136490........................ 28
Supplementary Figure 8: Regional GWAS results for rs34872471 locus on chromosome 10 at 112994312.................. 28
Supplementary Figure 9: Regional GWAS results for rs10830963 locus on chromosome 11 at 92975544.................... 29
Supplementary Figure 10: Regional GWAS results for rs76895963 locus on chromosome 12 at 4275678.................... 29
Supplementary Figure 11: Regional GWAS results for rs74628648 locus on chromosome 12 at 97457224.................. 30
Supplementary Figure 12: Regional GWAS results for rs2926003 locus on chromosome 16 at 81488676.................... 30
Supplementary Figure 13: Regional GWAS results for rs56381411 locus on chromosome 23 at 19380197.................. 32
Supplementary Figure 14: Gene property analysis of tissue specific expression.. 33
Supplementary Figure 15: Tissue specificity analysis of enrichment of differentially expressed gene sets (DEG). 34
Supplementary Figure 16: Heat map of gene expression. 35
Supplementary Figure 17: Enrichment of annotated genes for finemapped GDM loci in GWAS Catalog gene sets...... 36
Supplementary Figure 18: Genetic effect comparison in GDM vs T2D for GDM-associated loci.................................... 37
Supplementary Figure 19: Genetic correlation of GDM with 22 Traits. 38
Supplementary Figure 20: Genetic correlation of GDM with 29 biomarkers.. 39
Supplementary Figure 21: Comparison of effects of GDM-associated loci in GDM vs. glycemic traits 40
Supplementary Figure 22: Comparison of effects of GDM-associated loci in T2D in males vs T2D in females 42
Supplementary Figure 23: Comparison of effects of GDM-associated loci in T2D in parous females vs T2D in nulliparous females 42
Supplementary Figure 24: Comparison of shared variants analysis of GDM-associated loci in GDM vs. T2D by sex 43
Supplementary Figure 25: Comparison of effects of GDM-associated loci in GDM vs. T2D subgroups by sex and pregnancy history 44
Supplementary Figure 26: Comparison of shared variants analysis of extended data (FinnGen Release 11) GDM-associated loci in GDM vs. T2D by sex.. 46
Supplementary Figure 27: Comparison of effects of GDM-associated loci from extended data (FinnGen Release 11) in GDM vs. T2D subgroups by sex 47
Supplementary Figure 28: Comparison of GDM vs BMI effects sizes by GDM variant class 48
Supplementary Figure 30: Cell type specificity analysis with GDM and T2D summary statistics.................................. 49
Supplementary Figure 31: Cross dataset conditional analyses reveal independent signals for GDM and for T2D......... 50
Supplementary Figure 32: Cell type specificity analysis with GDM summary statistics in mouse vs human islets......... 52

Supplemental References ***53***

SUPPLEMENTARY NOTE

Supplementary Text

I. Phenotyping

A. Available diagnosis data for phenotyping

Registry data is available for all FinnGen participants from national health registers. This includes data on hospital and outpatient visits (HILMO - Care Register for Health Care), Primary care visits (AvoHILMO - Register of Primary Health Care), the Medical Birth Register (including data on the mother's diseases during pregnancy: ICD-9,10), Causes of Death, reimbursed medication entitlements and prescribed medicine purchases, and the Finnish Cancer Registry. Clinical endpoints were constructed from the register codes using Finnish version of International Classification of Diseases, 10th revision (ICD-10) diagnosis codes and harmonizing those with definitions from ICD-8 and ICD-9.

B. Mapping diagnostic codes to phenotype

As stated previously, all phenotyping was performed using only ICD codes or registry data. Codes related to diagnoses are as follows:

Phenotype	Codes
Gestational diabetes	*ICD10*: O244;<br><br>*ICD9*: 6480A;<br><br>*ICD8*: None
Diabetes	*ICD10*: E10, E10.[0-9], E11, E11.[0-9], E13, E13.[0-9], E14, E14.[0-9], M14.2, H36.0[0-9], O24, O24.[1-4];<br><br>*ICD9*: 250, 250.[0-8]A, 250.[0-8]B, 250.[0-8]C, 250.[0-8]X, 3620A, 3620B, 6480A, 6488A;<br><br>*ICD8*: 250, 2500[0-9],76110;<br><br>KELA drug reimbursement registry: 103, 215;<br><br>Drug ATC code: A10AB0[1-6], A10AB30, A10AC0[1-4], A10AC30, A10AD0[1-6], A10AD30, A10AE0[1-6], A10AE30, A10AE54, A10AE56, A10BA, A10BA0[1-3], A10BB, A10BB0[1-9], A10BB1[0-2], A10BB31, A10BC, A10BC01;
Chronic pancreatitis and pancreatic necrosis	*ICD10*: K86.01, K86.08, K86.1;<br><br>*ICD9*: 5771A, 5771B, 5771C, 5771D;<br><br>*ICD8*: 57710, 57719, 57702,57793;
Pancreatic cancer	*ICD10*: C25, C25.[0-9];<br><br>*ICD9*: 157, 157[0-4]A, 157[8-9]X;<br><br>*ICD8*: 157, 15701, 15787, 15788, 15799, 23060;

Cystic fibrosis	*ICD10*: E84, E84.[0-9];<br><br>*ICD9*: 2770A;<br><br>*ICD8*: 27300;

Temporal phenotyping was then performed in 2 steps: (1) pregnancies with gestational diabetes were identified, (2) individual females were assigned as cases or controls.

Date of delivery/ the child's birth date of 330,000 children born to female FinnGen participants was identified from the Population Register or the Medical Birth Register. Then a "pregnancy window" was generated for each pregnancy from 40 weeks before delivery to 5 weeks after. The allotted timing was to account for variability introduced by pseudoanonymization of time scale within and across registries. A pregnancy was classified as having "gestational diabetes" or "unaffected" based on the following criteria:

	Inclusion Criteria	Exclusion Criteria
Pregnancy with GDM	Pregnancies were included if they had any of the following:<br><br>1. Abnormal blood glucose listed in the Medical Birth Registry<br>2. Gestational diabetes code† received in the pregnancy window<br>3. Diabetes code received in the pregnancy window	Pregnancies were excluded if any of the following occurred prior to the index pregnancy:<br><br>1. Significant pancreatic disease including:<br>• Chronic pancreatitis<br>• Pancreatic necrosis<br>• Pancreatic cancer<br>• Cystic fibrosis<br><br>2. Diabetes diagnosis code occurring outside a pregnancy window<br>3. Type 1 or Type 2 diabetes† at any point prior
Unaffected pregnancy	None of the GDM inclusion criteria	Same as above
†Recall these definitions contain only ICD9 and ICD10 codes.		

The inclusion criteria reflect that a gestational diabetes diagnosis code was only available in ICD9 and ICD10 whereas in ICD8 there was only a general "diabetes" code. To include representation from ICD8 era, we constructed the inclusion and exclusion criteria to pull females who received a diagnosis of diabetes during a pregnancy window who had no other diabetes codes prior to pregnancy. The vast majority of cases were from ICD9 and ICD10 with only 730 cases added. Comparison of pregnancy labels created with the addition of ICD8 to the cohort to those without demonstrated they were sufficiently similar to proceed.

Among the 151,000 genotyped females with a pregnancy, cases were identified as those with gestational diabetes in at least 1 pregnancy. Controls are females with a history of pregnancy where all pregnancies were "unaffected". In other words, controls have (1) no abnormal blood glucose in the

pregnancy registry, (2) no diabetes codes occurring prior to or during any pregnancy, and (3) no significant pancreatic disease prior to or during any pregnancy.

C. Relationship between diagnosis code and blood glucose levels

Phenotyping is based on clinical diagnosis. The criteria for the diagnosis have shifted over the study period globally, and the Finnish Care Guidelines have matched the recommendations of the International Association of Diabetes and Pregnancy Study Groups (IADPSG), World Health Organization (WHO), the European Association for the Study of Diabetes (EASD), or the American Diabetes Association (ADA).The currently used threshold for diagnosis of diabetes during pregnancy in Finland is fasting plasma glucose $\geq$ 5 mmol/l, 1-hour glucose $\geq$ 10 mmol/l or 2-hour glucose $\geq$ 8.6 mmol/l.

II. Replication of lead variants from prior GenDIP meta-analysis

Prior meta-analysis in the GenDIP consortium identified 5 loci associated with GDM (Supplementary Table 2). Four of the 5 loci achieved genome-wide significance in our GWAS. All 5 loci were significantly replicated in the FinnGen GWAS after Bonferroni correction (p < .05/5) and were beta sign concordant.

However, we should have also been well-powered to detect a variant with the OR previously observed for *HKDC1* given our 1.8 fold increased effective sample relative to GenDIP. Furthermore, it appeared that our estimated effect at the locus was different from that observed previously.

A. Method for comparing genetic effects of a SNP between two studies

To determine if any of the GenDIP GDM loci had effects significantly different from those observed in our GWAS, we compared the effect estimates at each locus between studies. We computed the Z-score for the difference in observed genetic effects in each of two studies for each locus in GenDIP, *i*:

$$Z_i = \frac{\beta_{1,i} - \beta_{2,i}}{\sqrt{se_{1,i}^2 + se_{2,i}^2}}$$

P-values for the comparison for each SNP are obtained from a two-sided test of this Z-score, and evaluated after Bonferroni correction for multiple testing of the 5 GDM loci. Results are given in Supplemental Table 2.

B. Difference in estimated effect at *HKDC1*

There was a significantly different effect size estimate for one locus, *HKDC1*, after correcting for the number of tests (p-value 0.009). This was the previously identified locus that was not identified in the FinnGen GDM GWAS. In FinnGen (R11), *HKDC1*, is a strong locus for Intrahepatic Cholestasis of Pregnancy (ICP; beta=0.24, p=1.6e-14). Differences in *HKDC1* locus genetic effect magnitude should be considered in the context of the differences in phenotyping between GenDIP and this study. In GenDIP, phenotyping in the largest European cohort included as cases (1) an ICD-10 E10 - E14 diagnosis in the hospital inpatient admission data, followed by an obstetrical diagnosis within 0–120 days or if it preceded an obstetrical diagnosis by 0–180 days, (2) O24.4 (Diabetes mellitus arising in pregnancy) or O24.9 (Diabetes mellitus in pregnancy, unspecified) diagnosis in the Hospital inpatient admissions data, (3) GDM self-report diagnosis (code 1221, field 20002 “Non-cancer illness code, self-reported”), and (4) GDM self-report diagnosis using field 4041 "Did you only have diabetes during pregnancy?". In contrast, the phenotyping in the current work excluded cases of diabetes in pregnancy resulting from pre-existing diabetes (type 1 or type 2) restricting cases to only O24.4 or registry noted gestational diagnosis; additionally, there was no mechanism for sampling pregnancy complications.

III. Replication of the 13 loci from FinnGen GWAS

In a discovery cohort of 12,332 cases and 131,109 controls, we identified 13 loci associated with GDM. Replication was then performed in multiple cohorts including (1) a replication holdout sample from FinnGen, (2) an Estonian Biobank cohort, and (3) a meta-analysis of the two. We did not use the previously published GenDIP meta-analysis[1] for replication because it contains overlapping Finnish cohorts and did not contain results for 2 of our loci that were low frequency Finnish-enriched alleles. While the GenDIP results were not used for replication we do provide these results where available in Supplementary Table 3 together with all replication results.

For replication, we had a reserved holdout sample from FinnGen with 6,026 cases and 45,296 controls. The holdout cohort underwent the same temporal phenotyping employed for the discovery cohort described in section II above. Of the 13 loci identified by the discovery cohort, 10 loci replicated in the holdout sample ($p < .05/13$). Three loci did not replicate: *CDKN2B*, *NEDD1*, and *CMIP*.

We also replicated these findings in a sample from the Estonian Biobank, which should have allele frequencies similar to those in the Finnish population[2]. This sample was composed of 2,904 cases of females with an ICD code for gestational diabetes and 125,513 female controls. Of 13 loci, 8 loci replicated in the holdout sample.

We then meta-analyzed the results from the Finnish holdout replication sample and the Estonian Biobank cohort for our 13 loci. We did not include the GenDIP samples in this combined replication meta-analysis because they overlapped both the FinnGen and the Estonian. Eleven loci replicated ($p < .05/13$) in the combined replication including 1 locus, *NEDD1*, that did not replicate in the FinnGen holdout GWAS alone.

In summary, of the 13 loci identified in our discovery cohort, 11 loci replicated (Supplementary Table 3). Of the two loci that did not replicate, *CDKN2B* and *CMIP,* both were sign concordant and one, *CDKN2B*, is a previously known T2D locus that was also significantly associated with GDM in the GenDIP consortia data.

IV. Colocalization comparison metrics

We perform colocalization on finemapped credible sets obtained from SuSiE using the posterior inclusion probabilities (PIP) estimated by SuSIE. For the colocalization we report both the Causal Posterior Probability (CLPP) and the Causal Posterior Agreement (CLPA). Colocalization was performed for the FinnGen release 8 endpoints (Supplementary Table 4), gene expression data from GTEx (Supplementary Table 7) and the eQTL Catalogue (Supplementary Table 8), lipid measures (Supplementary Table 9) and biomarkers (Supplementary Table 10).

A. Causal Posterior Probability (CLPP)

The Causal Posterior Probability (CLPP) is computed similarly to Hormozdiari et al (equation 8)[3]. For the credible set of phenotype 1 (cs_1) and 2 (cs_2).

$$CLPP = \sum_{i \in cs_1 \cap cs_2}^{i} x_i * y_i$$

where x and y are vectors containing the PIP for cs_1 and cs_2, respectively. Notably CLPP is dependent on the credible set size.

B. Causal Posterior Agreement (CLPA)

The Causal Posterior Agreement (CLPA) is independent of credible set size and defined by

$$CLPA = \sum_{i \in cs_1 \cap cs_2}^{i} \min\,(x_1 * y_i)$$

C. Datasets

Colocalization was performed for SuSiE fine-mapped data from: FinnGen phenotype endpoints from FinnGen release 8 (Supplementary Table 4); gene expression data from 49 tissues from donors of mixed ancestry in GTEx v8 (Supplementary Table 7)[4]. Gene expression data from EMBL-EBI eQTL Catalogue on 24 tissues/cell types[5] from samples of predominantly European ancestry (Supplementary Table 8); metabolism eQTL datasets from the GeneRISK study[6,7], including 186 lipid species (Supplementary Table 9); and 36 continuous endpoints and 57 biomarkers from UK Biobank(Supplementary Table 10).

V. Finnish metabolite mQTL annotations

We annotate the SuSiE finemapped credible sets for GDM and Type 2 diabetes with metabolite QTLs (mQTL) on circulating metabolite data from the Finnish METSIM study[8] .

A. Dataset

The Metabolic Syndrome in Men (METSIM) study[8] is a Finnish population study 10,197 Finnish males examined in 2005-2010. Genetic data includes both array data and exome data. Fasting blood metabolites were obtained with NMR mass spectrometry. Notably, this is an all male study which is a potential limitation for our GDM annotations however this is in the same population as our study. Every SNP in METSIM has been tested for association with 1391 metabolites, 937 after removing metabolites that are classified as xenobiotics or uncharacterized.

B. Method

We evaluated the 28 diabetes loci (the 13 GDM associated loci and 15 loci from the T2D GWAS used in the shared variants analysis) to annotate the SuSiE finemapped credible sets with mQTLs on circulating metabolite data from the METSIM study. To annotate the loci, we choose a representative lead SNP for the metabolite annotation (metLead variant) that is the credible set variant with the highest SuSiE posterior inclusion probability, PIP, that occurs in the METSIM study. If no credible set member appears in METSIM then we assign the metLead variant as the variant in METSIM with the highest LD to the lead variant if the r2 is > 0.2, otherwise assign NA. Associations with 937 blood metabolites were then extracted for each of the metLead SNPs. Metabolites with significant p-values after Bonferroni correction were then annotated to the locus (threshold 5.3e-5 for multiple testing of 937 metabolites).

C. Results

We provide mQTL annotations for all of the GDM loci and for T2D loci. Seven of the 13 GDM loci have mQTL annotations. Novel GDM loci are associated with glucose, mannose, branched chain amino acids, serine, androgens, bile acids, and lipids. Detailed annotations provided in Supplementary Table 11.

VI. Comparison of genetic correlation results

Gestational diabetes was tested for genetic correlation (r_g) with 24 disorders or traits and with 29 biomarkers. Significantly correlated traits or biomarkers had their r_g in GDM compared to the r_g with T2D to determine if there was a significantly different overlap with T2D than GDM.

A. Method to compare genetic correlation results

For both GDM and T2D, initial pairwise genetic correlations (SNP-r_g) between GDM and other traits was performed using LD Score Regression (LDSC) Version 1.0.1 with previously computed LD scores from European ancestry individuals from the 1000 Genomes Project. We then compared the magnitude of SNP-r_g that was seen for each trait with GDM vs T2D by computing the Z-score for the comparison in SNP-r_g between trait 1 and 2 as follows:

$$Z = \frac{r_{g,gdm} - r_{g,t2d}}{\sqrt[2]{se(r_{g,gdm})^2 + se(r_{g,t2d})^2}}$$

P-values for the comparison were computed from the Z-score.

B. Results

Significant genetic correlations were identified with 12 diseases or traits and 8 blood laboratory values all of which had been phenotypically related to GDM (Supplementary Table 19 & 20; Figure 1b). The 12 traits with significant genetic correlation included glycemic traits such as fasting glucose, Hb1C, HOMA-IR, and 2 hour glucose on OGTT testing but not Fasting insulin (Supplementary Table 19). Adulthood disorders in which we observed significant rg to GDM like Coronary Artery Disease (CAD), Heart Failure (HF), and Hypertension (HTN) have previously been phenotypically linked to gestational diabetes[9-11]. Significant biomarkers included liver and lipid biomarkers linked to GDM[12-14]as well as apolipoprotein A and Vitamin D that have recently been implicated prior to or early in GDM pregnancies[15-17].

We then took the eleven traits with significant genetic correlation (excluding T2D) and compared the r_g in GDM to the r_g in T2D, (Supplementary Table 21 and Supplementary Figure 19). Three traits had significantly different r_g with GDM than with T2D after multiple testing correction: Waist Hip Ratio, BMI, and CAD. Comparison of the r_g to GDM versus to T2D in 20 biomarkers showed a significant difference in the genetic correlation of two biomarkers after correction for multiple testing: urea and gamma glutamyltransferase (Supplementary Table 22 and Supplementary Figure 20).

In particular, maternal GDM is known to affect child birth weight. Genetic studies of birth weight have identified both fetal and maternal genetic associations with the child birth weight[18] and have used structural equation modelling to distinguish the separate contributions of maternal and fetal effects. Based on the observed genetic correlation of these maternal and fetal effects with fasting glucose it was hypothesized that genetic signals for GDM in the mother would be positively corelated with child birth weight while genetic signals for GDM in the fetus would be negatively correlated with fetal birth weight. Intuitively this is because glucose but not insulin passes the placenta and fetal insulin is an important growth hormone stimulating fetal size and development. Thus, a fetal genetic effect that results in reduced GDM risk was thought to also correspond to reduced insulin levels and reduced production of this hormone essential for growth. Our GDM GWAS confirms this hypothesis that GDM has a significant positive genetic association with maternal GWAS of birthweight and maternal effect on birth weight but a negative genetic correlation with fetal GWAS of birth weight and fetal effect on birth weight (Supplementary Table 19,21 and 23). However contrary to the hypothesis in the paper, we do not see any significant association to fasting insulin levels at current sample sizes, suggesting any genetic correlation of GDM with general fasting insulin levels is likely modest (Supplementary Table 19&24.

VII. Comparison of GDM with T2D and glycemic traits

Initial evaluation of the top SNPs for GDM comparing their genetic effects in GDM and T2D suggested a relationship as in Supplementary Figure 20. To more formally compare the genetic effect of 13 GDM-associated loci on GDM versus T2D and 6 other disorders, we extended existing methods[19-22] to determine trends of effects across the loci and identify outliers that have different pattern of effects than the majority of SNPs. Our approach, Significant Cross-trait OUtliers and Trends in JOint York regression (SCOUTJOY), has the advantage of: (1) accounting for sampling error in the GWAS effect estimates for each phenotype, (2) allowing sample overlap or other sources of correlated sampling error, and (3) making no assumptions about a causal relationship between the phenotypes. To accomplish this we first had to derive estimating equations for a special case of York regression that has not been addressed by existing literature. Using this tool we were able to compare the loci's genetic effects in GDM vs glycemic traits including T2D and the glycemic traits such as fasting glucose, 2h glucose on Oral Glucose Tolerance Testing (OGTT), and fasting insulin.

Variables	
X_i, Y_i	GWAS effect sizes for SNP *i* for the two diseases/traits being compared
i	Index for chosen top SNPs 1,…,*K*
K	Total number of SNPs being compared
$\sigma_{x,i}$, $\sigma_{y,i}$	Standard errors of X_i and Y_i for SNP *i*
ρ_i	correlation of the errors for x_i and y_i
a	Intercept of regression line comparing effect sizes between two traits
a_0	Chosen fixed intercept for regression
b	Slope of the regression line comparing effect sizes between two traits
W_i	Weights $$\frac{1}{\sigma_{y,i}^2 + b^2\sigma_{x,i}^2 - 2b\rho_i\sigma_{x,i}\sigma_{y,i}}$$
$\widehat{X_i}, \widehat{Y_i}$	Estimate of the effect size under the York regression slope
$\alpha_{h2,x}$	estimated intercept from univariate LD score regression for the GWAS of the first phenotype
$\alpha_{h2,y}$	estimated intercept for GWAS of the second phenotype
α_{rg}	estimated intercept from bivariate LD score regression for the genetic correlation between the two phenotypes

A. Methods

1. Derivation of York regression model with fixed intercept

We want to compare the trend in genetic effects between the two disorders and identify outliers. To accomplish this we need a regression tool that allows for (1) sampling errors in GWAS scans for both disease X and Y, and (2) sample overlap, or other sources of correlated sampling errors in the two diseases. York regression is designed for this task, finding a best fit line for two variables that are observed with known measurement error (co)variance. Briefly, for observed variables X and Y, York regression finds the intercept, a, and slope, b, such that

$$Y_i = a + bX_i$$

[1]

minimizes the sum of squared weighted residuals

$$S = \sum_{i=1}^{K} \left[\frac{(Y_i - bX_i - a)^2}{\sigma_{y,i}^2 + b^2\sigma_{x,i}^2 - 2b\rho_i\sigma_{x,i}\,\sigma_{y,i}}\right]$$

[2]

where $\sigma_{x,i}^2$, $\sigma_{y,i}^2$, and ρ_i are the squared standard errors and estimated correlation of sampling errors for the two GWAS effect size estimates for each SNP *i*. Compared to standard linear regression, York regression avoids attenuation and inefficient error estimates that would be caused by the ignoring

uncertainty in the predictor variable (*X*). This can be thought of as a generalization of Deming regression, which addresses the case where X and Y have error that is independent ($\rho_i = 0$).

For our purposes (see SCOUTJOY below) we want to estimate York regression with the intercept fixed to 0. To our knowledge, no estimator has previously been derived for the fixed intercept case for York regression. Using the loss function S (Equation 2) treating the intercept as fixed to a_0 we can then take the derivative with respect to the slope, *b*, to get the gradient for optimization

$$\theta(b;X,Y) = \sum_i W_i^2\left[b\left(\sigma_{y,i}^2 x_i^2 + b\sigma_{x,i}^2 x_i v_i - b\rho_i\sigma_{x,i}\sigma_{y,i}x_i^2\right) - \left(\sigma_{y,i}^2 x_i v_i + b\sigma_{x,i}^2 v_i^2 - \rho_i\sigma_{x,i}\sigma_{y,i}v_i^2\right)\right] = 0$$

[3]

where and are the standard York regression weights for residual variation

$$W_i = \frac{1}{\sigma_{y,i}^2 + b^2\sigma_{x,i}^2 - 2b\rho_i\sigma_{x,i}\sigma_{y,i}}$$

[4]

It follows that the best fit slope can be estimated by:

$$b = \frac{\sum_i W_i^2 v_i\left(\sigma_{y,i}^2 x_i + b\sigma_{x,i}^2 v_i - \rho_i\sigma_{x,i}\sigma_{y,i}v_i\right)}{\sum_i W_i^2 x_i\left(\sigma_{y,i}^2 x_i + b\sigma_{x,i}^2 v_i - b\rho_i\sigma_{x,i}\sigma_{y,i}x_i\right)}$$

[5]

As in regular York regression, this estimator for the slope is not a closed form solution since depends on the slope, *b,* both directly and through the weights. Instead, the slope and weights can be calculated iteratively. The initial slope estimate is made based on a grid search of possible values around ordinary and weighted least squares regression fits with the fixed intercept. *W* and *b* are then updated iteratively until *b* converges.

As with previous York papers[19,21], the error variance of the slope can be estimated using the propagation of errors under the implicit function theorem.

$$\sigma_b^2 = \frac{\sum_{i=1}^{k}\left(\frac{\partial\theta}{\partial x_i}\right)^2 \sigma_{x,i}^2 + \left(\frac{\partial\theta}{\partial y_i}\right)^2 \sigma_{y,i}^2 + 2\left(\frac{\partial\theta}{\partial x_i}\right)\left(\frac{\partial\theta}{\partial y_i}\right)\sigma_{xy,i}}{\left(\frac{\partial\theta}{\partial b}\right)^2}$$

[6]

Which in the case of a fixed intercept simplifies to:

$$\sigma_b^2 = \frac{\sum_i W_i^2\left[\sigma_{y,i}^2 x_i^2 + \sigma_{x,i}^2 v_i^2 - 2\rho_i\sigma_{x,i}\sigma_{y,i}x_i v_i\right]}{D^2}$$

[7]

Where the denominator term D is:

$$D = \frac{1}{b}\sum_i W_i x_i v_i + 4\sum_i W_i[(\beta_i - x_i)\beta_i] + \frac{1}{b}\sum_i W_i[(bx_i - v_i)\beta_i] - \frac{1}{b}\sum_i W_i^2\left[\left(\rho_i\sigma_{x,i}\sigma_{y,i}\right)(bx_i - v_i)^2\right]$$

[8]

The work above allows us to perform York regression with a fixed intercept. For our application it is also necessary to estimate predicted values for the observed data points based on the fitted regression (sometimes referred to as least squared adjusted points). It can be shown that prior estimators of these predicted values for York regression[19,23] are equivalent[20] and that they still hold with a fixed intercept, which when simplify to

$$\widehat{X_\iota} = W_i\left[X_i\left(\sigma_{y,i}^2 - b\rho_i\sigma_{x,i}\sigma_{y,i}\right) + (\mathrm{Y} - a_0)\left(b\sigma_{x,i}^2 - \rho_i\sigma_{x,i}\sigma_{y,i}\right)\right]$$

[9]

$$\widehat{Y_\iota} = b\widehat{X_\iota} + a_0$$

[10]

With this we can construct an approach to compare genetic effects in two disorders as per below.

2. Estimation of correlation in sampling error for GWAS effect sizes

York regression assumes that for each observation the sampling errors in X_i and Y_i ($\sigma_{x,i}^2$ and $\sigma_{y,i}^2$, respectively) and their correlation (ρ_i) are known. For our application to GWAS loci, standard error estimates for the effect sizes are normally available, but the sampling covariance is not normally estimated. To avoid requiring a joint model to be fit for each locus, we estimate the correlation ρ_i from GWAS summary statistics.

Specifically, it has previously been observed that the covariance of sampling errors in normalized GWAS effect sizes can be estimated, up to scaling factors for sample size, based on the intercept terms of univariate and bivariate LD score regression[24].Thus if $\alpha_{h2,x}$ is the estimated intercept from univariate LD score regression for the GWAS of the first phenotype, $\alpha_{h2,y}$ is the corresponding intercept for GWAS of the second phenotype, and α_{rg} is the estimated intercept from bivariate LD score regression for the genetic correlation between the two phenotypes, then we estimate

$$\rho = \frac{\alpha_{rg}}{\sqrt{\alpha_{h2,x}\alpha_{h2,y}}}$$

[11]

as the sampling correlation of the GWAS effect sizes for all SNPs in the analysis. We assume this correlation is likely to be stable across SNPs under the assumption that any sampling covariance is likely to be driven by sample overlap and phenotypic correlation and that these values will be constant across SNPs in our current analysis. We therefore use this estimate of ρ for all loci in defining weights for York regression.

3. Significant Cross-trait OUtliers and Trends in JOint York regression (SCOUTJOY)

We are interested in the question of whether there is a consistent relationship in the effect sizes between GDM and T2D or other traits, as might be expected for instance if the loci for GDM reflect exposing the same underlying genetic risk factors as T2D. Genetic correlation estimates can help quantify this relationship on the genome-wide level, but we are interested in also making this comparison among genome-wide significant loci. Our interest is not only in quantifying the average relationship, but in identifying whether the observed locus results are consistent with a uniform relationship between the two phenotypes or if certain loci can be identified as outliers, potentially indicating differential roles or relative importance in each context.

This question is statistically similar to the types of heterogeneity MR-PRESSO[22] was developed to detect in mendelian randomization. Although we explicitly do not intend to draw any causal inferences about the pair of phenotypes, these diagnostic tests match our interest in identifying trends and outliers among the top genome-wide significant loci for a pair of traits.

Therefore we here adapt the tests introduced by MR-PRESSO[22] to our use case, to (a) use York regression to account for measurement error in the effect size estimates from both GWAS, including possible correlated error due to e.g. sample overlap, and (b) extend outlier detection in cases where initial estimates of the trend in correlating effect size estimates may be unstable. We denote this

adapted approach as Significant Cross-trait OUtliers and Trends in JOint York regression (SCOUTJOY).

To begin, we identify the lead SNPs for the genome-wide significant loci in the first phenotype (e.g. the 13 top SNPs for GDM) and get their estimated GWAS effect sizes and corresponding standard errors in GWAS of each trait (e.g. GDM and T2D), with effect sizes oriented to the risk increasing allele in the first phenotype. We then use York regression with the intercept fixed to zero (as described above) to estimate the slope representing the average trend in the relationship of the effect sizes for the two phenotypes for the selected SNPs. Fixing the intercept at zero represents a null model that the selected loci based on GWAS of the first phenotype aren't expected to have an average effect on the second phenotype across SNPs that is unrelated to the first phenotype. Adopting this null model facilitates closely following the approach of MR-PRESSO to evaluating excess heterogeneity across SNPs relative to this null trend.

After fitting this initial trend line by York regression, we then evaluate both (1) general overdispersion relative to a single trend line and (2) whether individual SNPs can be identified as outliers relative to the effects seen for other SNPs in the group. Outliers are of particular interest, so adaptations were made to extend the current approach of MR-PRESSO.

i. <u>Evaluation of general overall dispersion using the Global Test</u>

We first evaluate whether the distribution of observed residuals from each point to the fitted regression line are larger than would be expected if the true SNP effect sizes followed a single line. MR-PRESSO[22] previously performed this Global Test using simulated null replicates, but we provide an analytic solution using the standard goodness of fit test for the York regression model. Specifically, after fitting the fixed-intercept York regression to all points as described above to estimate the regression slope $\hat{b}$, we compute the observed loss from the weighted residuals summed over all K SNPs

$$S = \sum_{i=1}^{K} W_i \left(Y_i - \hat{b} X_i\right)^2 \quad [12]$$

with weights

$$W_i = \frac{1}{\sigma_{y,i}^2 + \hat{b}^2 \sigma_{x,i}^2 - 2\hat{b}\rho_i \sigma_{x,i}\sigma_{y,i}} \quad [13]$$

The value of *S* is then tested as a chi-squared statistic with K-1 degrees of freedom. A significant value of S indicates that the observed effect sizes are not consistent with the true effect sizes for the two traits being directly proportional across all SNPs.

ii. <u>Identification of outliers</u>

Beyond overall overdispersion, we would like to identify if individual points are outliers relative to the bivariate trend line. Using the concept of goodness of fit from the Global Test we can identify if any outliers are present. This test, however, focuses on whether the data is better fit by treating a given variant as an outlier modelled separately from the slope fit to the remaining variants ($\hat{b}_{-i}$). Since there may be multiple outliers present, we use an iterative approach to avoid the outlier status of a given variant depending on a regression that is potentially biased by other outlier points. This way we estimate a stable set of significant outliers, which we expect to be scientifically informative about the structure of the genetic relationship between the pair of phenotypes.

The first step in outlier identification is to test the outlier status of each individual SNP, *i*. To do this we perform a likelihood ratio test comparing the likelihood of SNP *i* being part of the same regression

model as the other *j* SNPs versus having a different mean. Since SNP *i* is perfectly fit by its own mean, the fit of the outlier model is determined by the fit of fixed-intercept York regression the other SNPs *j*, leaving out SNP *i.* Using the leave-one-out slope estimate $\hat{b}_{-i}$ the resulting loss function is

$$S_{-i} = \sum_{j \neq \mathrm{i}} \frac{\left(Y_j - \hat{b}_{-i} X_j\right)^2}{\sigma_{y,j}^2 + \hat{b}_{-i}^{\ 2} \sigma_{x,j}^2 - 2\hat{b}_{-i} \rho_j \sigma_{x,j}\, \sigma_{y,j}}$$

[14]

The likelihood ratio test of outlier status for SNP i is then given by comparing this value to the fit of the York regression model fit including SNP i (Equation 12).

$$S_i = S - S_{-i}$$

[15]

S_i is tested as 1 degree of freedom chi-squared statistic corresponding to the null hypothesis that SNP *I* is appropriate fit by the same regression model as the other SNPs. We evaluate the significance of this test with Bonferroni correction for the K SNPs tested to identify candidate outliers

Using this same structure we can then perform iterative outlier detection against a regression fit that excludes previously identified outliers and repeating this until the chosen set of outliers is stable. This includes evaluating whether previously identified outliers are no longer outliers after updates to the regression fit. Specifically, full the iterative outlier detection process works as follows:

1. For each of the K total SNPs, test for outlier status by performing a likelihood ratio test of whether SNP *i* is part of same regression model with all other SNPs based on leave-one-out York regression as described above (Equation 15). Denote the set of variants that are significant after Bonferroni correction for multiple testing as O_1. If there are no identified outliers then the procedure stops here.
2. For each of the K points, perform a new leave-*n*-out York regression excluding both the *i*th variant and the set of putative outliers O_1. Based on those regression results identify new outliers by performing the likelihood ratio test of whether SNP *i* is part of the same regression model as the current non-outlier SNPs (i.e. omitting O_1) versus having its own separate mean. From these new tests, determine which of the K points are significant outliers after Bonferroni correction for K variants, denoting this new set as O_2. Note that this means that an outlier detected in a previous iteration could be returned to "non-outlier" status if it no longer appears to be an outlier after other exclusions.
3. If the putative outlier sets O_1 and $O_2$are identical then the outlier detection has converged. If O_2does not match O_1 but is identical to an outlier set identified in some previous iteration then the procedure has become stuck in a loop and so iteration is halted without convergence. Otherwise, O_1 is updated to match O_2 and step 2 is repeated.

If the process converges then the variants in the final stable set O_1 are concluded to be significant outliers relative to the primary trend of relationship in GWAS effect sizes between the two phenotypes. While convergence is not guaranteed, in practice we do not encounter issues with infinite loops in the current analysis.

B. Results

For the 13 GDM-associated loci, we compared the genetic effects in GDM with 5 other disorders including Type 2 diabetes, fasting glucose, HbA1C, 2h Glucose on Oral Glucose Tolerance Testing (OGTT) and fasting insulin (Supplementary Figure 21; Supplementary Table 18&24). The comparison to T2D showed a group of outliers with strong T2D effect indicating a stratification that was not seen in any of the other glycemic traits, (Supplementary Table 18, Supplementary Figure 21). Falling into more than one group suggests some variants might have a different effect in pregnancy than the majority of

variants that have an effect in both GDM and T2D. This would be further explored in the shared variants analysis(Figure 2, Supplementary Table 27 & 28). After removal of the outliers, there was a significant positive relationship between genetic effects in GDM and in T2D for the 13 loci (Supplementary Table 18).

While the other glycemic traits did not have a stratified result seen in T2D, the relationship between genetic effects in GDM and in other traits (the trend line slope) was still informative (Supplementary Figure 21, Supplementary Table 24). A significant positive relationship was seen in fasting glucose (slope 0.19, p-value =5.2e-12) , HbA1C (slope 0.07, p-value =2.35e-10), and 2h glucose on OGTT(slope 0.15, p-value =4.0e-6) which suggests a uniform expected positive effect in SNPS in both disorders. In fasting insulin, no significant relationship was seen (slope= -1.24E-03, p-value=0.79). Complete list of slopes in Supplementary Table 24

C. Discussion

We were able to compare genetic effects across related diseases using our new method, SCOUTJOY, that leverages a newly derived regression form. This detected a unique relationship between GDM and T2D that was not present with other traits and could detail the relationship or lack thereof with glycemic traits.

VIII. Genetic effect differences by sex or pregnancy history

GDM is only experienced by pregnant females, so any observed differences between genetic effects for GDM and T2D could be confounded with differences in T2D genetic effects by sex or pregnancy history. We therefore evaluated whether the observed relationship of genetic effects between GDM and T2D was consistent across T2D in different groups by sex or pregnancy history. To evaluate the influence of sex or pregnancy status on the relationship between GDM-associated loci and T2D, we compared the effects of these loci in T2D across sex and across pregnancy history.

We first performed a genome-wide association for T2D in (a) T2D in males, (b) T2D in females, (c) T2D in parous females who had a history of pregnancy and (d) T2D in nulliparous females with no history of pregnancy. Within each group, cases were those with T2D and controls were those without T2D.

The stratified T2D GWAS allow comparison of sex or pregnancy effects in several ways. First, we test each of the 13 top hits from GDM for differences in T2D effect sizes between males and females or between parous and nulliparous females (method below). Second, we assess whether the overall trend in effect sizes for GDM and T2D across the 13 GDM hits differs when comparing to T2D in males vs. females or parous vs. nulliparous females using SCOUTJOY. Third, we consider the genome-wide genetic correlation of GDM with T2D in each sex and pregnancy history groups, as well as genetic correlation of T2D GWAS between the different groups.

A. Method for comparing genetic effects of a SNP in two traits

To determine if any of the GDM hits showed differences in T2D effects by sex or pregnancy history we compared the effect of each locus between groups. Following the same method previously used for testing sex differences in GWAS effect sizes[25], for each comparison (i.e. male vs. female, parous vs. nonparous) we computed the Z-score for the difference in observed genetic effects in each of two nonoverlapping groups for each top GDM SNP *i*:

$$Z_i = \frac{\beta_{1,i} - \beta_{2,i}}{\sqrt{se_{1,i}^2 + se_{2,i}^2}}$$

P-values for the comparison for each SNP are obtained from a two-sided test of this Z-score, and evaluated after Bonferroni correction for multiple testing of the 13 GDM loci.

B. Results

For the 13 GDM-associated loci, we compared the genetic effect on T2D in males versus females. Direct comparison of genetic effects at these loci revealed a significant difference at the *CKDN2B* locus (Supplementary Table 26) We then evaluated the relationship of genetic effects in T2D in males versus females using SCOUTJOY, (Supplementary Figure 22, Supplementary Table 25). The magnitude of genetic effect were very similar (slope=1.00, se=0.06) between sexes after identification of the one outlier at the *CDKN2B* locus. Effect plots of GDM versus T2D in each sex generally concordant, (Supplementary Figure 26) with the broadly similar slopes for the relationship of GDM with T2D in males (slope=0.44, se=0.05) and with T2D in females (slope=0.31, se=0.03).

Comparison of the genetic effects in T2D was then performed for females with a history of pregnancy (parous females) and those without (nulliparous females) (Suppl. Direct comparison revealed that none of the 13 GDM-associated loci were significantly different in these groups (Supplementary Table 26). Evaluation of the relationship of genetic effects using SCOUTJOY (Supplementary Figure 23, Supplementary Table 18) similarly revealed no outliers or heterogeneity (global test p=0.41) and generally concordant effect sizes (slope=0.77, se=0.11), though with higher uncertainty in nulliparous females due to lower sample size.

We further assessed genome-wide genetic correlation of GDM with T2D in males and females with different pregnancy histories, respectively (Supplementary Table 25). Highly similar genetic correlations were observed for GDM with males (r_g =0.76, se = 0.062), females (r_g =0.78, se =0.067), and parous females (r_g =0.77, se =0.074). While the correlation of GDM vs nulliparous females (r_g=0.94, se=0.16) appears different from the GDM vs the other subgroups, there's no sign that the genetics of T2D in parous females and nulliparous females actually differs (r_g=1.18, se=0.16). The initial observation could be attributable to sampling variation given the wider standard error and smaller sample size of the GWAS in nulliparous females. We do however find evidence that T2D in males is not perfectly genetically correlated with T2D in parous females (Z-score 1.73, p = 0.01) despite the high genetic correlation (r_g 0.92), potentially consistent with the observation of a single outlier with sex differences at the locus level for *CDKN2B*.

In conclusion, findings were highly concordant across sex with only one locus with significantly higher effect in women (Supplementary Figure 22, Supplementary Table 18,25-26). High concordance was also between women with and without a history of pregnancy (Supplementary Figure 23, Supplementary Table 18,25-26). Taken together this suggests that the relationship of GDM loci with T2D is not generally mediated by pregnancy effects or sex differences in the etiology of T2D (e.g. including any direct effect of GDM on T2D), and instead the observed structure is consistent across groups.

IX. Selection of variants for shared variants analysis

As stated in the Online Methods, we used the linemodels package (https://github.com/mjpirinen/linemodels) to compare summary statistics for 28 lead variant from both the T2D and GDM GWAS (13 from GDM and 15 from T2D).

Our goal was a fair comparison of effect sizes between our significant GDM loci and previously known T2D loci we might expect to have similar power to detect under a null hypothesis that the effect size in GDM is the same as in T2D. T2D has a higher effective sample size in FinnGen, because it is both more common and is not restricted to parous females. Thus to balance the comparison, we took only the T2D loci that would have been identified in a smaller study equivalent to the power of the GDM GWAS.

To do this, we first identified the minimum standardized effect size β^*_{min} among the genome-wide significant GDM loci (i.e. standardized for allele frequency).

$$\beta^*_j = \beta_j \sqrt{(2p_j(1-p_j)}$$

$$\beta^*_{min} = \min_j |\beta^*_j|$$

where β_j is the estimated log odds ratio for variant *j* in GDM GWAS, p_j is the minor allele frequency for variant *j* and β^*_j is the standardized effect size for variant *j*. It can be shown that the expected GWAS χ^2 statistic for a variant with observed standardized effect size β^* can then be approximated by

$$\chi^2_j \approx NK(1-K){\beta^*_j}^2$$

where *N* is the GWAS sample size and *K* is the proportion of cases in the GWAS sample. It may be noted that $NK(1-K)$ is proportional to conventional estimates of effective sample size. We therefore estimate that a variant with the minimum detected standardized beta from the GDM GWAS β^*_{min} would correspond to an expected GWAS χ^2 of

$$\chi^2_{j,T2D} = N_{T2D}K_{T2D}(1-K_{T2D}){\beta^*_{min}}^2$$

in the FinnGen GWAS of T2D with sample size N_{T2D} and in-sample prevalence K_{T2D}.

Based on the sample size for T2D in FinnGen release 6 (37031 cases, 214308 controls) we find the resulting $\chi^2_{j,T2D}$ corresponds to a p-value of 1.27e-22. Using this threshold we identified 15 independent loci from the T2D GWAS passing this p-value threshold that we would therefore expect to have power to detect in the GDM GWAS if the GDM and T2D effect sizes were equal. We therefore use the lead variants for this set of 15 T2D loci along with the 13 significant GDM loci for our comparison of top hit effect sizes.

X. Comparison of relationship of genetic effects in BMI and GDM for Class T and Class G loci

After identifying Class T and Class G SNPs, we were interested in their potential relationship with BMI. We focus here on BMI because we are not well powered to evaluate apparent patterns in other glycemic traits due to the smaller sample size of their published GWAS. For each class we compared the effects of member loci in gestational diabetes vs BMI using SCOUTJOY (Supplementary Figure 28 Supplementary Table 31). For each of the classes of SNPs we calculate the relationship between SNP effect in GDM and SNP effect in BMI IRN. We then compare the relationships observed across classes.

A. Method to generate and compare genetic effect relationships

Summary statistics from the GWAS for GDM, T2D, and inverse rank normalized BMI (BMI IRN) were used from FinnGen release 8. We then compared the effects using SCOUTJOY algorithm as detailed above. Comparison of slopes from these independent sets of variants was performed using Z-score.

$$Z = \frac{slope_{Class_T} - slope_{Class_G}}{\sqrt[2]{se(slope)^2_{Class_T} + se(slope)^2_{Class_G}}}$$

B. Results

The Class G loci, which have a GDM-predominant effect, had no significant relationship between the genetic effect in GDM and in BMI IRN across all variants (slope=0.015, p=0.039; Supplementary Figure 28a, Supplementary Table 31), and only a weak relationship after exclusion of 1 outlier (slope=0.024, p=6.4e-03). In contrast, the Class T loci, which have predominant effect in T2D, had a significant relationship between the genetic effect in GDM and BMI after outlier removal (beta -0.12, p 2.7-07). The relationship observed between GDM and BMI IRN was significantly different between Class G and Class T loci when the slopes were compared after excluding outliers (Z-score 9.6, p=6.0e-22). The negative relationship seen between top hits in Class T and BMI IRN echoes observations made previously about a negative correlation between genetic effects of top T2D hits and BMI[26]. We observe the same pattern in SCOUTJOY comparison of genetic effect in BMI IRN with T2D for class T loci (Supplementary Figure 28c; slope -0.08, p-value =5.09-08).

XI. Cell type specificity analyses

We were interested assessing the relationship between the GDM summary statistics and single cell expression across the body – particularly in comparison to the T2D summary statistics. To that end, we assessed the relationship between genetic summary statistics and single cell RNA (scRNA) datasets to detect relationships with specific cells and tissues. We focused on independent signals from each tissue and then evaluated cross-tissue signal dependence related to each disease. We performed this analysis on cell-specific tissue expression despite the lack of significant findings on bulk level tissue expression (Supplementary Figure 15 and 16) because we know that adaptive physiologic changes in pregnancy induces major adaptive changes to specific cell populations within maternal tissues and effects on expression specific to these cell populations might be obscured in bulk tissue expression.

A. Datasets for analysis

Genetic datasets tested include (1) our GDM summary statistics and, for reference, for (2) summary statistics from a recent Type 2 Diabetes European meta analysis dataset[27].

The scRNA datasets were tested in a staged manner, summarized below. We first performed this analysis on a large, well curated murine scRNA dataset that provides survey level data across 22 different organ/tissue types. Based on the results of this analysis, significant tissues were then more carefully evaluated with tissue specific murine datasets from hypothesized brain regions of interest. In the tissues/organs indicated, we also ran analyses on high quality human scRNA datasets where possible and provide comparisons.

Name	Description	PMID column
Tabula Muris FACS	Mouse samples were tested resulting in 119 cell-tissue pairs from 22 tissues/organs. scRNA expression was measured in 53,760 cells in the raw read count matrix, after QC 44,949 cells exist in the annotation file. Cells with label "unknown" were included as it was stated in the original study that they are potential novel cell types. Number of genes tested was 23,433 genes with 15,131 genes were mapped to human ENSG ID.	30283141 (ref.[28])
Mouse Hypothalamus (GSE87544)	Mouse hypothalamus samples were obtained from 8-10 week old mice and tested resulting in 46 cell types from hypothalamic tissue. scRNA expression was measured in 14,437 cells, after QC the total number of cells used was 1,039. Number of genes tested was 23,284 genes with 15,116 genes were mapped to human ENSG ID.	28355573 (ref.[29])
Mouse arcuate ME neurons (GSE93374)	Mouse brain samples were obtained from the hypothalamic arcuate-median eminence complex in 4-12 week old mice. Testing resulted in 28 cell types from the arcuate nucleus tissue. scRNA expression was measured in 21,086 cells resulting in 13,079 cells after QC and clustering. Number of genes tested was 19,743 genes of which 14,366 genes were mapped to human ENSG ID.	28166221 (ref.[30])
GSE84133	Mouse: All 1,886 cells were and from 14,878 genes, 12,741 genes were mapped to unique human ENSG ID.	27667365 (ref[31])
GSE84133	Human pancreas samples from individuals without diabetes. 7,266 cell were sampled. Number of genes tested was 20,125 genes and 19,546 genes were mapped to unique ENSG ID.	27667365 (ref[31])

For complete dataset details see summarized descriptions (https://fuma.ctglab.nl/tutorial#celltype). Multiple testing correction was performed using the Benjamini-Hochberg multiple test correction (FDR).

B. Method for cell specificity analysis

Assessing relationships across multiple scRNA-seq datasets is made particularly challenging due to complex batch and sampling differences between studies. A method addressing these concerns has been described by Wantanabe et al[32]. This method for a cell specificity analysis has been made available as part for the FUMA package. We briefly review the process below but for details please refer to the paper and online tutorials (https://fuma.ctglab.nl/tutorial#celltype). The analysis is a three-step process.

1. Identify cells with scRNA expression significantly associated with genetic summary statistics

For each cell/tissue type in each dataset, gene-level GWAS summary statistics from MAGMA are regressed on gene expression levels for the cell/tissue type and technical covariates.

2. Identify independently associated cell types within each dataset

For each dataset, all pairs of cell/tissue types that were significantly associated with the GWAS MAGMA results in Step 1 are systematically tested for conditional association. For each cell type pair, a proportional significance (PS) is then calculated:

$$PS_{\tau,\varphi} = \frac{-\log_{10}(p_{\tau,\varphi})}{-\log_{10}(p_{\tau})}$$

Where is the marginal p-value for a cell type and is the conditional p-value for cell type τ conditioning on cell type φ. Using the PS score for each cell type, the signal between cell type is then determined to be dependent or independent.

3. Identify independent signals across cells in different datasets/tissues

All cell/tissue types showing within-dataset conditional association in Step 2 are compared across datasets to identify cell/tissue types that have substantial independent association (PS > 0.5) with the GWAS MAGMA results.

C. Results

First we performed the initial step of cell specificity analysis on the summary statistics for GDM on single cell RNA expression data from the Tabula Muris dataset which gives a high-level survey of expression data across 22 tissues in a well-characterized murine sample (Supplementary Figure 30, Supplementary Table 32). Differences were seen between GDM and T2D in this initial screen with GDM having (1) significant associations with cells in the brain not seen in T2D, and (2) different pancreatic cell types being associated with GDM than T2D. Because the cell-level brain data in Tabula Muris was limited, we then repeated the step 1 analysis augmenting the Tabula Muris dataset with data from several high quality scRNA studies of the murine brain. From this analysis, differences in independent signals in cell-tissue data between GDM and T2D were seen (Figure 3, Supplementary Table 33) that reveal GDM was associated with two cell populations in the hypothalamus and one in the arcuate nucleus that were distinct from the one cell-type association in the arcuate nucleus seen in T2D. This is of particular interest as it is well established that adaptive changes in pregnancy occur in the ventromedial hypothalamus (VMH) and arcuate nucleus to regulate blood sugar and blood pressure during pregnancy[33].

To consider the relationship signal in the cells of the brain had with cells in the pancreas, we performed cross dataset conditional analyses on these data (Supplementary Figure 31, Supplementary Table 34) which revealed that one of the GDM hypothalamic cell associations in Glu7 appeared related to the arcuate nucleus cell association Nr5a1-Adcyap1. In the initial paper describing the arcuate nucleus dataset[30], the Nr5a1-Adcyap1 cell population was described as arising from the VMH and projecting across the arcuate nucleus, raising the hypothesis that this could be a direct shared signal. Cells arising from the VMH and projecting across the arcuate nucleus have been shown to express estrogen

receptor alpha (ERα) and control glucose balance[34] and potentially be regulated by estrogen levels.[35] The gene encoding ERα, *ESR1*, is one of our GWAS hits for GDM, and estrogen is known to be elevated significantly in pregnancy.

The conditional analysis of cell specificity also finds that the significantly associated pancreatic cells represent two signals but with some signal overlap (Supplementary Figure 31 Supplementary Tables 33-34). The appearance of both murine beta cells and ductal cells as independent but related signals is interesting given ongoing recent work elucidating the role of ductal cells in murine beta cell compensation or expansion during pregnancy[36,37]. Signal in the pancreatic cell types appear distinct from that in the cells of the hypothalamus or arcuate nucelii and in fact that the PS > 1 implies that these signals appear stronger after accounting for signals in the brain (and vice versa).

Finally, the structure of the mouse pancreas has known anatomic, physiologic, and molecular differences from the human pancreas. To compare the relationship of GDM summary statistics with human vs murine pancreatic expression, we performed the initial cell-specificity analysis for both human and mouse pancreatic data (Supplementary Figure 32, Supplementary Table 35). Significant differences in the cell-tissue relationships were seen in human vs murine scRNA expression. GDM was primarily associated with beta cells in the mouse but delta cells in human studies. This analysis of pancreatic cells alone does not show the association with ductal cells in mouse observed in analysis with the full 22 tissue Tabula Muris data, possibly indicating that some of that signal is associated with average expression across pancreatic cell types. In either case, larger human scRNA studies and additional characterization of the signal in mouse cells will be required to elucidate how the relevant aspects of GDM genetics manifest at the cellular level in the pancreas in mice and humans.

Supplementary Figures

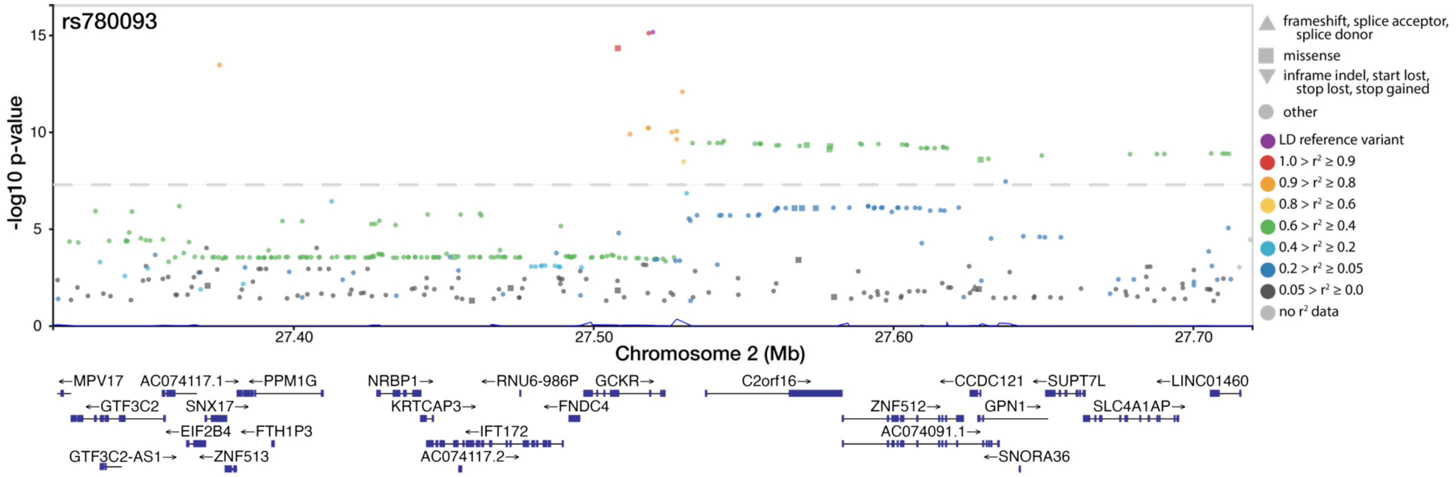

Supplementary Figure 1: Regional GWAS results for rs780093 locus on chromosome 2 at 27519736.
SNP label is the lead posterior inclusion probability (PIP) SNP from fine mapping. Unadjusted two-sided p-values from GDM GWAS using REGENIE 2.2.4 with firth correction. Dashed reference line is Bonferroni significance threshold (p = 5E-08). Color indicates LD with the lowest p-value SNP in the region. LD is computed from the Finnish SISu v3 reference panel. Shape indicates annotated functional consequence. Genes in the region from GENCODE are annotated below the X-axis.

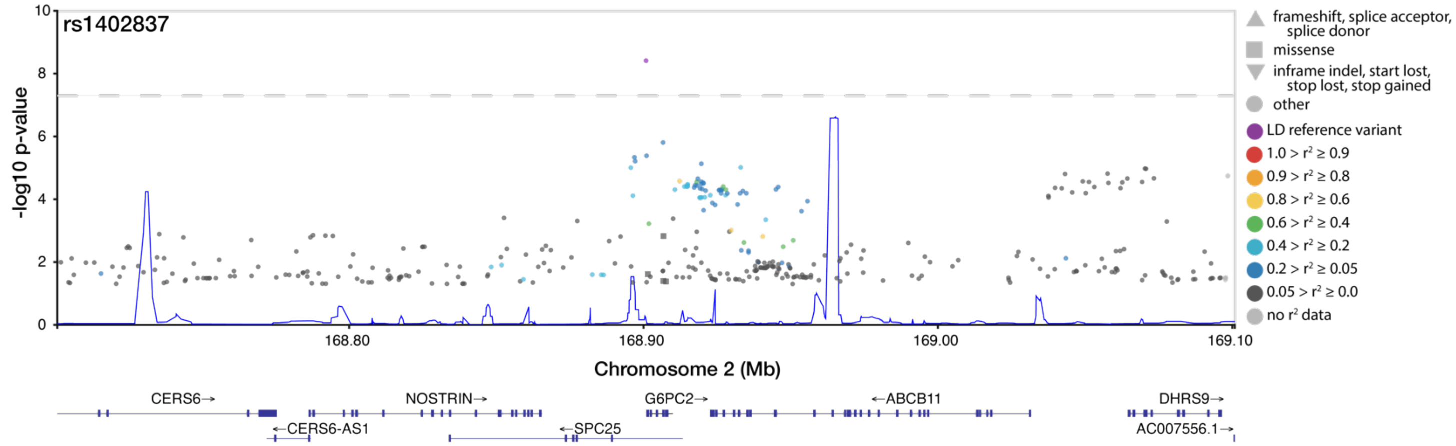

Supplementary Figure 2: Regional GWAS results for rs1402837 locus on chromosome 2 at 168900844.
SNP label is the lead posterior inclusion probability (PIP) SNP from fine mapping. Unadjusted two-sided p-values from GDM GWAS using REGENIE 2.2.4 with firth correction. Dashed reference line is Bonferroni significance threshold (p = 5E-08). Color indicates LD with the lowest p-value SNP in the region. LD is computed from the Finnish SISu v3 reference panel. Shape indicates annotated functional consequence. Genes in the region from GENCODE are annotated below the X-axis.

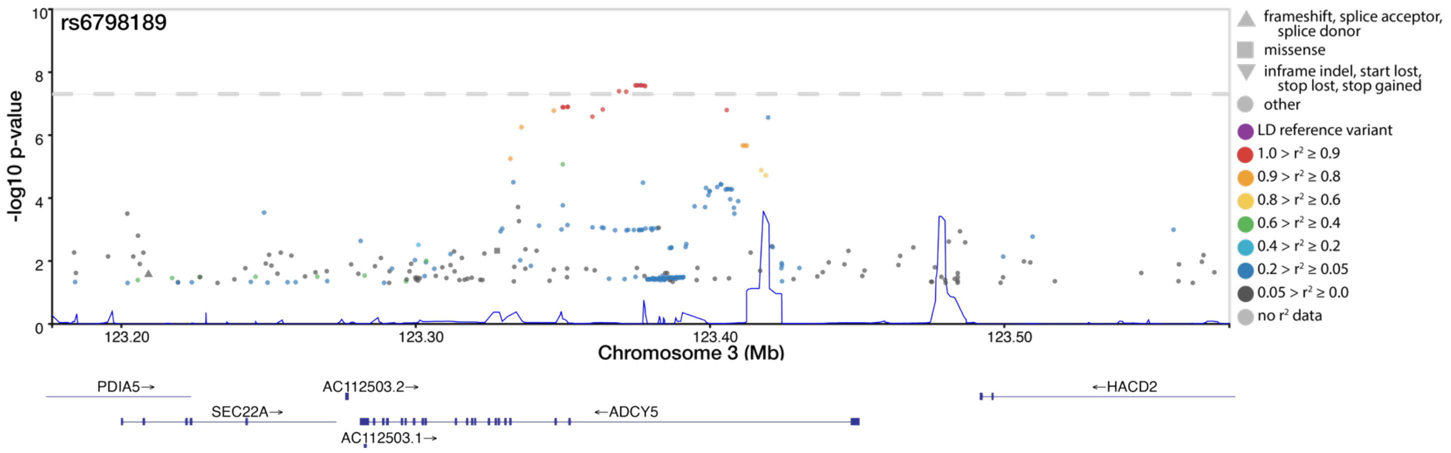

Supplementary Figure 3: Regional GWAS results for rs6798189 locus on chromosome 3 at 123376465.
SNP label is the lead posterior inclusion probability (PIP) SNP from fine mapping. Unadjusted two-sided p-values from GDM GWAS using REGENIE 2.2.4 with firth correction. Dashed reference line is Bonferroni significance threshold (p = 5E-08). Color indicates LD with the lowest p-value SNP in the region. LD is computed from the Finnish SISu v3 reference panel. Shape indicates annotated functional consequence. Genes in the region from GENCODE are annotated below the X-axis.

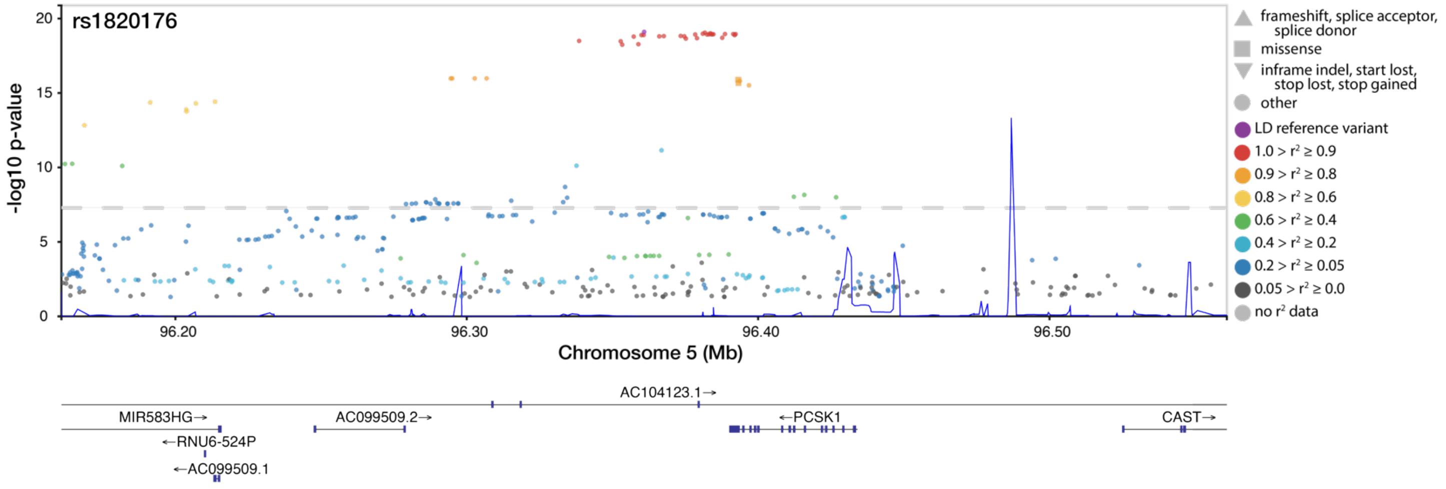

Supplementary Figure 4: Regional GWAS results for rs1820176 locus on chromosome 5 at 96360881.
SNP label is the lead posterior inclusion probability (PIP) SNP from fine mapping. Unadjusted two-sided p-values from GDM GWAS using REGENIE 2.2.4 with firth correction. Dashed reference line is Bonferroni significance threshold (p = 5E-08). Color indicates LD with the lowest p-value SNP in the region. LD is computed from the Finnish SISu v3 reference panel. Shape indicates annotated functional consequence. Genes in the region from GENCODE are annotated below the X-axis.

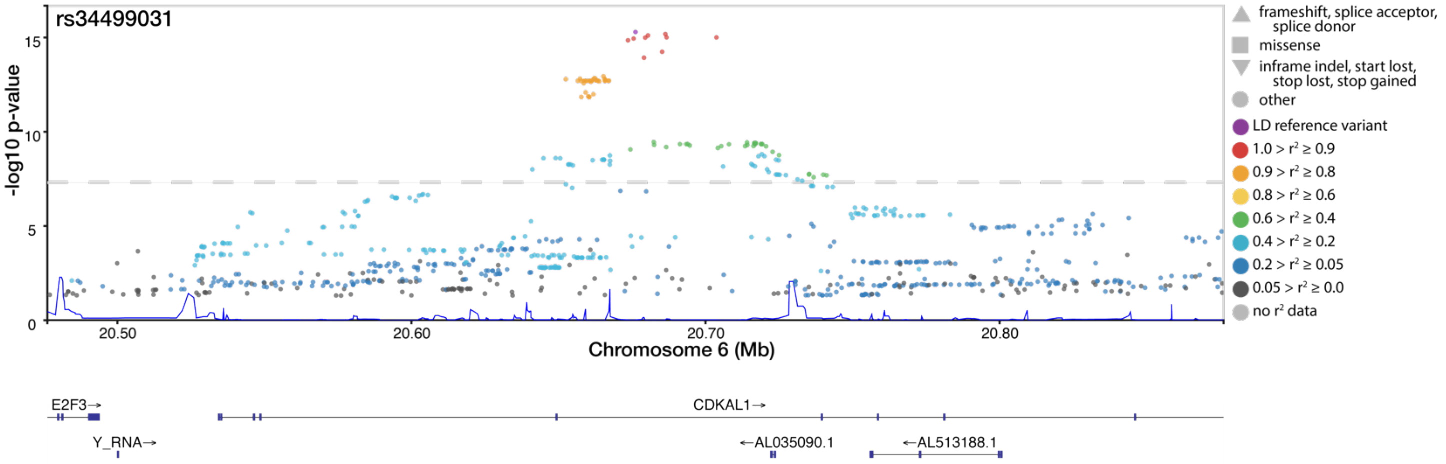

Supplementary Figure 5: Regional GWAS results for rs34499031 locus on chromosome 6 at 20676183.
SNP label is the lead posterior inclusion probability (PIP) SNP from fine mapping. Unadjusted two-sided p-values from GDM GWAS using REGENIE 2.2.4 with firth correction. Dashed reference line is Bonferroni significance threshold (p = 5E-08). Color indicates LD with the lowest p-value SNP in the region. LD is computed from the Finnish SISu v3 reference panel. Shape indicates annotated functional consequence. Genes in the region from GENCODE are annotated below the X-axis.

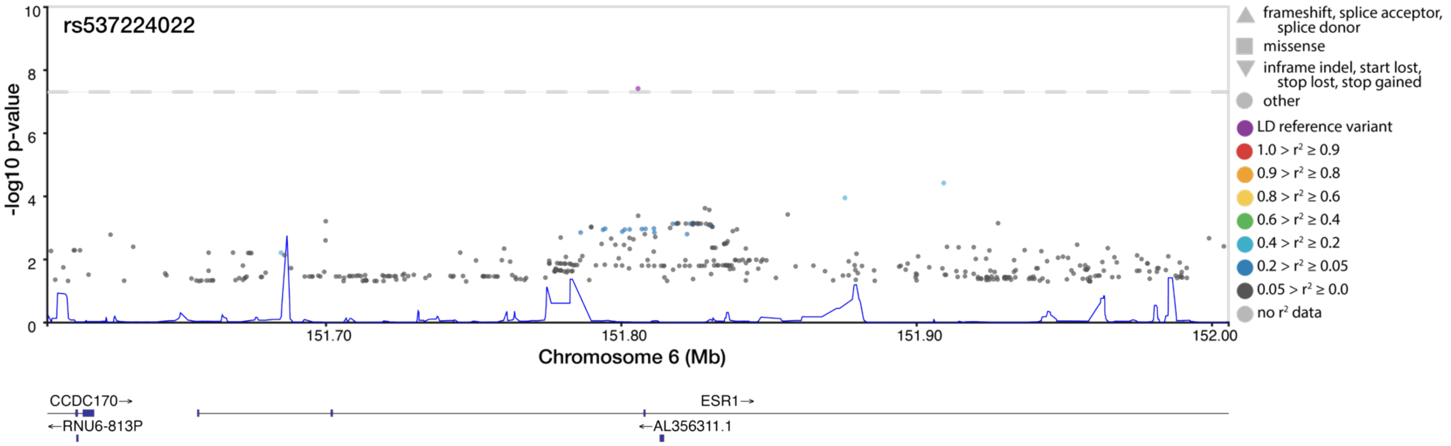

Supplementary Figure 6: Regional GWAS results for rs537224022 locus on chromosome 6 at 151805650.
SNP label is the lead posterior inclusion probability (PIP) SNP from fine mapping. Unadjusted two-sided p-values from GDM GWAS using REGENIE 2.2.4 with firth correction. Dashed reference line is Bonferroni significance threshold (p = 5E-08). Color indicates LD with the lowest p-value SNP in the region. LD is computed from the Finnish SISu v3 reference panel. Shape indicates annotated functional consequence. Genes in the region from GENCODE are annotated below the X-axis.

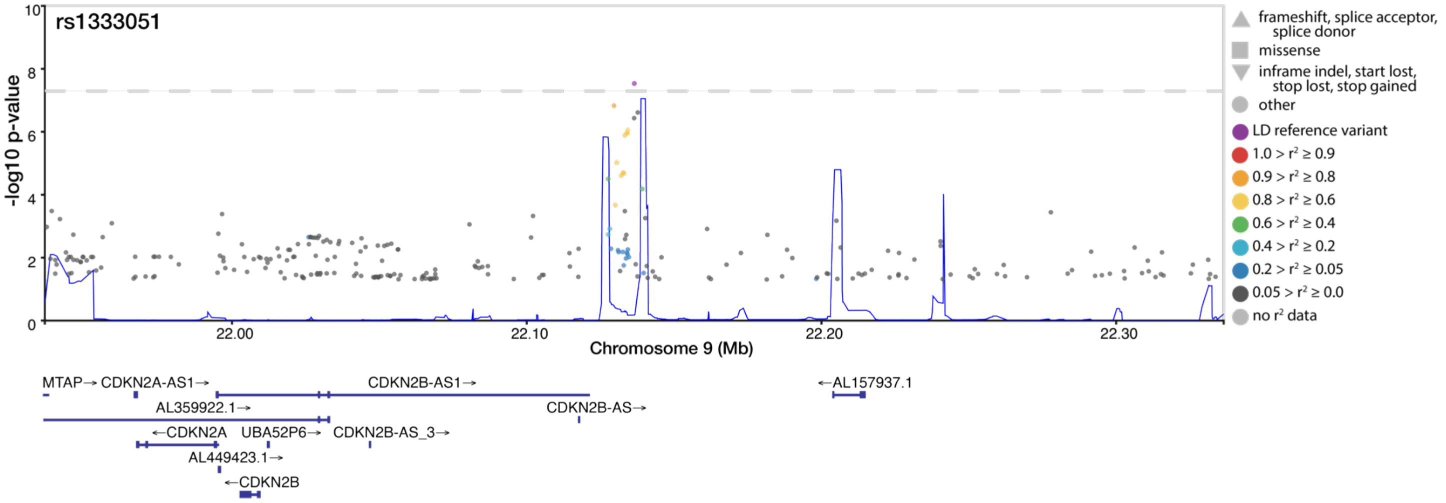

Supplementary Figure 7: Regional GWAS results for rs1333051 locus on chromosome 9 at 22136490.
SNP label is the lead posterior inclusion probability (PIP) SNP from fine mapping. Unadjusted two-sided p-values from GDM GWAS using REGENIE 2.2.4 with firth correction. Dashed reference line is Bonferroni significance threshold (p = 5E-08). Color indicates LD with the lowest p-value SNP in the region. LD is computed from the Finnish SISu v3 reference panel. Shape indicates annotated functional consequence. Genes in the region from GENCODE are annotated below the X-axis.

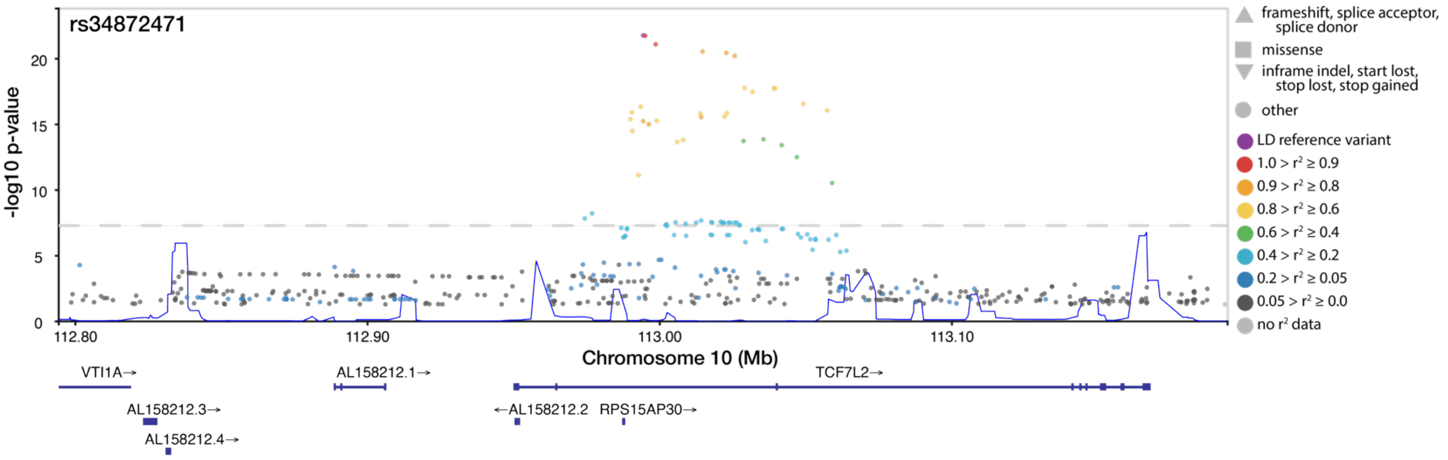

Supplementary Figure 8: Regional GWAS results for rs34872471 locus on chromosome 10 at 112994312.
SNP label is the lead posterior inclusion probability (PIP) SNP from fine mapping. Unadjusted two-sided p-values from GDM GWAS using REGENIE 2.2.4 with firth correction. Dashed reference line is Bonferroni significance threshold (p = 5E-08). Color indicates LD with the lowest p-value SNP in the region. LD is computed from the Finnish SISu v3 reference panel. Shape indicates annotated functional consequence. Genes in the region from GENCODE are annotated below the X-axis.

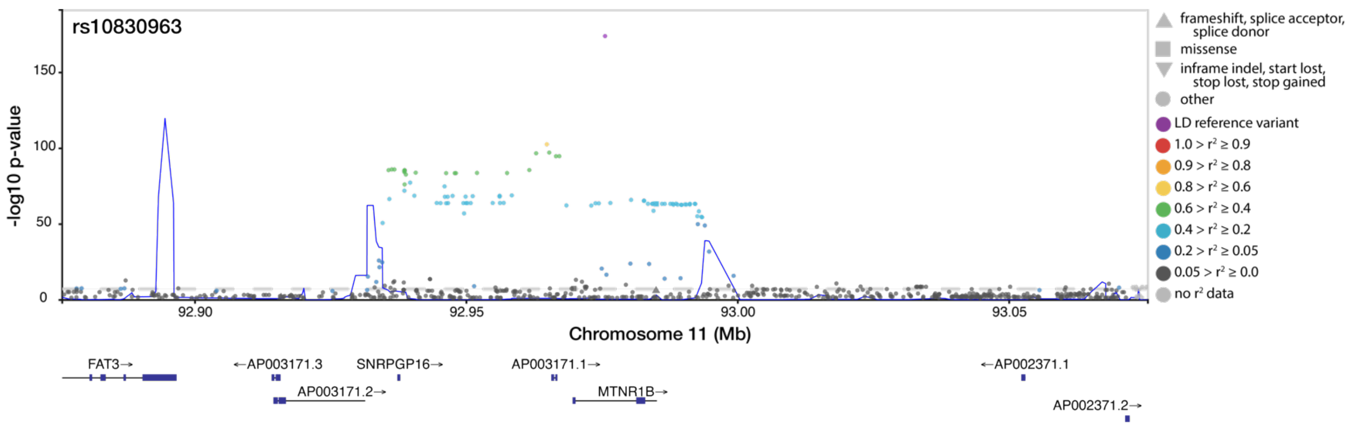

Supplementary Figure 9: Regional GWAS results for rs10830963 locus on chromosome 11 at 92975544.

SNP label is the lead posterior inclusion probability (PIP) SNP from fine mapping. Unadjusted two-sided p-values from GDM GWAS using REGENIE 2.2.4 with firth correction. Dashed reference line is Bonferroni significance threshold (p = 5E-08). Color indicates LD with the lowest p-value SNP in the region. LD is computed from the Finnish SISu v3 reference panel. Shape indicates annotated functional consequence. Genes in the region from GENCODE are annotated below the X-axis.

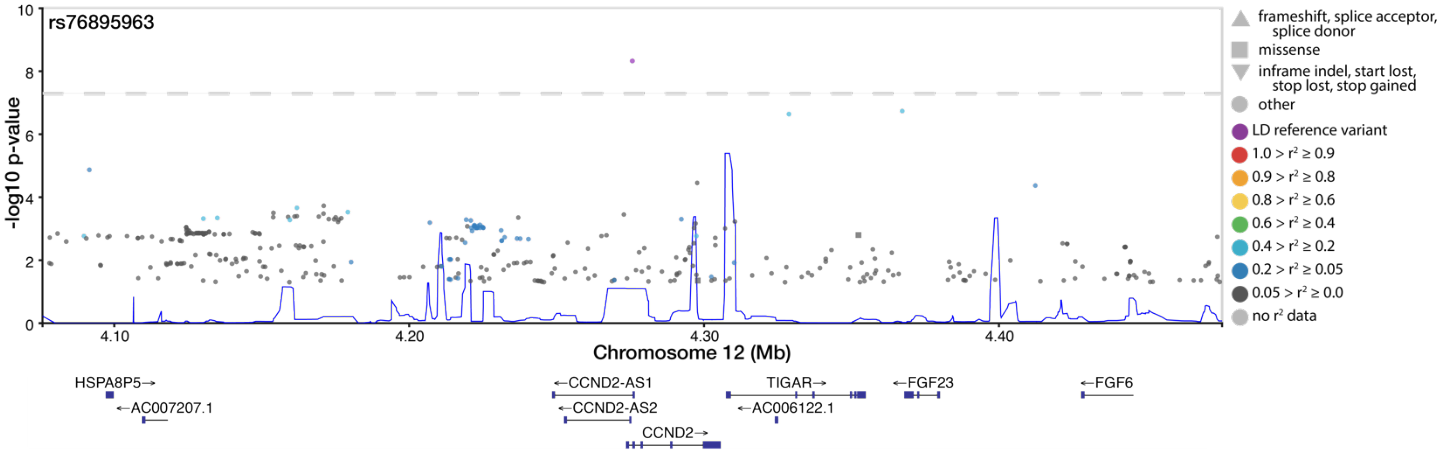

Supplementary Figure 10: Regional GWAS results for rs76895963 locus on chromosome 12 at 4275678.

SNP label is the lead posterior inclusion probability (PIP) SNP from fine mapping. Unadjusted two-sided p-values from GDM GWAS using REGENIE 2.2.4 with firth correction. Dashed reference line is Bonferroni significance threshold (p = 5E-08). Color indicates LD with the lowest p-value SNP in the region. LD is computed from the Finnish SISu v3 reference panel. Shape indicates annotated functional consequence. Genes in the region from GENCODE are annotated below the X-axis.

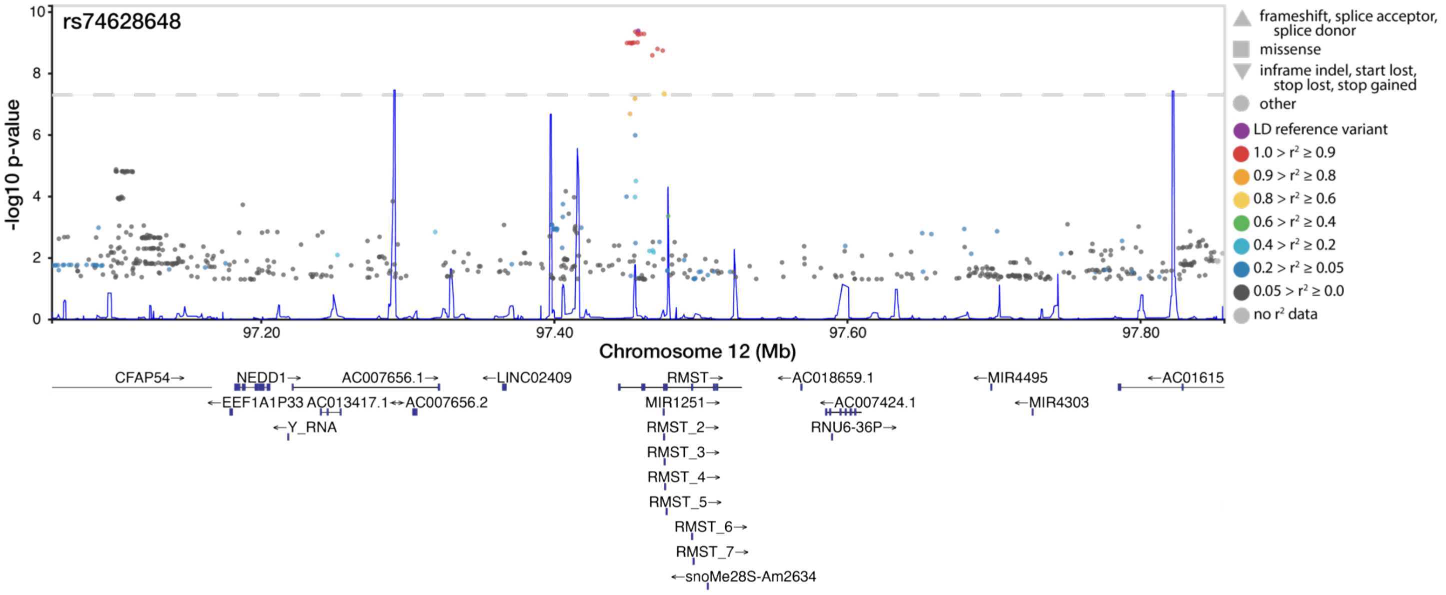

Supplementary Figure 11: Regional GWAS results for rs74628648 locus on chromosome 12 at 97457224.
SNP label is the lead posterior inclusion probability (PIP) SNP from fine mapping. Unadjusted two-sided p-values from GDM GWAS using REGENIE 2.2.4 with firth correction. Dashed reference line is Bonferroni significance threshold (p = 5E-08). Color indicates LD with the lowest p-value SNP in the region. LD is computed from the Finnish SISu v3 reference panel. Shape indicates annotated functional consequence. Genes in the region from GENCODE are annotated below the X-axis.

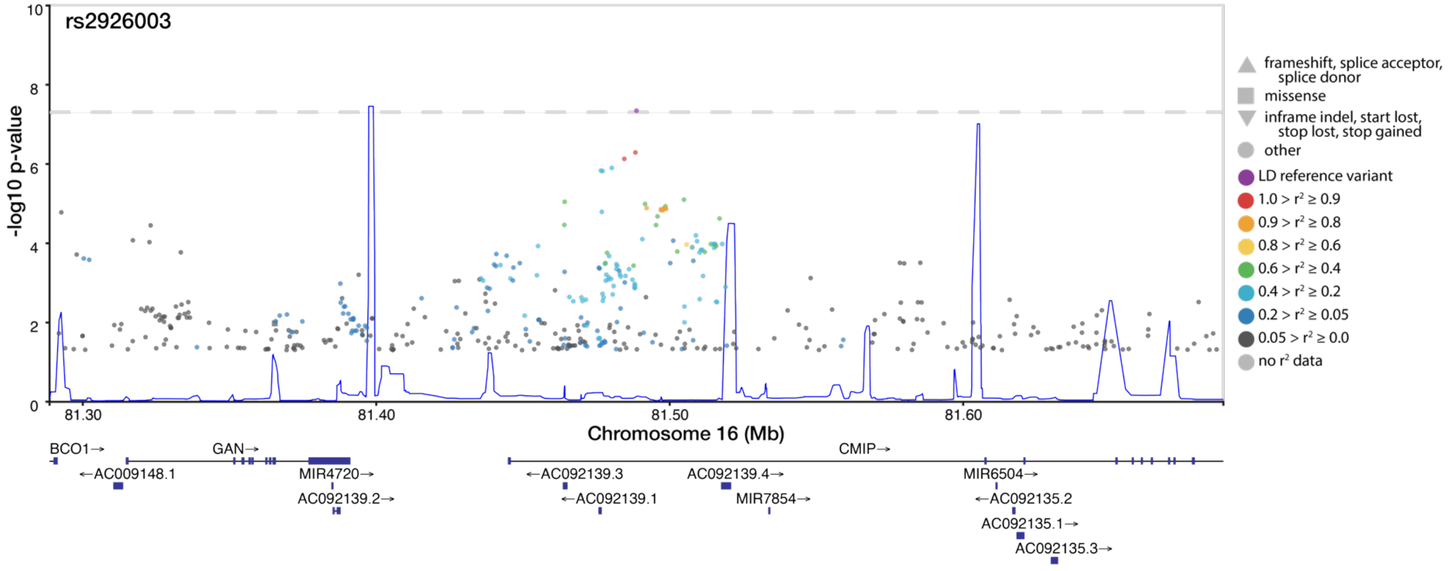

Supplementary Figure 12: Regional GWAS results for rs2926003 locus on chromosome 16 at 81488676.
SNP label is the lead posterior inclusion probability (PIP) SNP from fine mapping. Unadjusted two-sided p-values from GDM GWAS using REGENIE 2.2.4 with firth correction. Dashed reference line is Bonferroni significance threshold (p = 5E-08). Color indicates LD with the lowest p-value SNP in the region. LD is computed from the Finnish SISu v3 reference panel. Shape indicates annotated functional consequence. Genes in the region from GENCODE are annotated below the X-axis.

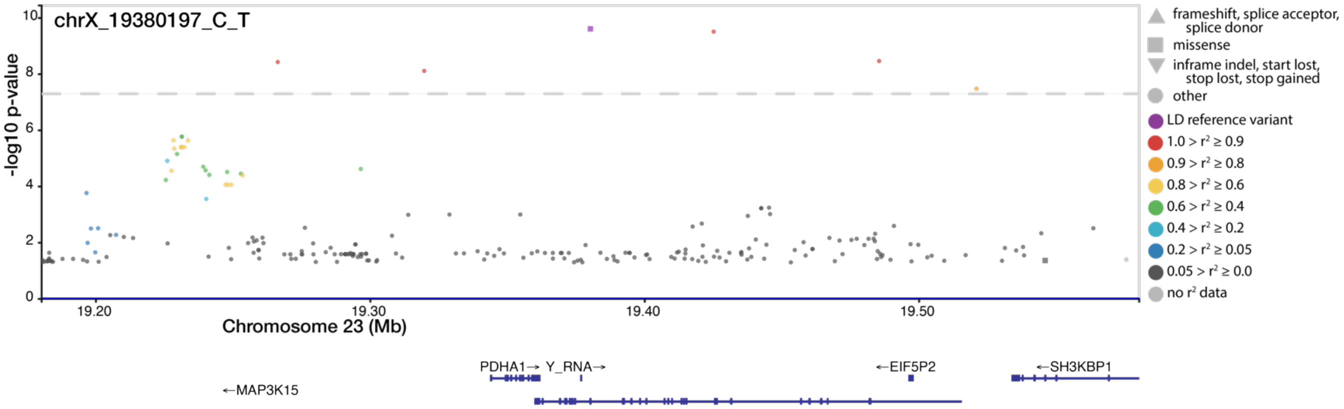

Supplementary Figure 13: Regional GWAS results for rs56381411 locus on chromosome 23 at 19380197.
SNP label is the lead posterior inclusion probability (PIP) SNP from fine mapping. Unadjusted two-sided p-values from GDM GWAS using REGENIE 2.2.4 with firth correction. Dashed reference line is Bonferroni significance threshold (p = 5E-08). Color indicates LD with the lowest p-value SNP in the region. LD is computed from the Finnish SISu v3 reference panel. Shape indicates annotated functional consequence. Genes in the region from GENCODE are annotated below the X-axis.

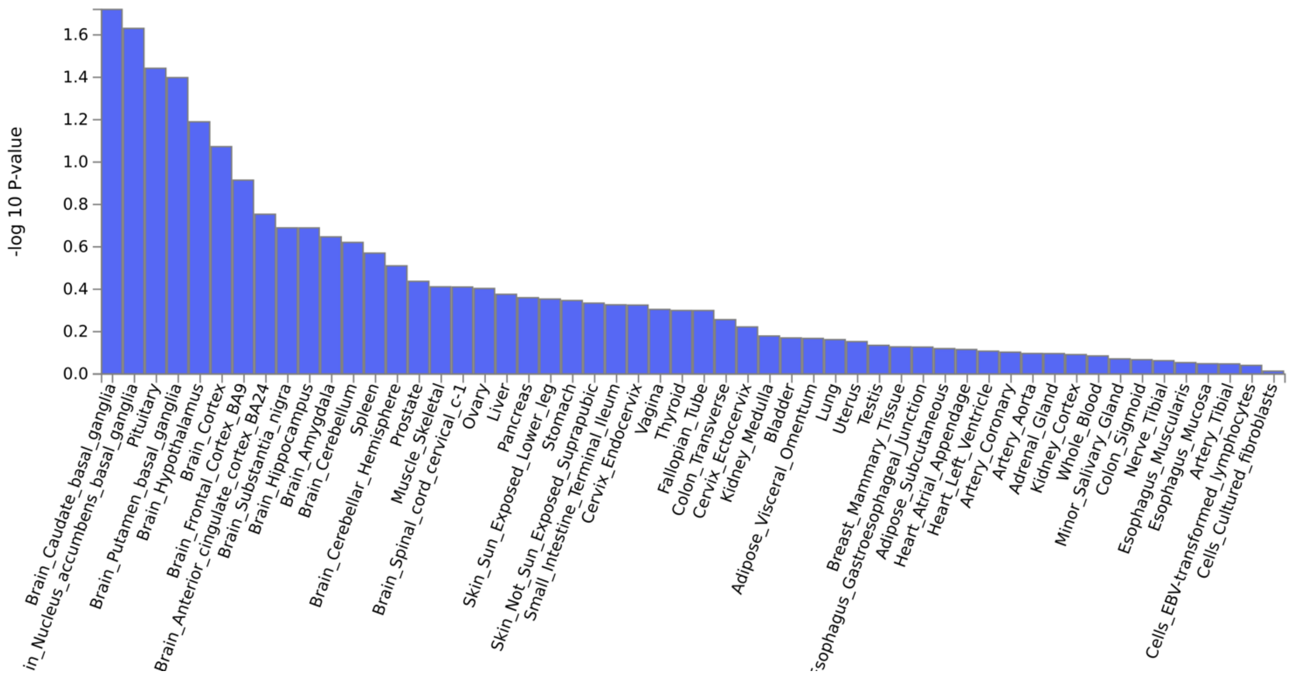

Supplementary Figure 14: Gene property analysis of tissue specific expression.
Unadjusted one-sided p-values from the FUMA cell property analysis v1.3.5d linear regression for association of MAGMA gene-level results for GDM on tissue specific gene expression across 17310 genes. Performed using FUMA SNP2GENE based on tissue specificity analysis of expression in GTEx. Data available in Supplementary Table 14.

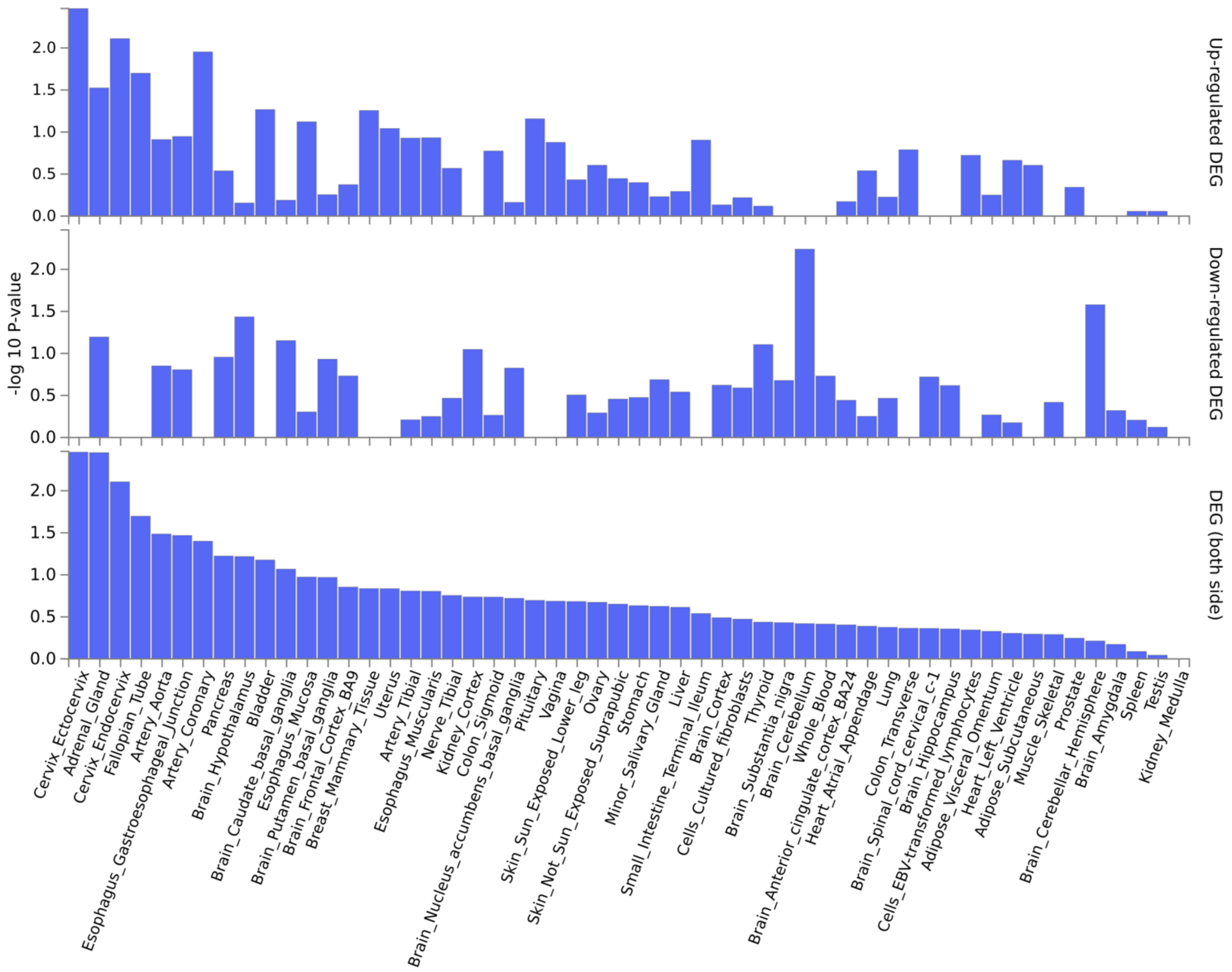

Supplementary Figure 15: Tissue specificity analysis of enrichment of differentially expressed gene sets (DEG).
Unadjusted p-values of enrichment of genes from SuSiE finemapping of GDM GWAS in gene sets for DEG in 54 tissue types in GTEx v8 using FUMA GENE2FUNC tool to perform a one-sided hypergeometric test per Online Methods. Panels correspond to enrichment for genes with (A) higher expression in a given tissue, (B) lower expression in a given tissue, (C) any expression change in a given tissue. No results are significant after correction for multiple testing. Data available in Supplementary Table 15.

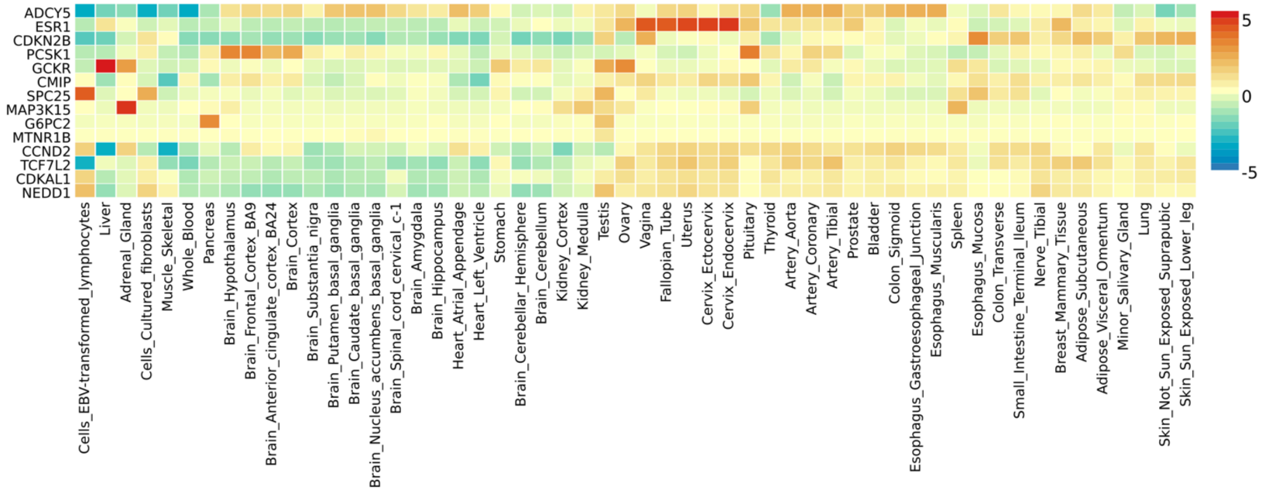

Supplementary Figure 16: Heat map of gene expression.
For each gene from finemapped GDM-associated loci, average log2(expression) in the given tissue compared to the average across tissues. Color scale is for intensity and direction of relative expression for gene labelled on Y-axis in tissue on X axis. Expression data from 54 tissues in GTEx analyzed using FUMA GENE2FUNC. GENE2FUNC with average of normalized expression per label.

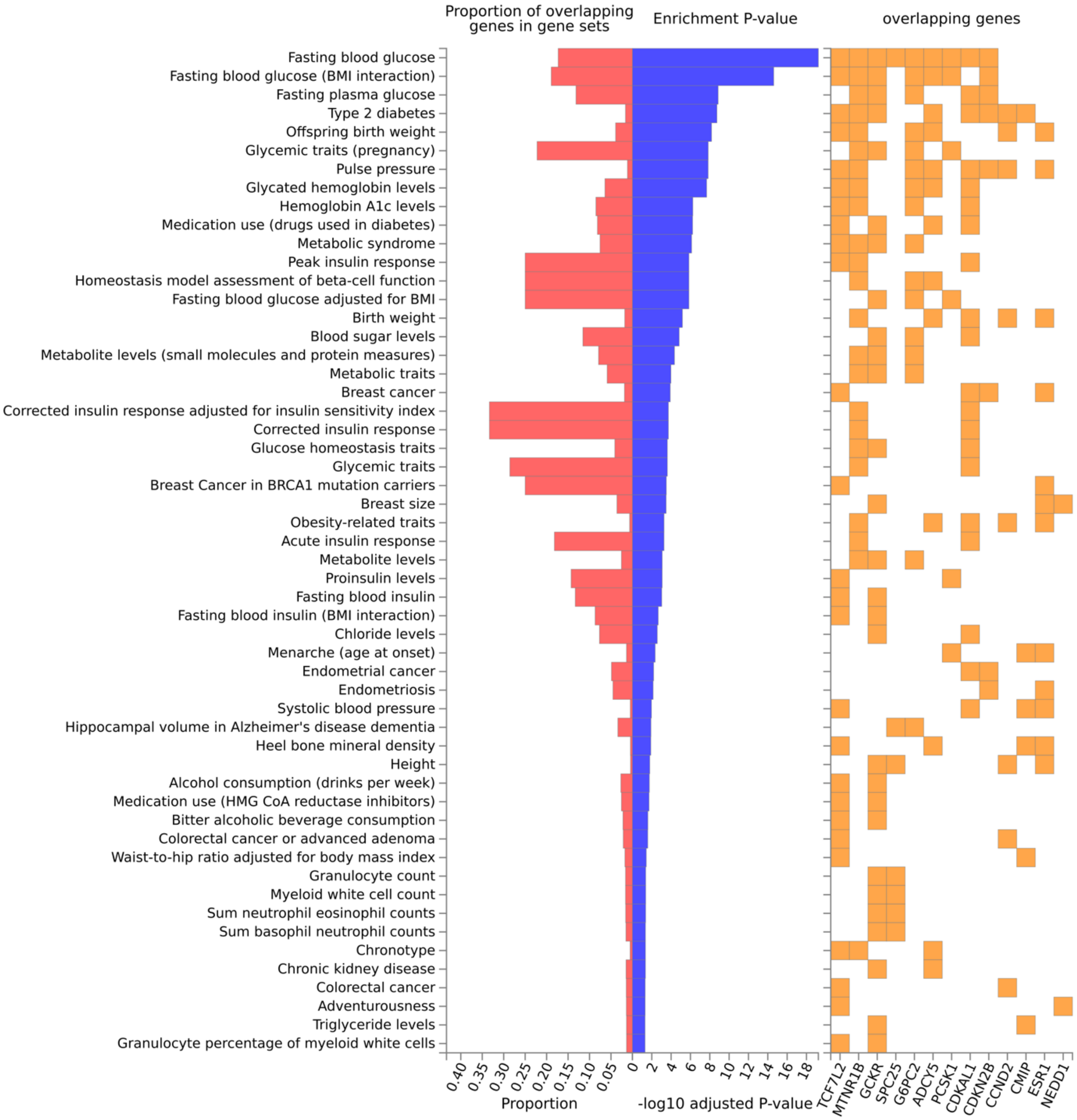

Supplementary Figure 17: Enrichment of annotated genes for finemapped GDM loci in GWAS Catalog gene sets.
MAGMA gene results from the GDM GWAS are tested for overrepresentation in gene sets using a one-sided hypergeometric test and showing only those with Bonferroni adjusted P-value < 0.05. Red bars indicate proportion of genes in the gene set that are among the GDM-related genes. Blue bars are the p-values after Bonferroni correction for number of tests. Yellow squares indicate overlapping genes. Performed using FUMA GENE2Func [https://fuma.ctglab.nl/tutorial#snp2gene]. Data available in Supplementary Table 16.

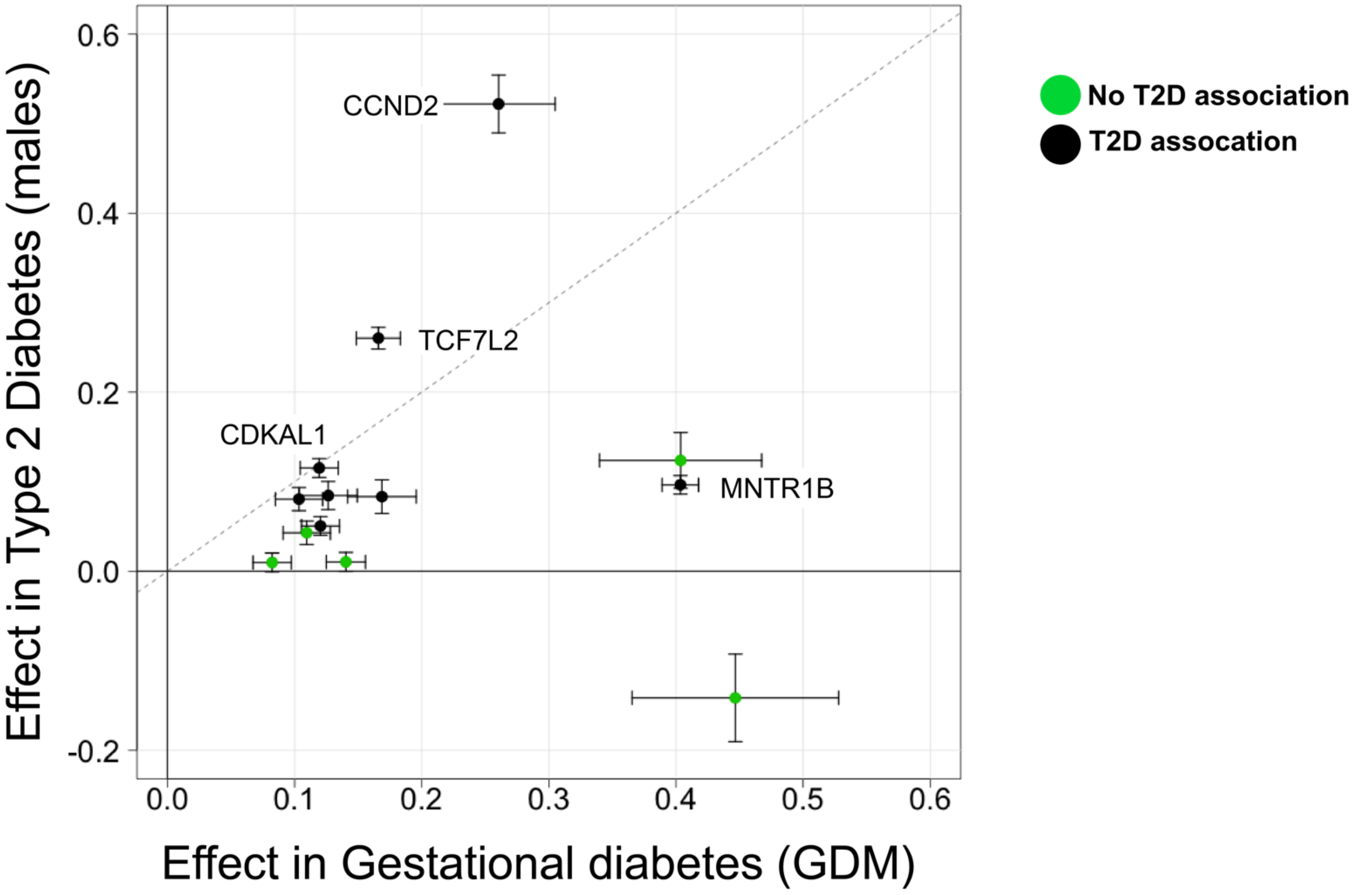

Supplementary Figure 18: Genetic effect comparison in GDM vs T2D for GDM-associated loci
The13 GDM-associated loci were then evaluated for their genetic effect in the GWAS of GDM (x-axis; 12,332 cases, 131,109 controls) versus in T2D in males (y-axis; 27,607 cases, 118,687 controls). GWAS were performed with REGENIE 2.2.4 with firth correction. Error bars reflect ±1 standard error. Heterogeneous appearance of effects was evident. The potential for components of effect was raised on gross examination – particularly given the more uniform appearance of many of the loci previously associated with T2D (black).

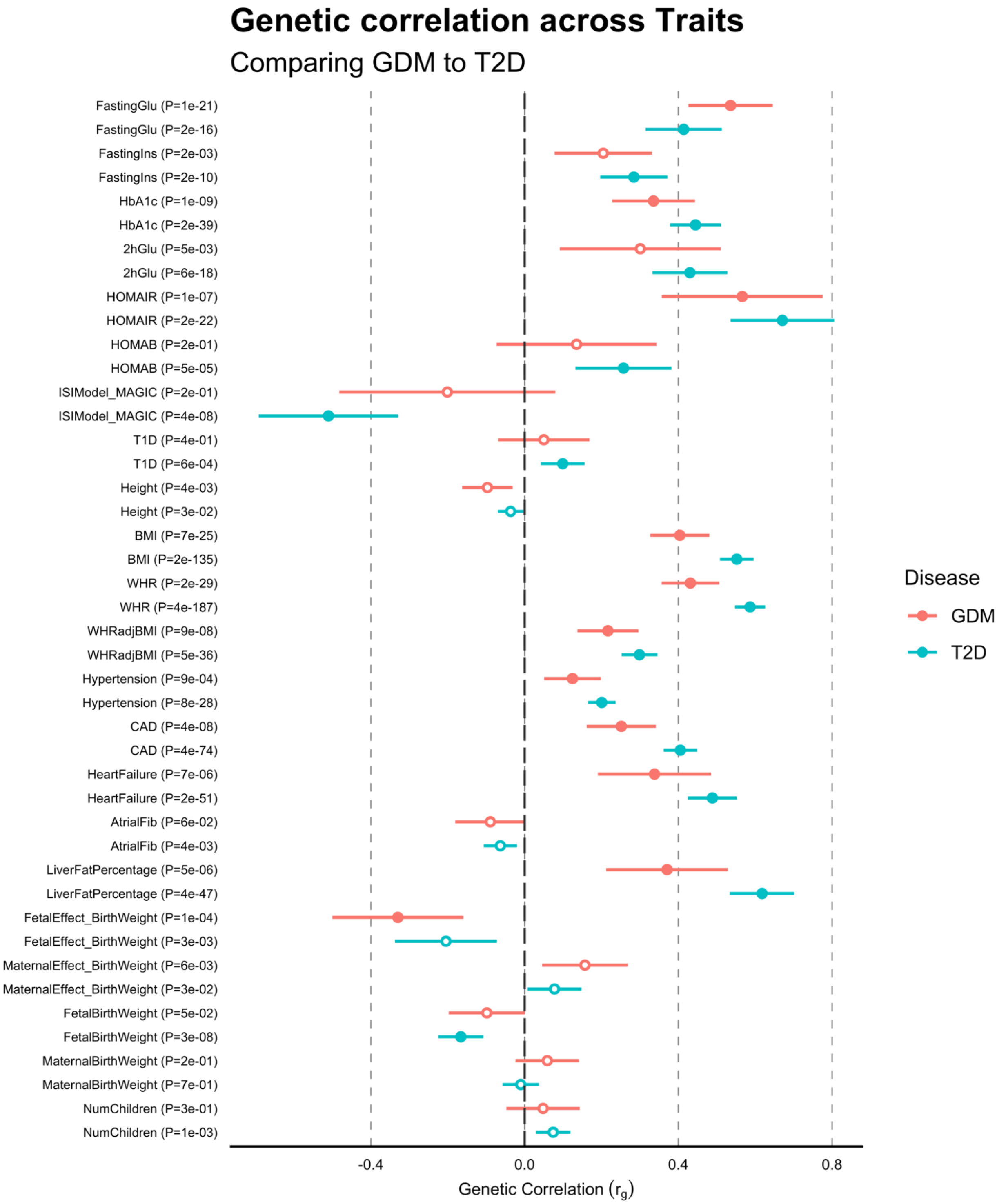

Supplementary Figure 19: Genetic correlation of GDM with 22 Traits.
The genetic correlations (SNP- r_g) between GDM (red; 12,332 cases, 131,109 controls) was computed with 22 traits using LD score regression (Supplementary Table 19) and compared to the SNP- r_g for T2D in FinnGen (Blue; 65,085 cases, 335,112 controls) against the same 22 traits. Error bars are 95% confidence intervals. Filled points indicate the two-tailed test of genetic correlation is significant after Bonferroni correction for multiple testing for both traits and biomarkers. The difference in r_g between GDM and T2D is significant in 3 traits after Bonferroni correction: BMI, Waist-to-hip ratio adjusted for BMI, and CAD (Supplementary Table 21).

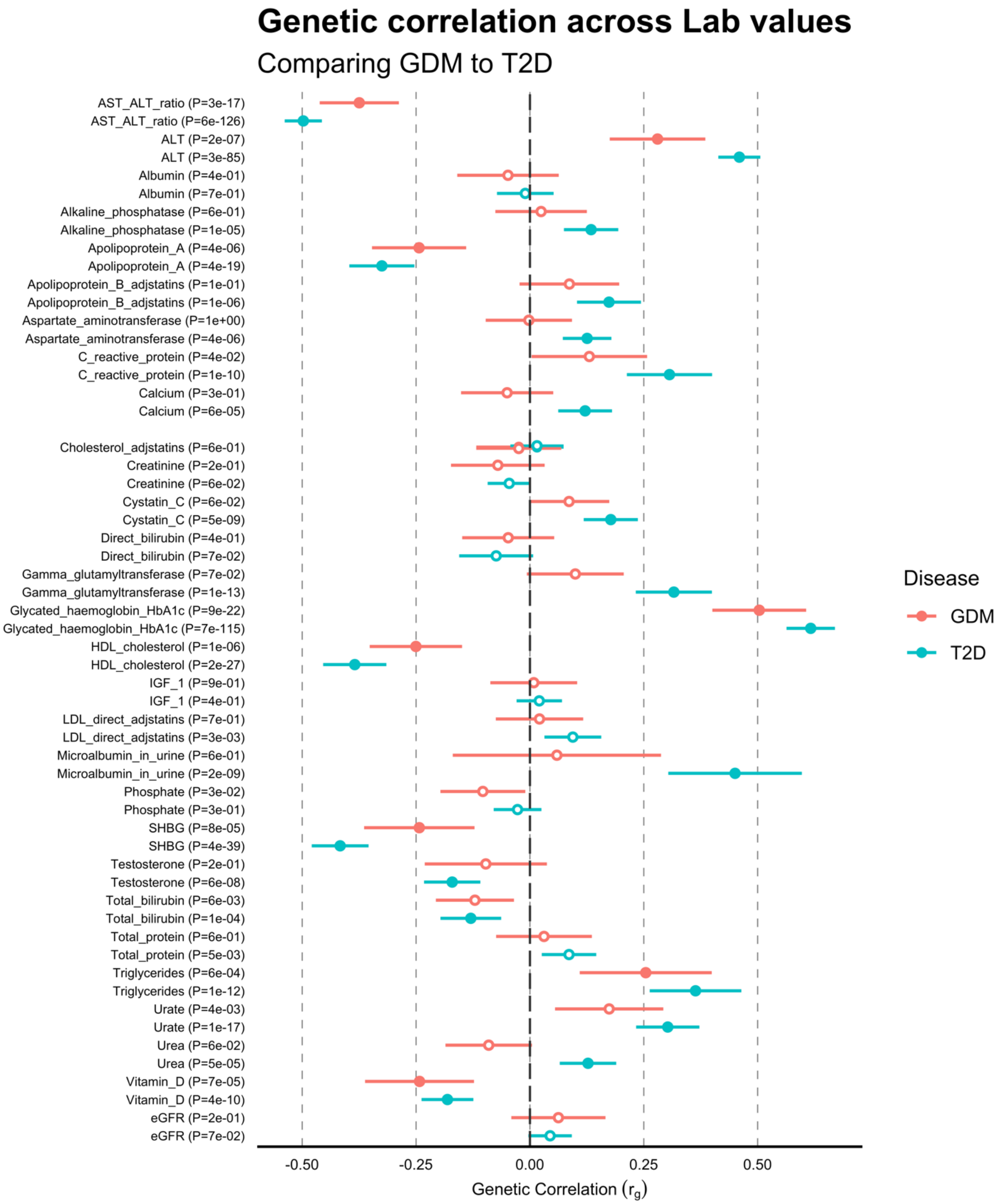

Supplementary Figure 20: Genetic correlation of GDM with 29 biomarkers.
The genetic correlation(SNP- r_g) between GDM (red; 12,332 cases, 131,109 controls) was computed with 29 biomarkers using LD score regression (Supplementary Table 20) and compared to the SNP- r_g for T2D in FinnGen (Blue; 65,085 cases, 335,112 controls) against the same 29 biomarkers. Error bars are 95% confidence intervals. Filled points indicate the two-tailed test of genetic correlation is significant after Bonferroni correction for multiple testing for both traits and biomarkers. The difference in r_g between GDM and T2D is significant in 1 biomarkers after Bonferroni correction: alanine transaminase (ALT; Supplementary Table 22).

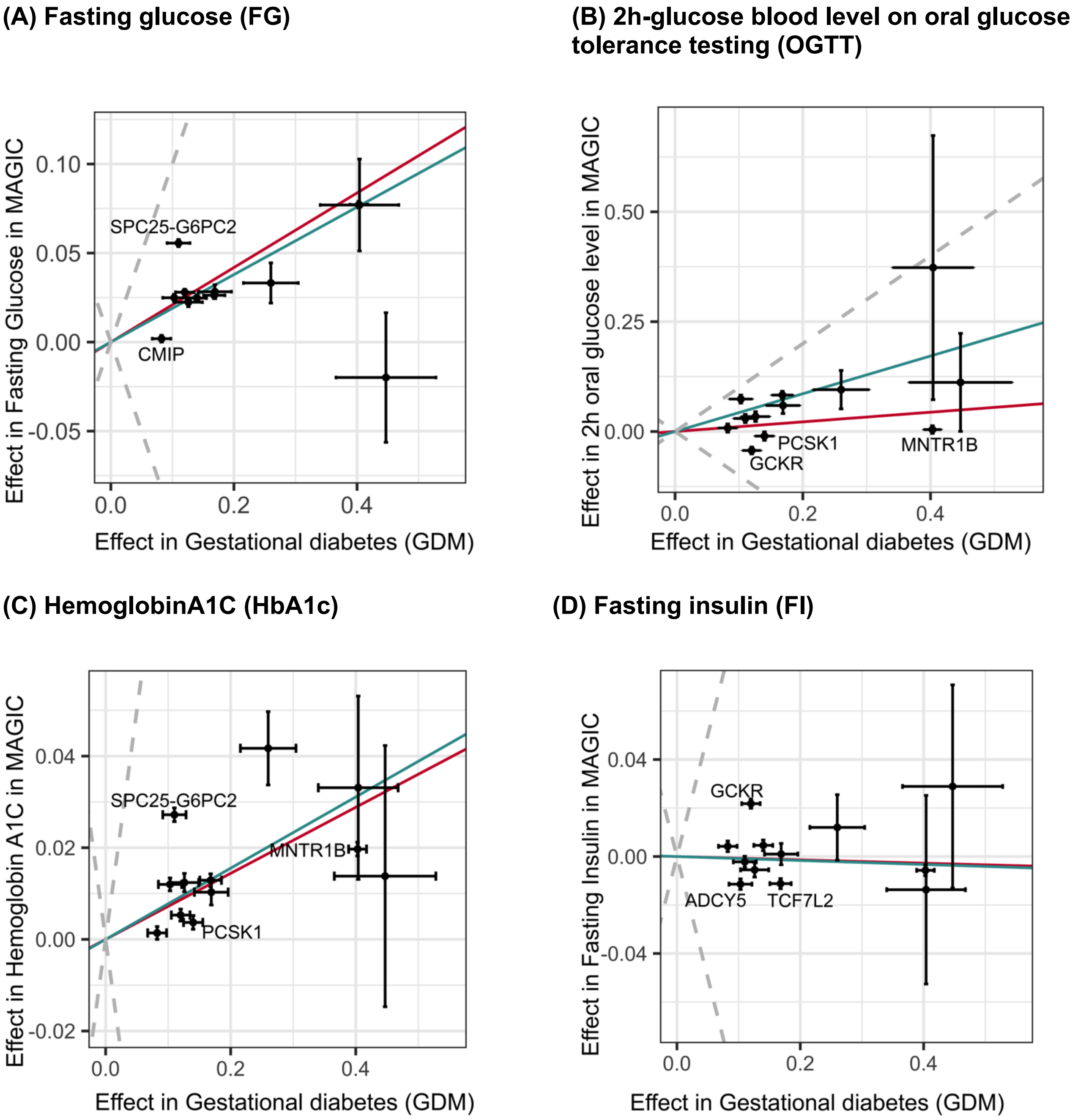

Supplementary Figure 21: Comparison of effects of GDM-associated loci in GDM vs. glycemic traits

The 13 GDM-associated loci were then evaluated for their log odds ratio (beta) in the GWAS of GDM (x-axis 12,332 cases, 131,109 controls) compared with the genetic effect in each of 4 glycemic traits from GWAS of European samples in the MAGIC consortia. Effects were compared to the genetic effects in (A) fasting glucose (N = 209,038), (B) 2 hour glucose (N = 157,983), (C) HbA1C (N = 64,469), and (D) fasting insulin (N = 149,006). Error bars indicate +/- 1 standard error in each GWAS. Points labelled with gene names are identified as outliers by SCOUTJOY. Fitted slopes are shown from York regression with all variants (red) and after removal of identified outliers (blue). Dashed gray reference lines indicate equal absolute effect size. SCOUTJOY analysis suggests significant heterogeneity in all 4

comparisons, and a significant positive relationship of GDM with fasting glucose, 2 hour glucose, and HbA1C after outlier removal (Supplementary Table 24).

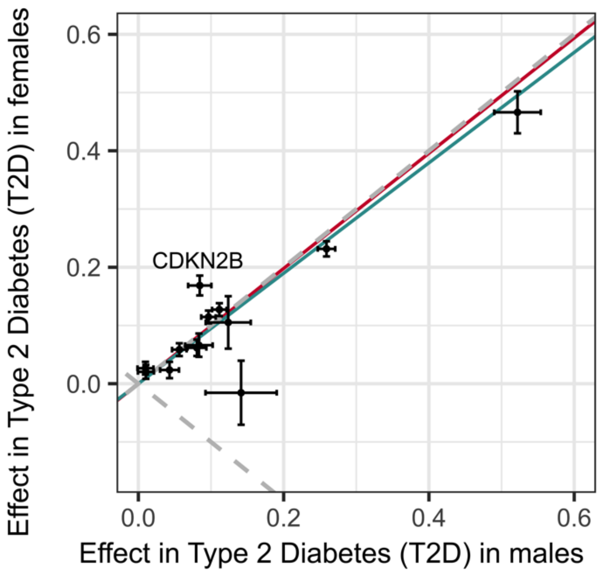

Supplementary Figure 22: Comparison of effects of GDM-associated loci in T2D in males vs T2D in females
The 13 GDM-associated loci were evaluated for whether their log odds ratio in T2D was different in males versus females by comparing a GWAS beta of T2D in males in FinnGen (x-axis; 27,607 cases, 118,687 controls) against the GWAS beta for T2D in females (y-axis; 21,507 cases, 156,472 controls). Error bars indicate +/- 1 standard error in each GWAS. Points labelled with gene names are identified as outliers by SCOUTJOY. Fitted slopes are shown from York regression with all variants (red) and after removal of identified outliers (blue). Dashed gray reference lines indicate equal absolute effect size. Data available in Supplementary Table 18,25-26.

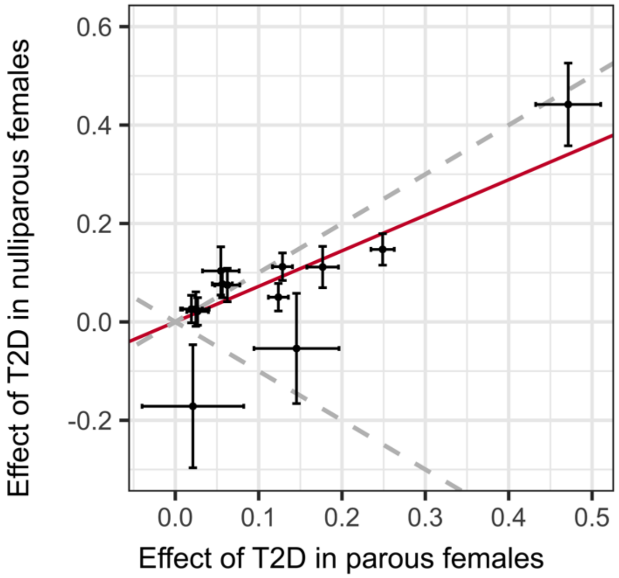

Supplementary Figure 23: Comparison of effects of GDM-associated loci in T2D in parous females vs T2D in nulliparous females
The 13 GDM-associated loci were evaluated for whether their log odds ratio in T2D was different in females with versus without a history of pregnancy by comparing the GWAS beta of T2D in parous females in FinnGen (x-axis; 17,912 cases, 124,357 controls) at these loci against the GWAS beta for T2D in nulliparous females (y-axis; 3,595 cases, 40,163 controls). Error bars indicate +/- 1 standard

error in each GWAS. No significant outliers are identified by SCOUTJOY. Fitted slope is from York regression with all variants. Dashed gray reference lines indicate equal absolute effect size. Data available in Supplementary Table 18,25-26.

(A) Males in Mahajan et al **(B) Females in Mahajan et al**

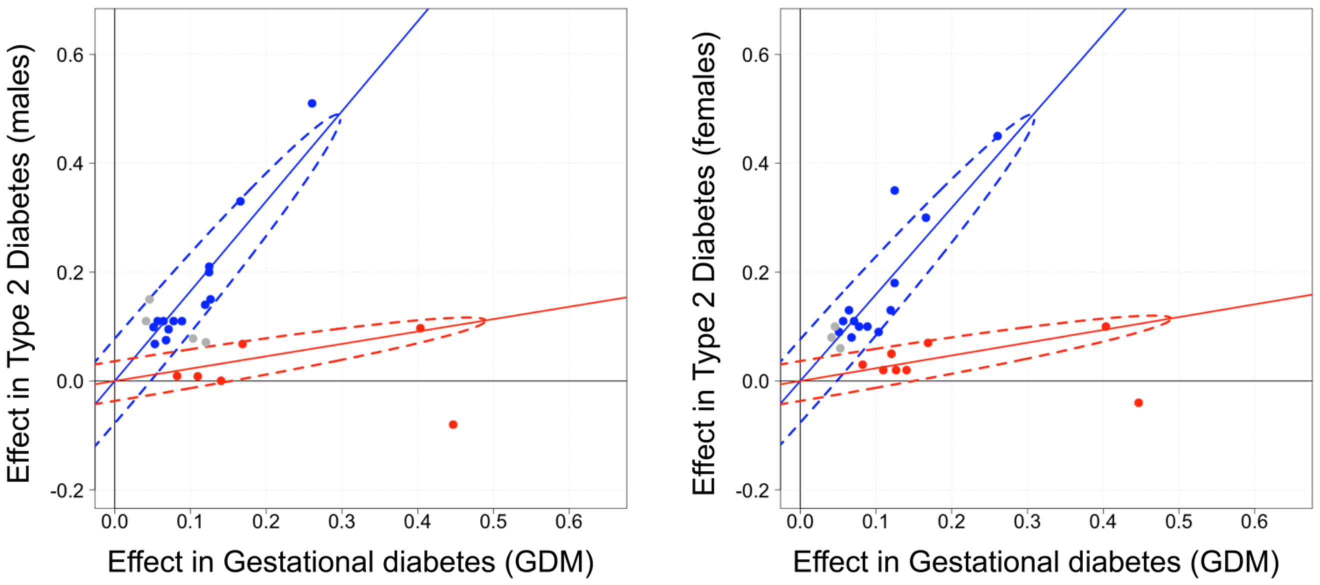

Supplementary Figure 24: Comparison of shared variants analysis of GDM-associated loci in GDM vs. T2D by sex
Betas (log odds ratios) from the GWAS of GDM (x-axis) for each of the 13 significantly associated loci are compared to the betas from GWAS of T2D (y-axis) from the Mahajan et al meta-analysis among (A) males or (B) females. Two distinct classes of SNP effects were identified by a Bayesian classifier in shared variants analysis: Class T (blue) containing SNPs with T2D-predominant genetic effects and Class G (red) with GDM-predominant effects (Supplementary Table 27). Grey SNPs were not confidently assigned to either class (posterior probability > 95%). Dotted ellipses indicate the 95% probability regions of the fitted bivariate effect size distributions with each class. Probability values for the groups are given in Supplementary Table 27.

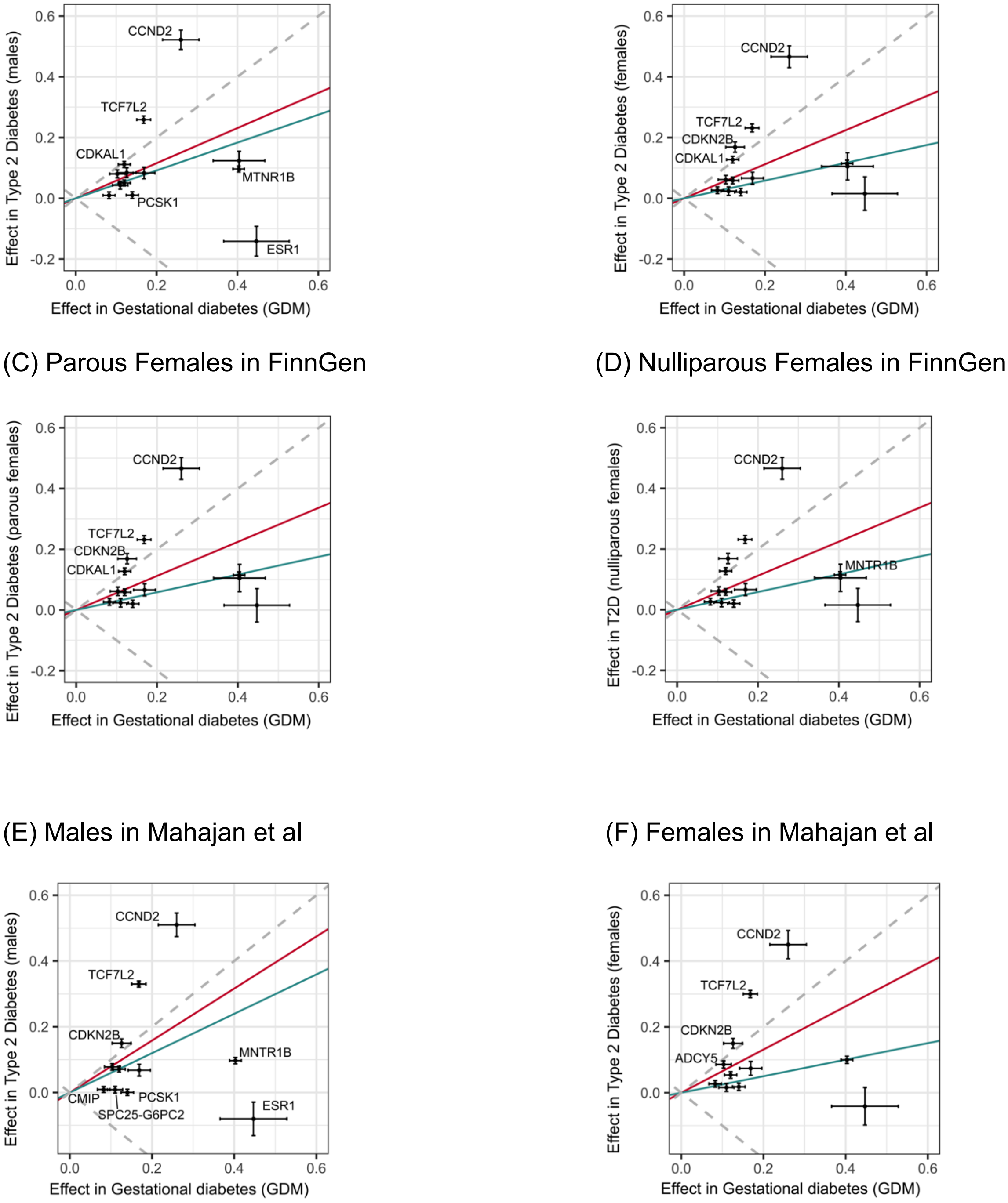

Supplementary Figure 25: Comparison of effects of GDM-associated loci in GDM vs. T2D subgroups by sex and pregnancy history

The 13 GDM-associated loci were evaluated for the relationship of the log odds ratio in GDM versus the effect in T2D with different sexes and pregnancy histories. For each locus,betas (log odds ratios) from the GWAS of GDM (12,332 cases, 131,109 controls) were compared to the betas from GWAS of T2D in FinnGen among (A) males (27,607 cases, 118,687 controls), (B) females (21,507 cases, 156,472 controls), (C) parous females (17,912 cases, 124,357 controls), and (D) nulliparous females (3,595 cases, 40,163 controls). Error bars indicate +/- 1 standard error in each GWAS. Points labelled with gene names are identified as outliers by SCOUTJOY. Fitted slopes are shown from York regression

with all variants (red) and after removal of identified outliers (blue). Dashed gray reference lines indicate equal absolute effect size. Data available in Supplementary Table 18.

(A) T2D in FinnGen

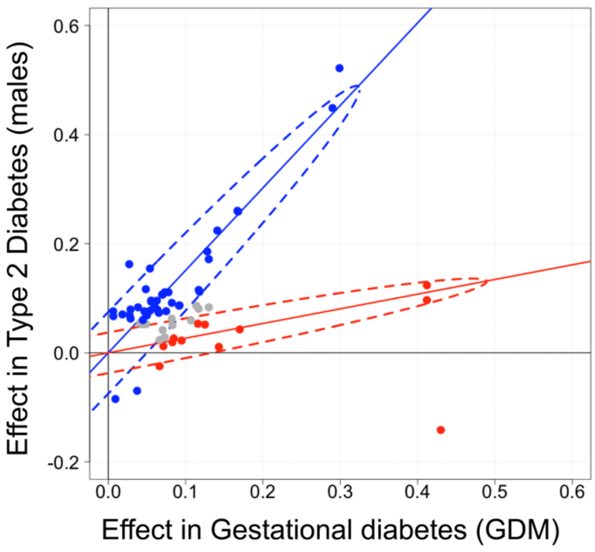

(B) Males in Mahajan et al

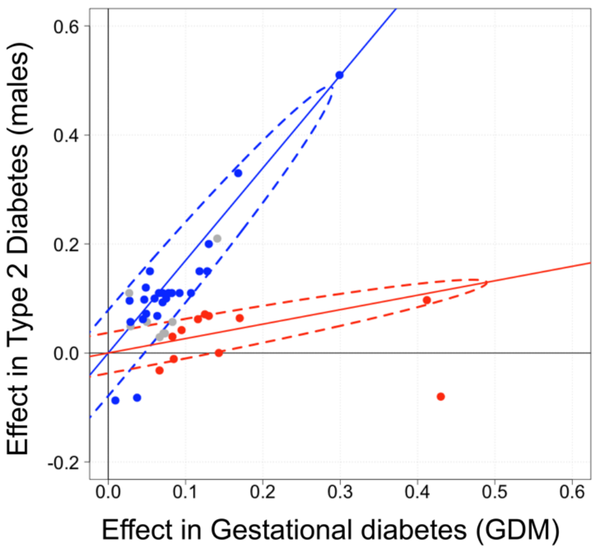

(C) Females in Mahajan et al

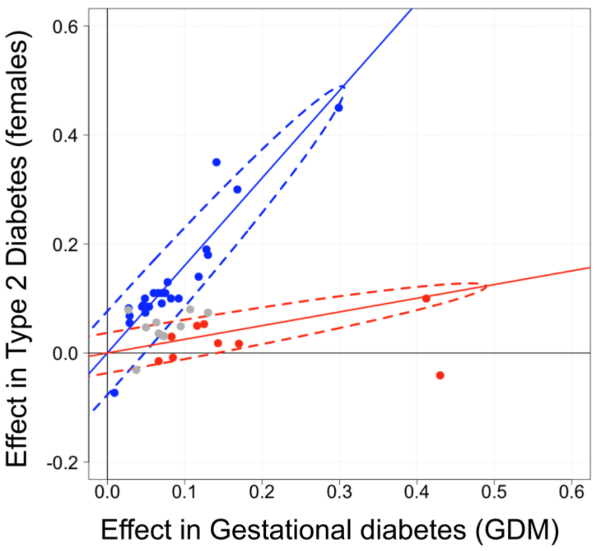

Supplementary Figure 26: Comparison of shared variants analysis of extended data (FinnGen Release 11) GDM-associated loci in GDM vs. T2D by sex

Betas (log odds ratios) from the GWAS of GDM (x-axis) for each of the 29 significantly associated loci are compared to the betas from GWAS of T2D (y-axis) from the Mahajan et al meta-analysis among (A) males or (B) females. Two distinct classes of SNP effects were identified by a Bayesian classifier in shared variants analysis: Class T (blue) containing SNPs with T2D-predominant genetic effects and Class G (red) with GDM-predominant effects (Supplementary Table 30). Grey SNPs were not confidently assigned to either class (posterior probability > 95%). Dotted ellipses indicate the 95% probability regions of the fitted bivariate effect size distributions with each class. Probability values for the groups are given in Supplementary Table 30

(A) T2D in FinnGen

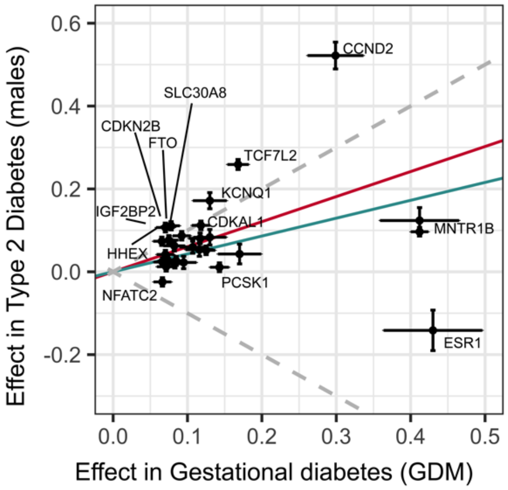

(B) Males in Mahajan et al

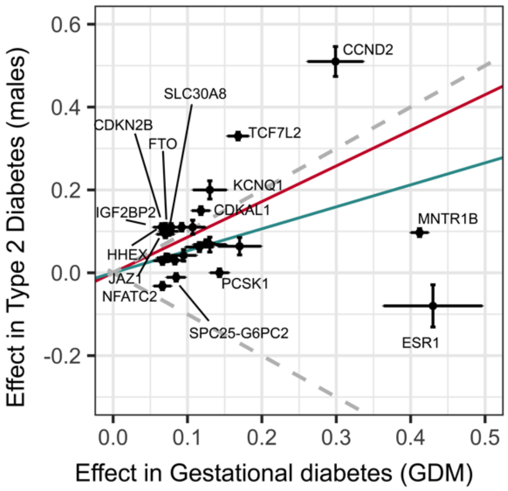

(C) Females in Mahajan et al

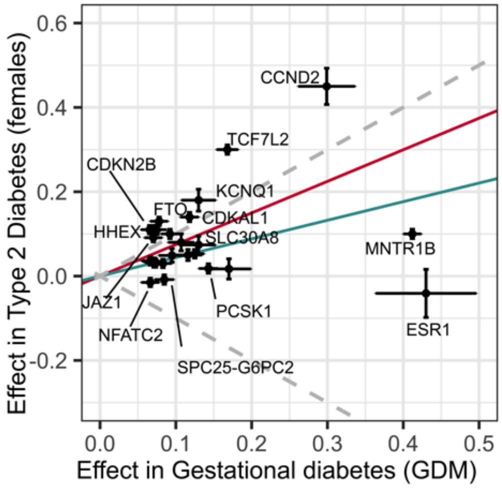

Supplementary Figure 27: Comparison of effects of GDM-associated loci from extended data (FinnGen Release 11) in GDM vs. T2D subgroups by sex

The 29 GDM-associated loci discovered in the extended FinnGen dataset (Supplementary Table 17) were evaluated for whether their log odds ratio in T2D was different in males versus females by comparing the GWAS betas (log odds ratios) of GDM in FinnGen extended data (x-axis, 18474 cases, 171349 controls) with the betas from GWAS of T2D (A) in FinnGen (65,085 cases, 335,112 controls) and in Mahajan et al in (B) males (reported effective N = 127,964), and (C) females (reported effective N = 86,065). Error bars indicate +/- 1 standard error in each GWAS. Points labelled with gene names are identified as outliers by SCOUTJOY. Fitted slopes are shown from York regression with all variants (red) and after removal of identified outliers (blue). Dashed gray reference lines indicate equal absolute effect size. Data available in Supplementary Table 29.

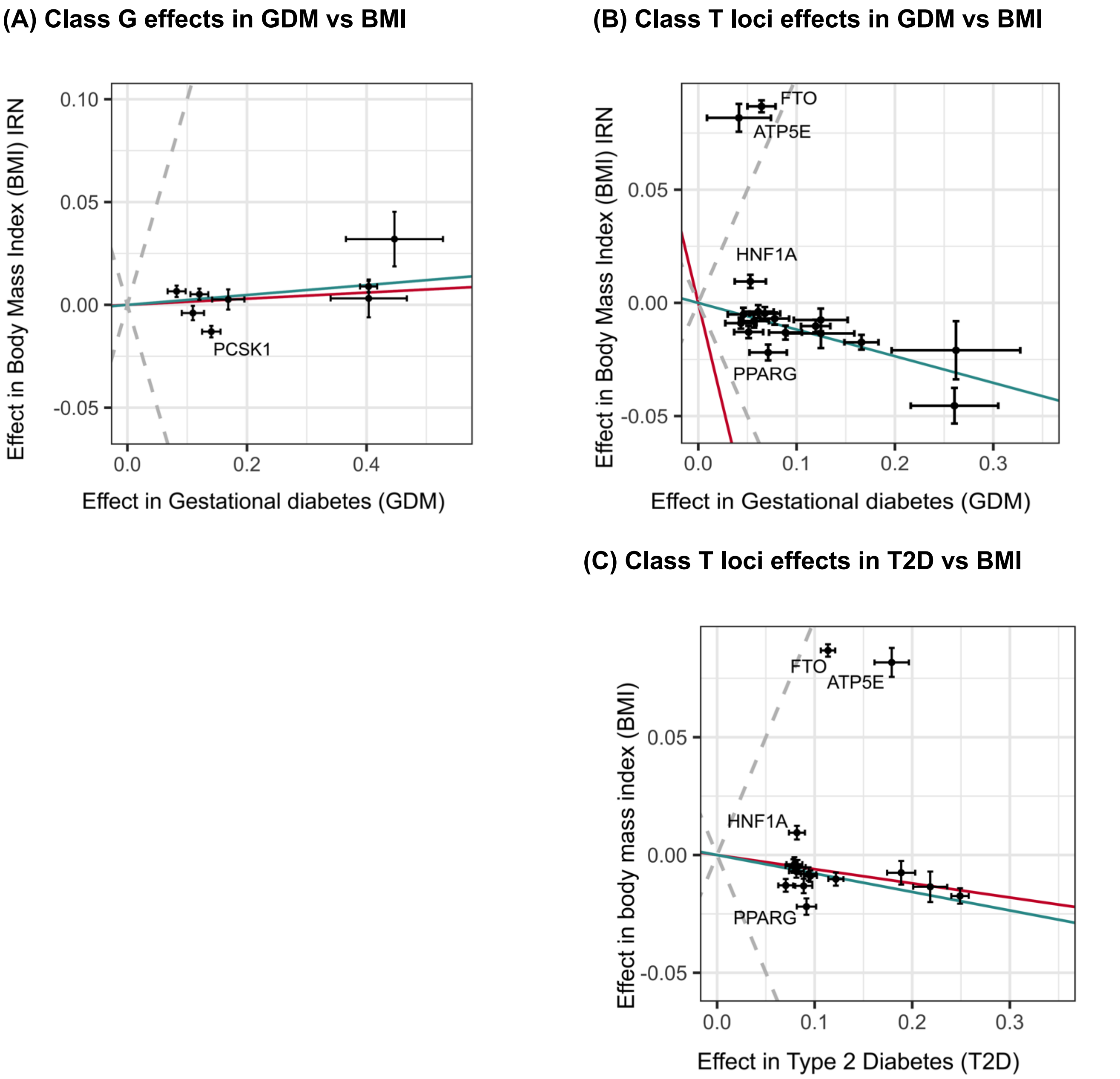

Supplementary Figure 28: Comparison of GDM vs BMI effects sizes by GDM variant class
The Classes of GDM-associated loci were evaluated for their relationship to BMI by comparing the betas (log odds ratios) from the GWAS of GDM (x-axis, 12,332 cases, 131,109 controls) to the betas from GWAS of inverse rank normalized (IRN) BMI in FinnGen (total number 290,820) among (A) ClassG SNPs and (B) ClassT SNPs from the shared variants analysis of GDM and T2D. For comparison, (C) IRN BMI effect sizes are also compared to GWAS of T2D in FinnGen. Error bars indicate +/- 1 standard error in each GWAS. Points labelled with gene names are identified as outliers by SCOUTJOY. Fitted slopes are shown from York regression with all variants (red) and after removal of identified outliers (blue). Dashed gray reference lines indicate equal absolute effect size. Data available in Supplementary Table 31.

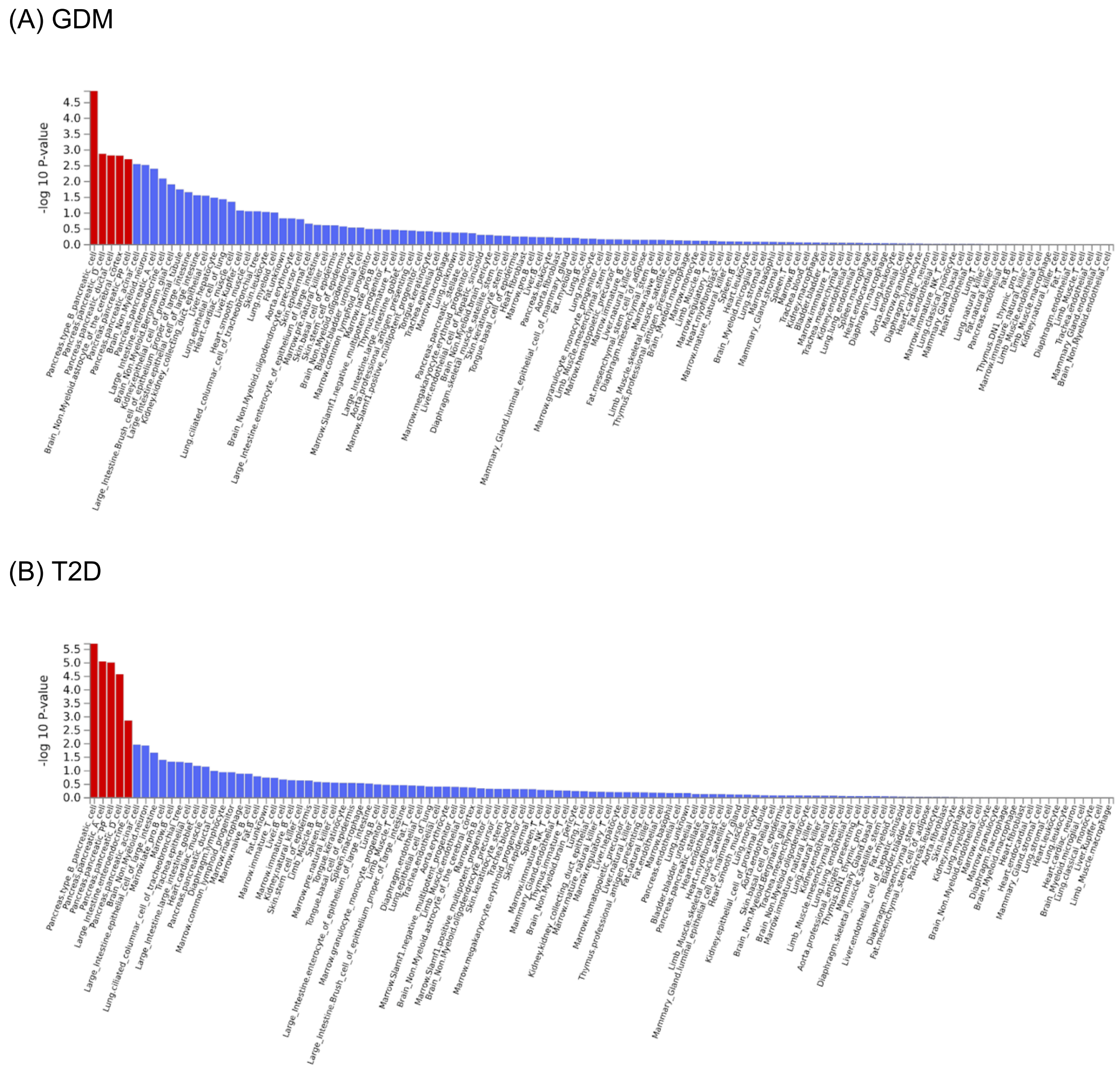

Supplementary Figure 30: Cell type specificity analysis with GDM and T2D summary statistics
FUMA cell specificity analysis v1.3.4 was performed for (A) the current GWAS of GDM and (B) published GWAS meta-analysis of T2D[27]. Unadjusted one-sided p-values from the FUMA cell specificity analysis v1.3.4 from linear regression of MAGMA gene-level results for GDM on cell-type specific gene expression for each cell-tissue pair. Analysis was performed using FUMA GENE2FUNC with murine single cell gene expression data from Tabula Muris. Cell-tissue pairs that remain significant after multiple testing correction are indicated in red. Data is available in Supplementary Table 32.

(A) GDM

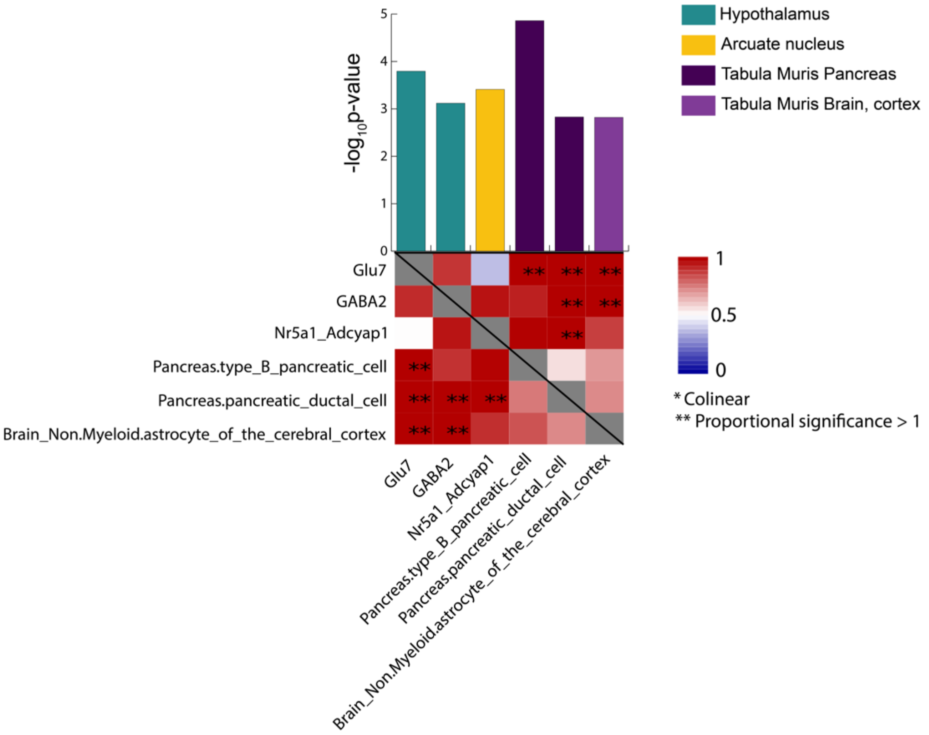

(B) T2D

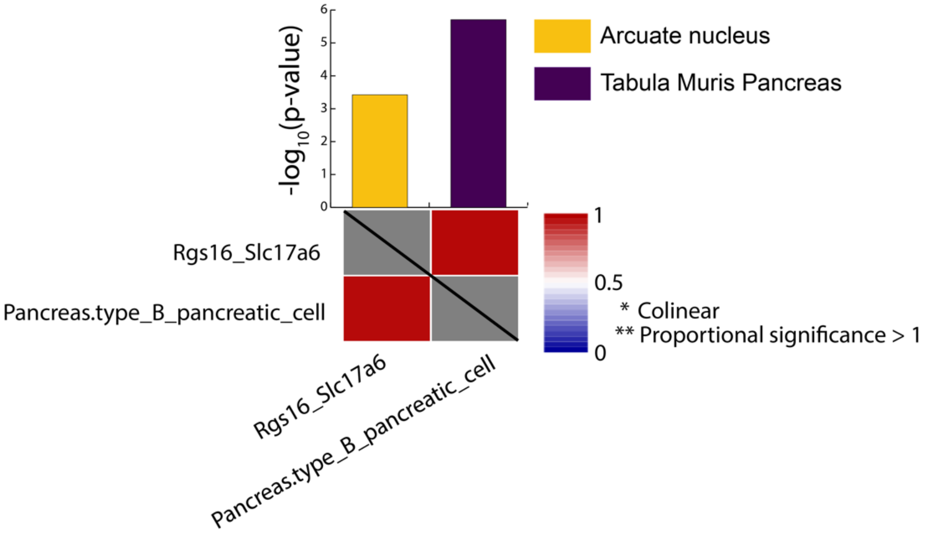

Supplementary Figure 31: Cross dataset conditional analyses reveal independent signals for GDM and for T2D

Evaluation of whether cell type specificity results across single cell expression datasets reflect overlapping genetic signals (Supplementary Note). Barplots indicate unadjusted one-sided p-values from the FUMA cell specificity analysis v1.3.4 from linear regression of MAGMA gene-level associations in the GWAS of (A) GDM or (B) T2D on relative gene expression in the given cell type. Colors indicate RNA-seq dataset source. Results are shown for cell types that are significantly associated with the GWAS after correction for multiple testing and have putatively independent association conditional on other cell types in the same RNA-seq dataset. Heatmaps are asymmetric and indicate the proportional significance (PS) of association with the cell type in the column after conditioning on the cell type in the row. Heatmap cell colors reflect PS between 0 and 1, with PS>1 is

represented by double stars. Analysis was performed using FUMA GENE2FUNC cell specificity analysis step 2 v1.3.4. Data is available in Supplementary Table 33&34.

(A) Mouse pancreas:

(B) Human pancreas:

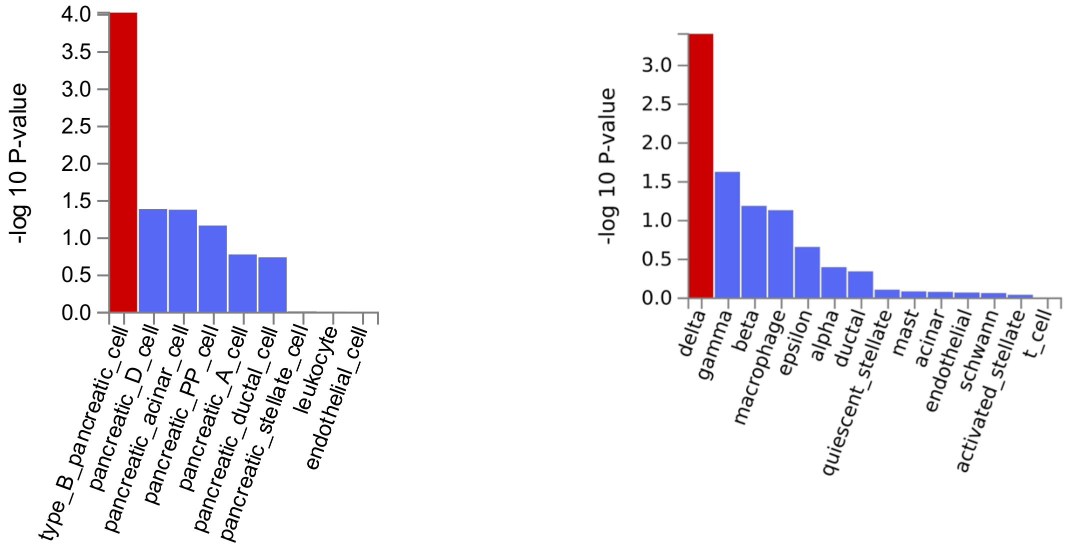

Supplementary Figure 32: Cell type specificity analysis with GDM summary statistics in mouse vs human islets

Cell specificity analysis for GDM GWAS with single cell gene expression data from (A) mouse pancreas in Tabula Muris and (B) a large single cell study of human pancreas[31]. Unadjusted one-sided p-values from the FUMA cell specificity analysis v1.3.4 from linear regression of MAGMA gene-level results for GDM on cell-type specific gene expression for each cell type. Cell types that remain significant after multiple testing correction are indicated in red. Analysis was performed using FUMA GENE2FUNC. Data is available in Supplementary Table 35.

Supplemental References

1. Pervjakova, N., Moen, G.H., Borges, M.C., Ferreira, T., Cook, J.P., Allard, C., Beaumont, R.N., Canouil, M., Hatem, G., Heiskala, A., Joensuu, A., Karhunen, V., Kwak, S.H., Lin, F.T.J., Liu, J., Rifas-Shiman, S., Tam, C.H., Tam, W.H., Thorleifsson, G., Andrew, T., Auvinen, J., Bhowmik, B., Bonnefond, A., Delahaye, F., Demirkan, A., Froguel, P., Haller-Kikkatalo, K., Hardardottir, H., Hummel, S., Hussain, A., Kajantie, E., Keikkala, E., Khamis, A., Lahti, J., Lekva, T., Mustaniemi, S., Sommer, C., Tagoma, A., Tzala, E., Uibo, R., Vaarasmaki, M., Villa, P.M., Birkeland, K.I., Bouchard, L., Duijn, C.M., Finer, S., Groop, L., Hamalainen, E., Hayes, G.M., Hitman, G.A., Jang, H.C., Jarvelin, M.R., Jenum, A.K., Laivuori, H., Ma, R.C., Melander, O., Oken, E., Park, K.S., Perron, P., Prasad, R.B., Qvigstad, E., Sebert, S., Stefansson, K., Steinthorsdottir, V., Tuomi, T., Hivert, M.F., Franks, P.W., McCarthy, M.I., Lindgren, C.M., Freathy, R.M., Lawlor, D.A., Morris, A.P. & Magi, R. Multi-ancestry genome-wide association study of gestational diabetes mellitus highlights genetic links with type 2 diabetes. *Hum Mol Genet* **31**, 3377-3391 (2022).
2. Leitsalu, L., Alavere, H., Tammesoo, M.L., Leego, E. & Metspalu, A. Linking a population biobank with national health registries-the estonian experience. *J Pers Med* **5**, 96-106 (2015).
3. Hormozdiari, F., van de Bunt, M., Segre, A.V., Li, X., Joo, J.W.J., Bilow, M., Sul, J.H., Sankararaman, S., Pasaniuc, B. & Eskin, E. Colocalization of GWAS and eQTL Signals Detects Target Genes. *Am J Hum Genet* **99**, 1245-1260 (2016).
4. Consortium, G.T. The GTEx Consortium atlas of genetic regulatory effects across human tissues. *Science* **369**, 1318-1330 (2020).
5. Kerimov, N., Hayhurst, J.D., Peikova, K., Manning, J.R., Walter, P., Kolberg, L., Samovica, M., Sakthivel, M.P., Kuzmin, I., Trevanion, S.J., Burdett, T., Jupp, S., Parkinson, H., Papatheodorou, I., Yates, A.D., Zerbino, D.R. & Alasoo, K. A compendium of uniformly processed human gene expression and splicing quantitative trait loci. *Nat Genet* **53**, 1290-1299 (2021).
6. Widen, E., Junna, N., Ruotsalainen, S., Surakka, I., Mars, N., Ripatti, P., Partanen, J.J., Aro, J., Mustonen, P., Tuomi, T., Palotie, A., Salomaa, V., Kaprio, J., Partanen, J., Hotakainen, K., Pollanen, P. & Ripatti, S. How Communicating Polygenic and Clinical Risk for Atherosclerotic Cardiovascular Disease Impacts Health Behavior: an Observational Follow-up Study. *Circ Genom Precis Med* **15**, e003459 (2022).
7. Ottensmann, L., Tabassum, R., Ruotsalainen, S.E., Gerl, M.J., Klose, C., Widén, E., FinnGen, Simons, K., Ripatti, S. & Pirinen, M. Genome-wide association analysis of plasma lipidome identifies 495 genetic associations. *medRxiv*, 2023.01.21.23284765 (2023).
8. Laakso, M., Kuusisto, J., Stancakova, A., Kuulasmaa, T., Pajukanta, P., Lusis, A.J., Collins, F.S., Mohlke, K.L. & Boehnke, M. The Metabolic Syndrome in Men study: a resource for studies of metabolic and cardiovascular diseases. *J Lipid Res* **58**, 481-493 (2017).
9. Kramer, C.K., Campbell, S. & Retnakaran, R. Gestational diabetes and the risk of cardiovascular disease in women: a systematic review and meta-analysis. *Diabetologia* **62**, 905-914 (2019).
10. Tobias, D.K., Hu, F.B., Forman, J.P., Chavarro, J. & Zhang, C. Increased risk of hypertension after gestational diabetes mellitus: findings from a large prospective cohort study. *Diabetes Care* **34**, 1582-4 (2011).
11. Echouffo-Tcheugui, J.B., Guan, J., Retnakaran, R. & Shah, B.R. Gestational Diabetes and Incident Heart Failure: A Cohort Study. *Diabetes Care* **44**, 2346-52 (2021).
12. Sridhar, S.B., Xu, F., Darbinian, J., Quesenberry, C.P., Ferrara, A. & Hedderson, M.M. Pregravid liver enzyme levels and risk of gestational diabetes mellitus during a subsequent pregnancy. *Diabetes Care* **37**, 1878-84 (2014).

13. Ryckman, K.K., Spracklen, C.N., Smith, C.J., Robinson, J.G. & Saftlas, A.F. Maternal lipid levels during pregnancy and gestational diabetes: a systematic review and meta-analysis. *BJOG* **122**, 643-51 (2015).
14. Hedderson, M.M., Darbinian, J., Havel, P.J., Quesenberry, C.P., Sridhar, S., Ehrlich, S. & Ferrara, A. Low prepregnancy adiponectin concentrations are associated with a marked increase in risk for development of gestational diabetes mellitus. *Diabetes Care* **36**, 3930-7 (2013).
15. Shahgheibi, S., Farhadifar, F. & Pouya, B. The effect of vitamin D supplementation on gestational diabetes in high-risk women: Results from a randomized placebo-controlled trial. *J Res Med Sci* **21**, 2 (2016).
16. Burris, H.H. & Camargo, C.A., Jr. Vitamin D and gestational diabetes mellitus. *Curr Diab Rep* **14**, 451 (2014).
17. Ravnsborg, T., Andersen, L.L., Trabjerg, N.D., Rasmussen, L.M., Jensen, D.M. & Overgaard, M. First-trimester multimarker prediction of gestational diabetes mellitus using targeted mass spectrometry. *Diabetologia* **59**, 970-9 (2016).
18. Warrington, N.M., Beaumont, R.N., Horikoshi, M., Day, F.R., Helgeland, Ø., Laurin, C., Bacelis, J., Peng, S., Hao, K., Feenstra, B., Wood, A.R., Mahajan, A., Tyrrell, J., Robertson, N.R., Rayner, N.W., Qiao, Z., Moen, G.-H., Vaudel, M., Marsit, C.J., Chen, J., Nodzenski, M., Schnurr, T.M., Zafarmand, M.H., Bradfield, J.P., Grarup, N., Kooijman, M.N., Li-Gao, R., Geller, F., Ahluwalia, T.S., Paternoster, L., Rueedi, R., Huikari, V., Hottenga, J.-J., Lyytikäinen, L.-P., Cavadino, A., Metrustry, S., Cousminer, D.L., Wu, Y., Thiering, E., Wang, C.A., Have, C.T., Vilor-Tejedor, N., Joshi, P.K., Painter, J.N., Ntalla, I., Myhre, R., Pitkänen, N., van Leeuwen, E.M., Joro, R., Lagou, V., Richmond, R.C., Espinosa, A., Barton, S.J., Inskip, H.M., Holloway, J.W., Santa-Marina, L., Estivill, X., Ang, W., Marsh, J.A., Reichetzeder, C., Marullo, L., Hocher, B., Lunetta, K.L., Murabito, J.M., Relton, C.L., Kogevinas, M., Chatzi, L., Allard, C., Bouchard, L., Hivert, M.-F., Zhang, G., Muglia, L.J., Heikkinen, J., Consortium, E.G.G., Morgen, C.S., van Kampen, A.H.C., van Schaik, B.D.C., Mentch, F.D., Langenberg, C., Luan, J.a., Scott, R.A., Zhao, J.H., Hemani, G., Ring, S.M., Bennett, A.J., Gaulton, K.J., Fernandez-Tajes, J., van Zuydam, N.R., Medina-Gomez, C., de Haan, H.G., Rosendaal, F.R., Kutalik, Z., Marques-Vidal, P., Das, S., Willemsen, G., Mbarek, H., Müller-Nurasyid, M., Standl, M., Appel, E.V.R., Fonvig, C.E., Trier, C., van Beijsterveldt, C.E.M., Murcia, M., Bustamante, M., Bonas-Guarch, S., Hougaard, D.M., Mercader, J.M., Linneberg, A., Schraut, K.E., Lind, P.A., Medland, S.E., Shields, B.M., Knight, B.A., Chai, J.-F., Panoutsopoulou, K., Bartels, M., Sánchez, F., Stokholm, J., Torrents, D., Vinding, R.K., Willems, S.M., Atalay, M., Chawes, B.L., Kovacs, P., Prokopenko, I., Tuke, M.A., Yaghootkar, H., Ruth, K.S., Jones, S.E., Loh, P.-R., Murray, A., Weedon, M.N., Tönjes, A., Stumvoll, M., Michaelsen, K.F., Eloranta, A.-M., Lakka, T.A., van Duijn, C.M., Kiess, W., Körner, A., Niinikoski, H., Pahkala, K., Raitakari, O.T., Jacobsson, B., Zeggini, E., Dedoussis, G.V., Teo, Y.-Y., Saw, S.-M., Montgomery, G.W., Campbell, H., Wilson, J.F., Vrijkotte, T.G.M., Vrijheid, M., de Geus, E.J.C.N., Hayes, M.G., Kadarmideen, H.N., Holm, J.-C., Beilin, L.J., Pennell, C.E., Heinrich, J., Adair, L.S., Borja, J.B., Mohlke, K.L., Eriksson, J.G., Widén, E.E., Hattersley, A.T., Spector, T.D., Kähönen, M., Viikari, J.S., Lehtimäki, T., Boomsma, D.I., Sebert, S., Vollenweider, P., Sørensen, T.I.A., Bisgaard, H., Bønnelykke, K., Murray, J.C., Melbye, M., Nohr, E.A., Mook-Kanamori, D.O., Rivadeneira, F., Hofman, A., Felix, J.F., Jaddoe, V.W.V., Hansen, T., Pisinger, C., Vaag, A.A., Pedersen, O., Uitterlinden, A.G., Järvelin, M.-R., Power, C., Hyppönen, E., Scholtens, D.M., Lowe, W.L., Davey Smith, G., Timpson, N.J., Morris, A.P., Wareham, N.J., Hakonarson, H., Grant, S.F.A., Frayling, T.M., Lawlor, D.A., Njølstad, P.R., Johansson, S., Ong, K.K., McCarthy, M.I., Perry, J.R.B., Evans, D.M. & Freathy, R.M. Maternal and fetal genetic effects on birth weight and their relevance to cardio-metabolic risk factors. *Nature Genetics* **51**, 804-814 (2019).
19. York, D. Least squares fitting of a straight line with correlated errors. *Earth and Planetary Science Letters* **5**, 320-324 (1968).

20. York, D., Evensen, N.M., Martínez, M.L. & Delgado, J.D.B. Unified equations for the slope, intercept, and standard errors of the best straight line. *American Journal of Physics* **72**, 367-375 (2004).
21. Mahon, K.I. The New "York" Regression: Application of an Improved Statistical Method to Geochemistry. *International Geology Review* **38**, 293-303 (1996).
22. Verbanck, M., Chen, C.-Y., Neale, B. & Do, R. Detection of widespread horizontal pleiotropy in causal relationships inferred from Mendelian randomization between complex traits and diseases. *Nature Genetics* **50**, 693-698 (2018).
23. Titterington, D.M. & Halliday, A.N. On the fitting of parallel isochrons and the method of maximum likelihood. *Chemical Geology* **26**, 183-195 (1979).
24. Turley, P., Walters, R.K., Maghzian, O., Okbay, A., Lee, J.J., Fontana, M.A., Nguyen-Viet, T.A., Wedow, R., Zacher, M., Furlotte, N.A., Agee, M., Alipanahi, B., Auton, A., Bell, R.K., Bryc, K., Elson, S.L., Fontanillas, P., Furlotte, N.A., Hinds, D.A., Hromatka, B.S., Huber, K.E., Kleinman, A., Litterman, N.K., McIntyre, M.H., Mountain, J.L., Northover, C.A.M., Sathirapongsasuti, J.F., Sazonova, O.V., Shelton, J.F., Shringarpure, S., Tian, C., Tung, J.Y., Vacic, V., Wilson, C.H., Pitts, S.J., Magnusson, P., Oskarsson, S., Johannesson, M., Visscher, P.M., Laibson, D., Cesarini, D., Neale, B.M., Benjamin, D.J., andMe Research, T. & Social Science Genetic Association, C. Multi-trait analysis of genome-wide association summary statistics using MTAG. *Nature Genetics* **50**, 229-237 (2018).
25. Martin, J., Khramtsova, E.A., Goleva, S.B., Blokland, G.A.M., Traglia, M., Walters, R.K., Hubel, C., Coleman, J.R.I., Breen, G., Borglum, A.D., Demontis, D., Grove, J., Werge, T., Bralten, J., Bulik, C.M., Lee, P.H., Mathews, C.A., Peterson, R.E., Winham, S.J., Wray, N., Edenberg, H.J., Guo, W., Yao, Y., Neale, B.M., Faraone, S.V., Petryshen, T.L., Weiss, L.A., Duncan, L.E., Goldstein, J.M., Smoller, J.W., Stranger, B.E., Davis, L.K. & Sex Differences Cross-Disorder Analysis Group of the Psychiatric Genomics, C. Examining Sex-Differentiated Genetic Effects Across Neuropsychiatric and Behavioral Traits. *Biol Psychiatry* **89**, 1127-1137 (2021).
26. Karaderi, T., Drong, A.W. & Lindgren, C.M. Insights into the Genetic Susceptibility to Type 2 Diabetes from Genome-Wide Association Studies of Obesity-Related Traits. *Current Diabetes Reports* **15**, 83 (2015).
27. Mahajan, A., Taliun, D., Thurner, M., Robertson, N.R., Torres, J.M., Rayner, N.W., Payne, A.J., Steinthorsdottir, V., Scott, R.A., Grarup, N., Cook, J.P., Schmidt, E.M., Wuttke, M., Sarnowski, C., Magi, R., Nano, J., Gieger, C., Trompet, S., Lecoeur, C., Preuss, M.H., Prins, B.P., Guo, X., Bielak, L.F., Below, J.E., Bowden, D.W., Chambers, J.C., Kim, Y.J., Ng, M.C.Y., Petty, L.E., Sim, X., Zhang, W., Bennett, A.J., Bork-Jensen, J., Brummett, C.M., Canouil, M., Ec Kardt, K.U., Fischer, K., Kardia, S.L.R., Kronenberg, F., Lall, K., Liu, C.T., Locke, A.E., Luan, J., Ntalla, I., Nylander, V., Schonherr, S., Schurmann, C., Yengo, L., Bottinger, E.P., Brandslund, I., Christensen, C., Dedoussis, G., Florez, J.C., Ford, I., Franco, O.H., Frayling, T.M., Giedraitis, V., Hackinger, S., Hattersley, A.T., Herder, C., Ikram, M.A., Ingelsson, M., Jorgensen, M.E., Jorgensen, T., Kriebel, J., Kuusisto, J., Ligthart, S., Lindgren, C.M., Linneberg, A., Lyssenko, V., Mamakou, V., Meitinger, T., Mohlke, K.L., Morris, A.D., Nadkarni, G., Pankow, J.S., Peters, A., Sattar, N., Stancakova, A., Strauch, K., Taylor, K.D., Thorand, B., Thorleifsson, G., Thorsteinsdottir, U., Tuomilehto, J., Witte, D.R., Dupuis, J., Peyser, P.A., Zeggini, E., Loos, R.J.F., Froguel, P., Ingelsson, E., Lind, L., Groop, L., Laakso, M., Collins, F.S., Jukema, J.W., Palmer, C.N.A., Grallert, H., Metspalu, A., Dehghan, A., Kottgen, A., Abecasis, G.R., Meigs, J.B., Rotter, J.I., Marchini, J., Pedersen, O., Hansen, T., Langenberg, C., Wareham, N.J., Stefansson, K., Gloyn, A.L., Morris, A.P., Boehnke, M. & McCarthy, M.I. Fine-mapping type 2 diabetes loci to single-variant resolution using high-density imputation and islet-specific epigenome maps. *Nat Genet* **50**, 1505-1513 (2018).
28. Tabula Muris Consortium. Single-cell transcriptomics of 20 mouse organs creates a Tabula Muris. *Nature* **562**, 367-372 (2018).
29. Chen, R., Wu, X., Jiang, L. & Zhang, Y. Single-Cell RNA-Seq Reveals Hypothalamic Cell Diversity. *Cell Rep* **18**, 3227-3241 (2017).

30. Campbell, J.N., Macosko, E.Z., Fenselau, H., Pers, T.H., Lyubetskaya, A., Tenen, D., Goldman, M., Verstegen, A.M., Resch, J.M., McCarroll, S.A., Rosen, E.D., Lowell, B.B. & Tsai, L.T. A molecular census of arcuate hypothalamus and median eminence cell types. *Nat Neurosci* **20**, 484-496 (2017).
31. Baron, M., Veres, A., Wolock, S.L., Faust, A.L., Gaujoux, R., Vetere, A., Ryu, J.H., Wagner, B.K., Shen-Orr, S.S., Klein, A.M., Melton, D.A. & Yanai, I. A Single-Cell Transcriptomic Map of the Human and Mouse Pancreas Reveals Inter- and Intra-cell Population Structure. *Cell Syst* **3**, 346-360 e4 (2016).
32. Watanabe, K., Umicevic Mirkov, M., de Leeuw, C.A., van den Heuvel, M.P. & Posthuma, D. Genetic mapping of cell type specificity for complex traits. *Nat Commun* **10**, 3222 (2019).
33. Ladyman, S.R. & Grattan, D.R. Region-Specific Suppression of Hypothalamic Responses to Insulin To Adapt to Elevated Maternal Insulin Secretion During Pregnancy. *Endocrinology* **158**, 4257-4269 (2017).
34. He, Y., Xu, P., Wang, C., Xia, Y., Yu, M., Yang, Y., Yu, K., Cai, X., Qu, N., Saito, K., Wang, J., Hyseni, I., Robertson, M., Piyarathna, B., Gao, M., Khan, S.A., Liu, F., Chen, R., Coarfa, C., Zhao, Z., Tong, Q., Sun, Z. & Xu, Y. Estrogen receptor-alpha expressing neurons in the ventrolateral VMH regulate glucose balance. *Nat Commun* **11**, 2165 (2020).
35. Yang, J.A., Stires, H., Belden, W.J. & Roepke, T.A. The Arcuate Estrogen-Regulated Transcriptome: Estrogen Response Element-Dependent and -Independent Signaling of ERalpha in Female Mice. *Endocrinology* **158**, 612-626 (2017).
36. Dirice, E., De Jesus, D.F., Kahraman, S., Basile, G., Ng, R.W., El Ouaamari, A., Teo, A.K.K., Bhatt, S., Hu, J. & Kulkarni, R.N. Human duct cells contribute to beta cell compensation in insulin resistance. *JCI Insight* **4**(2019).
37. Dirice, E., Basile, G., Kahraman, S., Diegisser, D., Hu, J. & Kulkarni, R.N. Single-nucleus RNA-Seq reveals singular gene signatures of human ductal cells during adaptation to insulin resistance. *JCI Insight* **7**(2022).
