# Supplementary material for: Distinct and shared genetic architectures of gestational diabetes mellitus and type 2 diabetes
Source: Nat Genet. 2024 Jan 5;56(3):377–82. doi: 10.1038/s41588-023-01607-4 (PMC10937370; doi:10.1038/s41588-023-01607-4)
Supplement: Supplementary file 3 — Supplementary Tables 1–35. [file 41588_2023_1607_MOESM3_ESM.pdf]

nature portfolio

Corresponding author(s): Mark Daly and Elisabeth Widén

Last updated by author(s): Nov 6, 2023

Reporting Summary

Nature Portfolio wishes to improve the reproducibility of the work that we publish. This form provides structure for consistency and transparency in reporting. For further information on Nature Portfolio policies, see our Editorial Policies and the Editorial Policy Checklist.

Statistics

For all statistical analyses, confirm that the following items are present in the figure legend, table legend, main text, or Methods section.

n/a	Confirmed	
☐	☒	The exact sample size (*n*) for each experimental group/condition, given as a discrete number and unit of measurement
☐	☒	A statement on whether measurements were taken from distinct samples or whether the same sample was measured repeatedly
☐	☒	The statistical test(s) used AND whether they are one- or two-sided<br>*Only common tests should be described solely by name; describe more complex techniques in the Methods section.*
☐	☒	A description of all covariates tested
☐	☒	A description of any assumptions or corrections, such as tests of normality and adjustment for multiple comparisons
☐	☒	A full description of the statistical parameters including central tendency (e.g. means) or other basic estimates (e.g. regression coefficient) AND variation (e.g. standard deviation) or associated estimates of uncertainty (e.g. confidence intervals)
☐	☒	For null hypothesis testing, the test statistic (e.g. *F*, *t*, *r*) with confidence intervals, effect sizes, degrees of freedom and *P* value noted<br>*Give P values as exact values whenever suitable.*
☐	☒	For Bayesian analysis, information on the choice of priors and Markov chain Monte Carlo settings
☐	☒	For hierarchical and complex designs, identification of the appropriate level for tests and full reporting of outcomes
☐	☒	Estimates of effect sizes (e.g. Cohen's *d*, Pearson's *r*), indicating how they were calculated

Our web collection on statistics for biologists contains articles on many of the points above.

Software and code

Policy information about availability of computer code

Data collection	No software was used for data collection.
Data analysis	GWAs was performed using REGENIE 2.24 (https://rgcgithub.github.io/regenie/) with the pipeline described at the github for FinnGen GWAS pipeline publicly described (https://github.com/FINNGEN/regenie-pipelines).<br><br>Finemapping of GWAS signals was performed using SuSIE 0.9.2 (https://stephenslab.github.io/susieR/index.html) with the FinnGen finemapping pipeline publicly described (https://github.com/FINNGEN/finemapping-pipeline)<br><br>Colocalization was performed using the FinnGen colocalization pipline which is based on eCAVIAR (https://github.com/FINNGEN/pheweb-colocalization). Annotations were performed according to the FinnGen annotation pipeline (https://github.com/FINNGEN/autoreporting). Genetic correlation was performed using LDSC v1.0.1(https://github.com/bulik/ldsc).<br><br>Gene level association results from MAGMA were used to identify tissues and pathways enrichment using the FUMA modules for SNP2GENE v1.3.5d, GENE2FUNC v1.3.4and cell specificity module v1.3.4 (https://fuma.ctglab.nl/).<br><br>Comparison of heterogeneity of cross-disorder genetic effects was performed using Significant Cross-trait OUtliers and Trends in JOint York regression (SCOUTJOY) v0.0.5 (https://github.com/aelliott08/SCOUTJOY). Classification of variants based on their bivariate effect size was carried out using Linemodels 0.2.0 (https://github.com/mjpirinen/linemodels).

For manuscripts utilizing custom algorithms or software that are central to the research but not yet described in published literature, software must be made available to editors and reviewers. We strongly encourage code deposition in a community repository (e.g. GitHub). See the Nature Portfolio guidelines for submitting code & software for further information.

Data

Policy information about availability of data

All manuscripts must include a data availability statement. This statement should provide the following information, where applicable:

- Accession codes, unique identifiers, or web links for publicly available datasets
- A description of any restrictions on data availability
- For clinical datasets or third party data, please ensure that the statement adheres to our policy

The FinnGen data may be accessed through Finnish Biobanks' FinnBB portal (www.finbb.fi) and THL Biobank data through THL Biobank (https://thl.fi/en/web/thl-biobank). The full summary statistics of the primary scan of GDM are available at https://www.ebi.ac.uk/gwas/studies/GCST90296696. GWAS of T2D in males, females, parous females, and nulliparous females are available at https://www.ebi.ac.uk/gwas/studies/GCST90296697, https://www.ebi.ac.uk/gwas/studies/GCST90296698, https://www.ebi.ac.uk/gwas/studies/GCST90296699, https://www.ebi.ac.uk/gwas/studies/GCST90296700.

Additional data used for colocalization/annotations is available from GWAS Catalog (https://www.ebi.ac.uk/gwas/home), GTEx (https://gtexportal.org/home/datasets), EMBL-EPI eQTL Catalogue (https://www.ebi.ac.uk/eqtl/), UK Biobank fine-mapping (https://www.finucanelab.org/data), and METSIM (https://pheweb.org/metsim-metab/). GeneRisk lipid QTL results will be available on GWAS Catalog (https://www.ebi.ac.uk/gwas/) upon publication of the corresponding manuscript (https://www.medrxiv.org/content/10.1101/2023.01.21.23284765v1.full). A complete list of sources used for annotation with FUMA, including download links, are available at https://fuma.ctglab.nl/links and https://fuma.ctglab.nl/tutorial#datasets; see Supplementary Note for details on datasets used in the current analysis. Previous GWAS results on T2D from Mahajan et al. 2018 are available from the DIAGRAM Consortium (https://diagram-consortium.org/downloads.html). GWAS results for glycemic traits are available from MAGIC (https://magicinvestigators.org/downloads/). Additional GWAS results used for genetic correlation analyses are also all publicly available: birth weight results from the EGG Consortium (https://egg-consortium.org/birth-weight-2019.html), BMI, height, and WHR results from GIANT (https://portals.broadinstitute.org/collaboration/giant/index.php/GIANT_consortium_data_files), liver fat, NAFLD, T1D, and number of children results on GWAS Catalog (https://www.ebi.ac.uk/gwas/; GCST90029073, GCST008468, GCST90014023, and GCST90029038, respectively), hypertension results on NHLBI GRASP (https://grasp.nhlbi.nih.gov/FullResults.aspx), CAD, heart failure, and AtrialFib results from the CVD Knowledge Portal (https://cvd.hugeamp.org/downloads.html#summary), and Sinnott-Armstrong et al. biomaker results on FigShare (https://nih.figshare.com/articles/dataset/The_meta-analyzed_GWAS_summary_statistics_for_35_lab_biomarkers_described_in_Genetics_of_35_blood_and_urine_biomarkers_in_the_UK_Biobank_/12355382).

Human research participants

Policy information about studies involving human research participants and Sex and Gender in Research.

Reporting on sex and gender	The current study investigates the genetic underpinnings of pregnancy-induced diabetes. Therefore the main analyses only includes women who have delivered a child. Data on sex, pregnancies and childbirth were obtained from national care and population registers. Data on gender were not available.
Population characteristics	All study subjects were of Finnish ancestry and participants from the FinnGen study, which is a biobank project aiming to collect and analyse genome and health data from 500,000 Finnish biobank participants. (Kurki et al. Nature 613, 508–518 (2023)). The current study used the results from the FinnGen release R8, which includes data from 342,499 individuals (mean age 51.4 yrs). Our analyses encompass 143,441 parous women. 12,332 were cases with a medical history of gestational diabetes and 131,109 were controls. Information on delivery was obtained from the Finnish Population Register or the Medical Birth Register. Cases with gestational diabetes were identified based on receiving a diagnosis of glucose intolerance or diabetes during pregnancy with no other diabetes codes prior to pregnancy.<br><br>FinnGen participants have been genotyped with Illumina (Illumina) and Affymetrix (Thermo Fisher Scientific) chip arrays and genotype calls were made with GenCall and zCall algorithms for Illumina and the AxiomGT1 algorithm for Affymetrix data. . Imputation was performed using population-specific SISu v3 importation reference panel.
Recruitment	The FinnGen Study is a biobank project that utilizes samples from a nationwide network of Finnish biobanks and electronic health records from national registries. All FinnGen participants provided informed consent for biobank research, based on the Finnish Biobank Act. Alternatively, separate research cohorts, collected prior the Finnish Biobank Act came into effect (in September 2013) and start of FinnGen (August 2017), were collected based on study-specific consents and later transferred to the Finnish biobanks after approval by Fimea, the National Supervisory Authority for Welfare and Health.
Ethics oversight	The study protocol was approved by the Coordinating Ethics Committee of the Hospital District of Helsinki and Uusimaa (HUS).

Note that full information on the approval of the study protocol must also be provided in the manuscript.

Field-specific reporting

Please select the one below that is the best fit for your research. If you are not sure, read the appropriate sections before making your selection.

☒ Life sciences   ☐ Behavioural & social sciences   ☐ Ecological, evolutionary & environmental sciences

For a reference copy of the document with all sections, see nature.com/documents/nr-reporting-summary-flat.pdf

Life sciences study design

All studies must disclose on these points even when the disclosure is negative.

Sample size	The current study includes 12,332 cases with gestational diabetes and 131,109 controls (among women who had given birth). Data obtained from population and Birth Registry records enabled us to identify all parous women included in FinnGen data release R8 who had given birth to a child. No Health record data were extensively analyzed to identify all cases with medical records of glucose intolerance of diabetes during pregnancy. Cases were confirmed to have a diagnosis within a pregnancy window and those with diagnoses of diabetes prior to the index pregnancy were excluded. All available samples were included to maximize the statistical power for discovery. The exact sample size was not predetermined by any other criteria than availability of samples.
Data exclusions	To ensure that only women women with pregnancy-induced diabetes were included the analyses we excluded women with diagnoses of diabetes prior to the index pregnancy . Also, women with a medical record of significant pancreatic disease prior to pregnancy potentially causing glucose intolerance, i.e., chronic pancreatitis, pancreatic necrosis, pancreatic cancer, cystic fibrosis were excluded.
Replication	Replication analysis has been carried out in an independent data set from FinnGen encompassing 6,026 women with GDM and 45,296 contral and in a data set from Estonian biobank including 2,904 GDM cases and 125,513 controls. Eleven of 13 associations replicated whereas two loci were not significant in the replication sets albeit directionally consistent.
Randomization	Our study is a case-control study and randomization is not applicable.
Blinding	Our study is a case-control study and blinding is not applicable.

Reporting for specific materials, systems and methods

We require information from authors about some types of materials, experimental systems and methods used in many studies. Here, indicate whether each material, system or method listed is relevant to your study. If you are not sure if a list item applies to your research, read the appropriate section before selecting a response.

Materials & experimental systems

n/a	Involved in the study	
☒	☐	Antibodies
☒	☐	Eukaryotic cell lines
☒	☐	Palaeontology and archaeology
☒	☐	Animals and other organisms
☒	☐	Clinical data
☒	☐	Dual use research of concern

Methods

n/a	Involved in the study	
☒	☐	ChIP-seq
☒	☐	Flow cytometry
☒	☐	MRI-based neuroimaging
